# Human cells contain myriad excised linear intron RNAs with links to gene regulation and potential utility as biomarkers

**DOI:** 10.1371/journal.pgen.1011416

**Published:** 2024-09-26

**Authors:** Jun Yao, Hengyi Xu, Elizabeth A. Ferrick-Kiddie, Ryan M. Nottingham, Douglas C. Wu, Manuel Ares, Alan M. Lambowitz

**Affiliations:** 1 Departments of Molecular Biosciences and Oncology University of Texas at Austin Austin, Texas, United States of America; 2 Department of Molecular, Cell, and Developmental Biology University of California, Santa Cruz, California, United States of America; Ohio State University, UNITED STATES OF AMERICA

## Abstract

A previous study using Thermostable Group II Intron Reverse Transcriptase sequencing (TGIRT-seq) found human plasma contains short (≤300 nt) structured full-length excised linear intron (FLEXI) RNAs with potential to serve as blood-based biomarkers. Here, TGIRT-seq identified >9,000 different FLEXI RNAs in human cell lines, including relatively abundant FLEXIs with cell-type-specific expression patterns. Analysis of public CLIP-seq datasets identified 126 RNA-binding proteins (RBPs) that have binding sites within the region corresponding to the FLEXI or overlapping FLEXI splice sites in pre-mRNAs, including 53 RBPs with binding sites for ≥30 different FLEXIs. These included splicing factors, transcription factors, a chromatin remodeling protein, cellular growth regulators, and proteins with cytoplasmic functions. Analysis of ENCODE datasets identified subsets of these RBPs whose knockdown impacted FLEXI host gene mRNA levels or proximate alternative splicing, indicating functional interactions. Hierarchical clustering identified six subsets of RBPs whose FLEXI binding sites were co-enriched in six subsets of functionally related host genes: AGO1-4 and DICER, including but not limited to agotrons or mirtron pre-miRNAs; DKC1, NOLC1, SMNDC1, and AATF (Apoptosis Antagonizing Transcription Factor), including but not limited to snoRNA-encoding FLEXIs; two subsets of alternative splicing factors; and two subsets that included RBPs with cytoplasmic functions (*e*.*g*., LARP4, PABPC4, METAP2, and ZNF622) together with regulatory proteins. Cell fractionation experiments showed cytoplasmic enrichment of FLEXI RNAs with binding sites for RBPs with cytoplasmic functions. The subsets of host genes encoding FLEXIs with binding sites for different subsets of RBPs were co-enriched with non-FLEXI other short and long introns with binding sites for the same RBPs, suggesting overarching mechanisms for coordinately regulating expression of functionally related genes. Our findings identify FLEXIs as a previously unrecognized large class of cellular RNAs and provide a comprehensive roadmap for further analyzing their biological functions and the relationship of their RBPs to cellular regulatory mechanisms.

## Author summary

We characterized a previously unrecognized, large class of full-length excised liner intron (FLEXI) RNAs that are ≤300 nucleotides in length and relatively stable in human cells and plasma. These included subsets of FLEXIs with binding sites for different subsets of RNA-binding proteins (RBPs) that have different cellular functions and are co-enriched in subsets of functionally related genes with other short and long introns with binding sites for the same RBPs, potentially enabling coordinate regulation of different gene sets. Many of the proteins that have binding sites in different subsets of FLEXIs were also identified as interacting with each other by STRING protein-network analysis, potentially enabling co-localization of subsets of proteins to different intracellular locations, including the cytoplasm. Our findings provide a comprehensive road map for further analysis of the functions of FLEXIs and their associated RBPs and for the identification of FLEXIs that can serve as biomarkers in human cells and plasma.

## Introduction

Most protein-coding genes in eukaryotes consist of exons separated by introns, which are removed by RNA splicing to produce mRNAs. RNA splicing is performed by the spliceosome, a large ribonucleoprotein complex that catalyzes transesterification reactions yielding ligated exons and an excised intron lariat RNA, whose 5’ end is linked to a branch-point nucleotide near its 3’ end by a 2’-5’-phosphodiester bond [1]. After splicing, this bond is typically hydrolyzed by the debranching enzyme DBR1, and the resulting linearized intron RNA is rapidly degraded by exonucleases [2]. Several classes of excised intron RNAs have been found to persist after excision. These include trimmed lariat RNAs that lack a 3’ tail; circularized intron RNAs whose 5’ and 3’ nucleotides are putatively linked by a 3’-5’ phosphodiester bond; and full-length excised linear intron RNAs (FLEXIs), with members of all 3 classes having cellular functions or clinical correlations [3–14]. FLEXIs include a group of yeast excised linear intron RNAs that contribute to cell growth regulation by sequestering spliceosomal proteins in stationary phase or under other stress conditions [11,15]. Other FLEXIs include mirtron pre-miRNAs, structured excised intron RNAs that are debranched by DBR1 and processed by DICER into annotated miRNAs, and agotrons, structured excised intron RNAs that bind AGO2 and repress target mRNAs in a miRNA-like manner [16–19].

Previously, we identified 44 different short (≤300 nt) FLEXI RNAs in commercial human plasma from healthy individual by using Thermostable Group II Intron Reverse Transcriptase sequencing (TGIRT-seq), a method that enables continuous end-to-end sequencing reads of structured RNAs [20]. Plasma FLEXIs were identified by peak calling as discrete blocks of continuous sequencing reads corresponding to excised intron RNAs beginning and ending within 3 nucleotides (nt) of annotated splice sites, with more than half corresponding to annotated agotrons or mirtron pre-miRNAs [20]. Almost all of the plasma FLEXIs (>95%) had stable predicted secondary structures (ΔG ≤ -30 kcal/mol), with ~50% containing a CLIP-seq-identified binding site for one or more RNA-binding proteins (RBPs) that may help protect FLEXIs from plasma RNases. These findings suggested that FLEXIs might have utility as RNA biomarkers in liquid biopsies [20].

Despite the precedents of agotrons and mirtron pre-miRNAs, human FLEXIs have remained largely unexplored. Here, TGIRT-seq of RNAs in human cell lines identified >9,000 FLEXIs, subsets of which have cell-type specific expression patterns. Analysis of published CLIP-seq datasets identified subsets of RBPs with binding sites for FLEXIs that were enriched in different subsets of functionally related host genes with other short and long intron that have binding sites for the same RBPs, suggesting the involvement of these RBPs in previously unknown overarching cellular regulatory mechanism.

## Results

### Identification of FLEXIs in cellular RNA preparations

**Fig 1:**
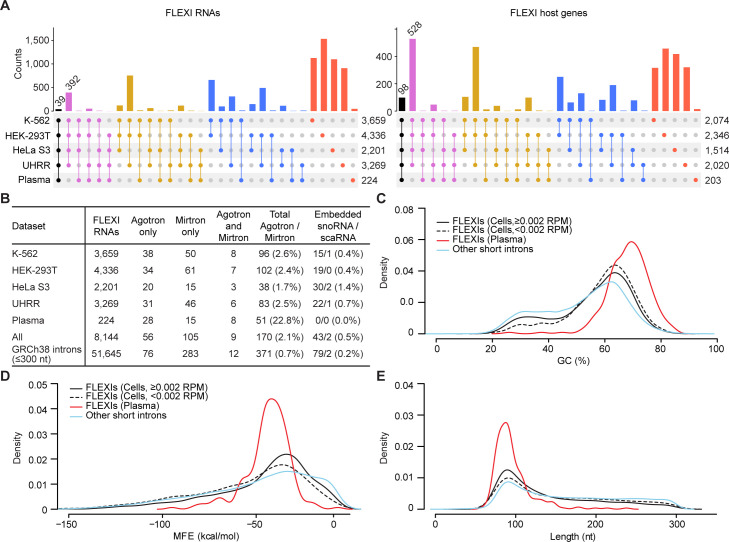
Characteristics of FLEXIs in human cells and plasma. (A) UpSet plots of FLEXI RNAs and their host genes identified by TGIRT-seq of RNA preparations from the indicated human cell lines and plasma. Different FLEXIs from the same host gene were aggregated into one entry for that gene in the plot. (B) Numbers and percentages of different FLEXIs in datasets for each sample type that correspond to an annotated agotron, a pre-miRNA of an annotated mirtron, or encode an annotated snoRNA, including Cajal body-specific snoRNAs (scaRNAs). "All" indicates the number and percentages of different FLEXIs in each category in this group of samples, and GRCh38 indicates the number and percentages of all 51,645 annotated short introns (≤300 nt) in each category in the GRCh38 human genome reference sequence. (C-E) Density distribution plots of (C) GC content, (D) minimum free energy (MFE) for the most stable RNA secondary structure predicted by RNAfold, and (E) length for different categories of FLEXIs compared to other GRCh38-annotated short introns in a merged dataset for the 4 cellular RNA samples.

A search of the human genome (Ensembl GRCh38 Release 93 annotations; http://www.ensembl.org) identified 51,645 short introns (≤300 nt) in 12,020 different genes that could potentially give rise to FLEXIs. To investigate which short introns give rise to FLEXIs, we carried out TGIRT-seq [21,22] of rRNA-depleted, unfragmented HEK-293T, K-562, and HeLa S3 whole-cell RNAs and Universal Human Reference RNA (UHRR), a commercial mixture of whole-cell RNAs from 10 human cell lines (Fig 1). TGIRT-seq is well-suited for the identification of FLEXIs as it employs a TGIRT enzyme (GsI-IIC RT aka TGIRT-III) that can efficiently template-switch from an RNA-seq adapter to initiate cDNA synthesis directly at the 3’-nucleotide of target RNAs and then reverse transcribe processively to yield full-length cDNAs that give end-to-end sequencing reads of structured RNAs [23–26]. Most of the reads in the TGIRT-seq datasets of unfragmented whole-cell RNAs corresponded to full-length mature tRNAs and other small non-coding RNAs (sncRNAs; S1 and S2 Figs).

To identify FLEXIs in the whole-cell RNA preparations, we compiled the coordinates of all short introns (≤300 nt) in Ensembl GRCh38 Release 93 annotations into a BED file and searched for intersections in TGIRT-seq datasets for each of the 4 cellular RNA samples. For each cellular RNA sample, the searched TGIRT-seq dataset included merged technical replicates totaling 666 to 768 million mapped paired-end reads, supplemented by datasets for previously published biological replicates to assess reproducibility (S1 Table). As done previously for FLEXI RNAs in plasma, we identified cellular FLEXI RNAs as continuous intron reads that began and ended within 3 nt of annotated splice sites [20]. This definition enabled us to include full-length intron reads with small numbers of non-templated nucleotides added by cellular enzymes to the 3’ end of RNAs [27] or by the TGIRT enzyme to the 3’ end of completed cDNAs [21,22]. The 300-nt size cutoff for more detailed characterization of FLEXIs was based on the length distribution of FLEXIs found in human plasma [20] and encompassed 85–92% of the FLEXIs detected in the 4 cellular RNA samples, including the most abundant FLEXIs (S3A Fig). By using these methods, we initially identified 8,144 different FLEXIs originating from 3,743 different protein-coding genes, lncRNA genes, or pseudogenes (collectively denoted FLEXI host genes). By using the same updated read mapping pipeline and mapping reads to the same human genome reference sequence, we identified 224 FLEXIs in the previously analyzed human plasma samples (S2 Table) [20].

The distribution of FLEXIs and their host genes between the different cellular RNA samples and plasma is shown in UpSet plots in Fig 1A. Fifty-six of the identified FLEXIs corresponded to an annotated agotron and 105 corresponded to a pre-miRNA of an annotated mirtron (Fig 1B) [16,19]. The percentage of FLEXIs corresponding to annotated agotrons or mirtron pre-miRNAs was higher in plasma (22.8%) than in cells (1.7–2.6%; p <1×10^−15^ by Fisher’s exact test for the different cell types tested; Fig 1B), suggesting preferential cellular export and/or greater resistance of these classes of FLEXIs to plasma RNases [20]. Forty-three of the FLEXIs in the cellular RNA samples encoded an annotated snoRNA, all of which were also detected as processed mature snoRNAs in the same samples (Fig 1B).

Density distribution plots showed that the identified FLEXIs differed from other short introns in being skewed toward higher GC content and more stable predicted secondary structures (*i*.*e*., lower minimum free energy (MFE) for the most stable secondary structure predicted by RNA fold; p≤0.001 for both by Wilcoxon signed-rank test; Fig 1C and 1D). These differences were most pronounced for the FLEXIs found in plasma, which were a relatively homogeneous subset with peaks at approximately 90-nt length, 70% GC content, and -40 kcal/mol MFE (Fig 1C–1E). Lower and higher abundance FLEXIs in the cellular RNA samples (<0.002 or ≥0.002 reads per million (RPM), respectively) had similar GC contents and MFEs that differed from those of other short introns (Fig 1C and 1D, respectively). Collectively, these findings showed that human cells contain large numbers of previously unidentified FLEXI RNAs, only small subsets of which corresponded to annotated agotrons or mirtron pre-miRNAs or were detected in plasma [16,19].

### FLEXIs are excised linear intron RNAs

**Fig 2:**
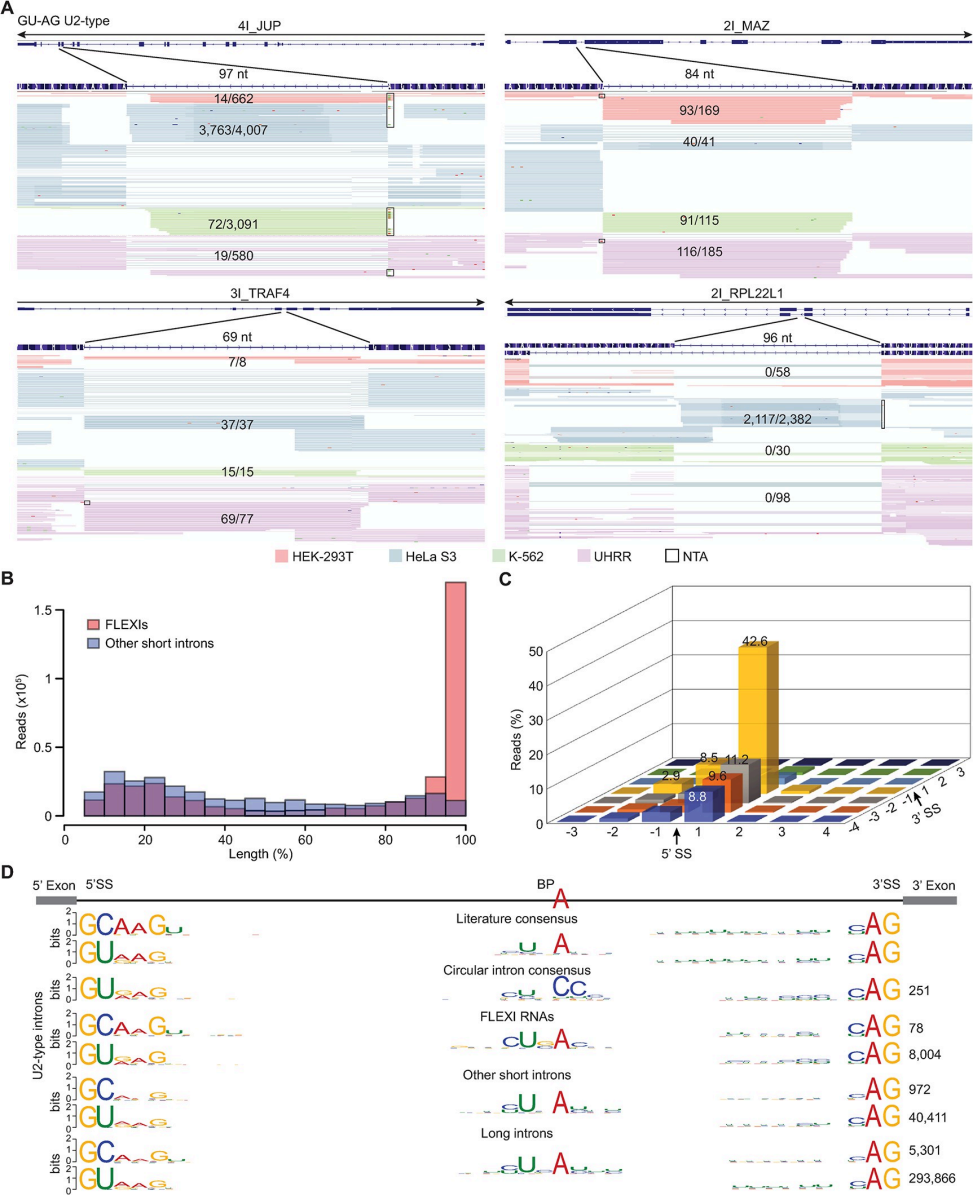
FLEXI RNAs are detected by TGIRT-seq as continuous full-length, end-to-end reads. (A) Integrative Genomics Viewer (IGV) screenshots showing read alignments for FLEXI RNAs in RNA samples from different cell lines. FLEXIs are named at the top above an arrow indicating the 5’ to 3’ orientation of the RNA with the tracks below showing gene annotations (exons, thick lines; introns, thin lines) followed by alignments of FLEXI reads in different cellular RNA samples color coded by sample type. The length of the intron that is the focus of each panel is shown above the read alignments, and the fraction of FLEXI reads (continuous reads that began and ended within 3 nt of the annotated 5’- and 3’-splice sites) for that intron in different cell lines is shown within the read alignments. Boxes indicate non-templated nucleotides added to the 3’ end of cDNAs by TGIRT-III (denoted NTA). Reads were down sampled to a maximum of 100 for display in IGVs. (B) Histogram of the length distribution of reads mapping to introns identified as FLEXIs (red) compared to those mapping to other Ensemble GRCh38-annotated short introns (≤300 nt; blue) in a merged dataset for the K-562, HEK-293T, HeLa S3, and UHRR cellular RNA samples. Percent length was calculated from TGIRT-seq read spans in the indicated intervals normalized to the length of the corresponding intron. FLEXIs encoding embedded sno/scaRNAs were excluded to avoid contributions from fully or partially processed sno/scaRNAs. (C) Three-dimensional bar graph showing the percentage of FLEXI reads in a merged dataset for the 4 cellular RNA samples ending at different positions around intron-exon junctions. Arrows indicate the 5’- and 3’-splice sites (5’SS and 3’SS, respectively). (D) Splice-site and branch-point (BP) consensus sequences of FLEXIs corresponding to GC-AG and GU-AG U2-type spliceosomal introns in a merged dataset for the 4 cellular RNA samples compared to the literature consensus sequences for human U2-type spliceosomal introns [29,30], circular introns [12], and Ensemble GRCh38-annotated U2-type other short (≤300 nt) or long (>300 nt) introns. The number of introns with each consensus sequence is indicated to the right.

Cellular FLEXI RNAs stood out in Integrated Genomics Viewer (IGV) alignments of reads mapping to their host genes as discrete blocks of continuous intron reads that began and ended within 3 nt of annotated splice sites (Fig 2A). We used this as a conservative definition of FLEXIs that would not count those that were partially degraded by cellular RNases to stable structured or protein-protected regions. The IGV alignments for 4I_JUP, an annotated agotron [19], provided an example of a such an excised intron RNA for which a high proportion (94%) of the reads satisfied the conservative definition of a FLEXI in whole-cell RNAs from HeLa S3 cells, but had 3’ truncations in the other cellular RNA samples that resulted in only small proportions (2–3%) being counted as FLEXIs (Fig 2A, top left). FLEXI reads showed no indication of reverse transcription impediments or misincorporation at or near a branch-point nucleotide as might be expected for a lariat RNA (Fig 2A).

A histogram of the length distribution of all reads that mapped to FLEXIs in the 4 cellular RNA samples showed that the predominant bin corresponded to 90–100% intron length (Fig 2B, pink bars). By contrast, the reads that mapped to other short introns (≤300 nt) annotated in GRCh38 corresponded to heterogeneously sized RNA fragments distributed across the length of the intron, as expected for rapid degradation of canonical excised intron RNAs (Fig 2B, blue bars). Most of the reads that mapped to FLEXIs began and ended directly at the splice sites (87% for the 5’-splice site and 58% for the 3’-splice site), with <10% extending a short distance into a flanking exon (Fig 2C for all FLEXIs and S3B Fig for FLEXIs in each cellular RNA sample).

Most of the FLEXIs (8,082 of 8,144) identified in the 4 cellular RNA samples and plasma had sequence characteristics of major U2-type spliceosomal introns (8,004 with GU-AG splice sites and 78 with GC-AG splice sites; 36 had sequence characteristics of minor U12-type spliceosomal introns (34 with GU-AG and 2 with AU-AC splice sites); and 26 had non-canonical splice sites (*e*.*g*., AU-AG and AU-AU; Fig 2D for U2-type FLEXIs and S4 Fig for U12 type and non-canonical splice site FLEXIs) [28,29]. The splice-site and branch-point (BP) consensus sequences of the identified FLEXIs were similar to literature consensus sequences of canonical human introns [28,30] as well as those of other short and long introns in the same TGIRT-seq datasets, with no indication of enrichment for a CC branch-point sequence characteristic of the major class of trimmed lariat introns (Figs 2D and S4) [10,12,30].

TGIRT template switching requires RNA templates with a free 3’ end, a feature lacking in trimmed intron lariat or circular introns. Consequently, none of the identified FLEXIs corresponded to a previously identified trimmed lariat intron [10,12]; was present in databases of circular RNAs generated by backsplicing (circRNADb, http://reprod.njmu.edu.cn/circrnadb; circBase, http://www.circbase.org); or was identified by CIRI2 [31] as having back-spliced circular RNA junctions in TGIRT-seq datasets of chemically fragmented UHRR [32]. RT-qPCR assays for several relatively abundant FLEXIs confirmed that they were sensitive to 5’- and 3’-exonuclease digestion, whereas a synthetic FLEXI RNA circularized *in vitro* was resistant to digestion by these nucleases (S5A and S5B Fig and S3 Table). Although hydrolytic splicing, which would directly produce an excised linear intron RNA, cannot be excluded, the largely canonical branch-point sequences in FLEXIs suggest that most if not all were excised as lariat RNAs and debranched after splicing, as found previously for mirtron pre-miRNAs and agotrons [17–19].

### Abundance of FLEXI RNAs

**Fig 3:**
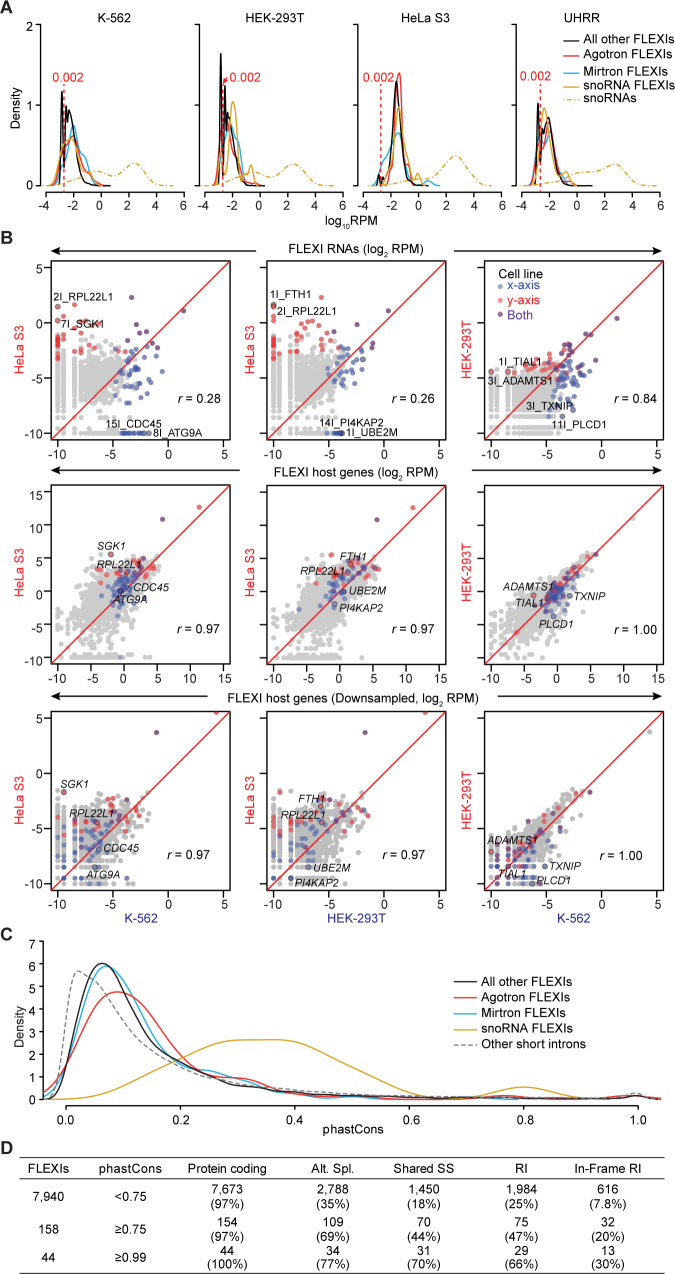
FLEXI RNA abundance, cell-type specific expression patterns, and conservation. (A) Density distribution plots of the abundance of different categories of FLEXIs in different cellular RNA samples color coded as shown at the top right. A plot for the abundance distribution of annotated snoRNAs (dashed yellow line) in each cellular RNA sample is shown for comparison. The vertical dashed red line in each plot separates lower and higher abundance FLEXIs (<0.002 and ≥0.002 RPM, respectively). (B) Scatter plots showing pairwise comparisons of FLEXI and host gene RNA abundance (log_2_-transformed RPM) in different cellular RNA samples. Top row, all reads that mapped to different FLEXIs; middle row, all reads that mapped to different FLEXI host genes; bottom row, all reads that mapped to different FLEXI host genes down sampled to match the sequencing depth of FLEXI reads. Dots for relatively abundant FLEXI RNAs (≥0.01 RPM) that were reproducibly highly expressed in one or both of the compared cell lines in all technical replicates are colored red, blue, or purple in the top row of plots with examples named in the plots and dots corresponding to their host gene RNAs similarly named and color coded in the middle and bottom rows of plots. Pearson correlation coefficients (*r*) for all detected FLEXIs or their host gene RNAs are shown for each scatter plot. (C) Density distribution plots of phastCons scores for different categories of FLEXIs in the cellular and plasma RNA samples compared to those of other annotated short introns (≤300 nt). PhastCons scores were calculated as the average score across all intron bases from multiple sequences alignment of 27 primates, including humans, mice, dogs, and armadillos. (D) Characteristics of conserved FLEXIs with different phastCons scores (<0.75, ≥0.75, and ≥0.99) in a merged dataset for the K-562, HEK-293T, HeLa S3, and UHRR cellular RNA samples. The number of FLEXIs in each group is shown to the left, and the numbers and percentages FLEXIs in different categories are shown to the right. Alt. Spl. indicates FLEXIs that were alternatively spliced to generate different protein isoforms based on Ensemble GRCh38 annotations. Shared SS indicates FLEXIs that share a 5’- or 3’-splice site with a longer intron (>300 nt). RI indicates FLEXIs that are retained in some annotated alternatively spliced transcripts, and In-Frame RI indicates subsets of those retained FLEXIs that have reading frames in frame with their flanking exons.

Density plots of the abundance of FLEXIs in the each of the 4 cellular RNA samples showed separate peaks for lower and higher abundance FLEXIs (<0.002 or ≥0.002 RPM, respectively) with a tail extending to 6.9 RPM, overlapping the lower end of the abundance distribution for snoRNAs in the same samples (dashed yellow line; Fig 3A). In HeLa S3 cells, the peak for higher abundance FLEXIs was more pronounced than in the other cellular RNA samples (Fig 3A). Relatively abundant FLEXIs included 3,363 detected at ≥0.01 RPM in one or more of the cellular RNA samples.

To estimate copy number per cell values of FLEXI RNAs, we used sncRNAs with literature-reported copy number per cell values [33] to produce a linear regression model for the relationship between log_10_-transformed RPM values in the TGIRT-seq datasets and log_10_-transformed copy number per cell values (S6A Fig and S4 Table). This method indicated that moderately abundant FLEXIs (0.01–0.99 RPM) were present at 15 to 239 copies per cell and that the most abundant FLEXIs (≥1 RPM) were present at 363 to 1,863 copies per cell. Based on the linear regression model, FLEXIs corresponding to annotated agotrons were present at up to 1,335 copies per cell; those corresponding to mirtron pre-miRNAs at up to 225 copies per cell; and those encoding annotated snoRNAs at up to 265 copies per cell. Direct measurements of the cellular abundance of several FLEXIs by droplet digital PCR (ddPCR) in the HEK-293T, HeLa S3, and UHRR samples gave variable results when normalized to different sncRNAs in different cell lines. Those normalized to sncRNAs U7, whose RPM value from TGIRT-seq was closest to the RPM values for FLEXIs, gave copy number per cell values similar to those for the linear regression model in most cases, while those for the more abundant SNORD14B and SNORD44 gave copy number per cell values for FLEXIs similar to those for the linear regression model in some cases but differing by an order of magnitude in other cases (S6B Fig, S4 Table). Collectively, these measurements established a range of copy number cell values for relatively abundant FLEXIs, with the most consistent estimates in a range of hundreds to a few thousand copies per cell based on the linear regression model (S6B Fig).

### FLEXI RNA expression patterns differ between cell lines

Scatter plots comparing the relative abundance of FLEXIs (log_2_-transformed RPM) in all technical and biological replicates of RNAs from different cell lines identified subsets of relatively abundant FLEXIs (≥0.01 RPM) that were differentially expressed in each cell line (color coded along with their host genes in Figs 3 and S8). The cell-type specific expression patterns of FLEXI RNAs were confirmed by t-SNE and ZINB-WaVE [34], which showed clustering of FLEXI expression profiles in technical and biological replicates of TGIRT-seq datasets for unfragmented UHRR and whole cell RNAs from 5 different human cell lines (HEK-293T, HeLa S3, K-562, MDA-MB-231, and MCF7) for which we had additional biological and technical replicates from previous studies (S7A Fig, S1 Table). Notably, the differential expression patterns of FLEXIs were more discriminatory (lower *r* values) between cell types than those for all RNAs whose reads mapped to the same hosts genes (higher *r* values; Fig 3B, top and middle rows, respectively), even after down sampling of host genes reads to match the lower sequencing depth of FLEXI reads (Fig 3B, bottom row; p<0.0001 by Fisher’s r-to-z transformation in both cases). The clustering of cell-type specific expression patterns appeared to improve only slightly when restricted to more abundant FLEXIs detected at ≥0.01 RPM, but not further with higher abundance cutoffs, likely reflecting that clustering was already biased toward higher abundance FLEXIs (S7B Fig).

The more discriminatory expression patterns of FLEXI RNAs, a desirable characteristic for RNA biomarkers, reflects that their abundance depends not only upon the rate of transcription of their host genes, but also on differences in alternative spicing and the rates of RNA splicing and turnover of different intron RNAs encoded by the same gene. This combination of factors can result in wide differences in the expression levels of FLEXIs from the same host gene (examples shown in IGVs in S8 Fig). Supporting the potential of FLEXIs to serve as cellular RNA biomarkers, a heatmap of the 200 FLEXIs with the highest variations in expression levels between different cell lines identified subsets of relatively abundant FLEXIs that have cell-type specific expression patterns and were detected at higher abundance in most technical and biological replicates for the same, but not other, cell lines, despite suboptimal sequencing depth in some biological replicates (heat map S9A Fig, box plots S9B Fig).

### Evolutionary conservation of FLEXIs

PhastCons scores provide a measure of evolutionary conservation, with higher phastCons scores indicating a higher probability that a sequence was conserved across a range of organisms [35]. Density plots of phastCons scores for FLEXIs calculated across 27 primates, including humans, plus mice, dogs, and armadillos, showed that most categories of FLEXIs were poorly conserved (peaks at 0.06–0.09), with agotrons (red line) having the highest phastCons scores in this range and with peaks for all categories of FLEXIs in this range extending to higher phastCons scores (Fig 3D). FLEXIs encoding snoRNAs had higher phastCons scores than other FLEXIs, likely reflecting conservation of the encoded snoRNA (Fig 3C, yellow line). Overall, FLEXIs appeared to have slightly higher phastCons score than other annotated short introns (peak at 0.02). The relatively low phastCons scores of most FLEXIs could reflect more recent acquisition and/or more rapid sequence divergence in the human lineage.

Although most FLEXIs were poorly conserved, 158 FLEXIs that did not encode a snoRNA had phastCons scores ≥0.75 (Fig 3D), suggesting an evolutionarily conserved, sequence-dependent function. Compared to FLEXIs with lower phastCons scores, these more highly conserved FLEXIs were enriched in introns that were alternatively spliced (69% compared to 35%); shared a 5’- or 3’-splice-site with a longer intron (44% compared to 18%); corresponded to an alternatively spliced retained intron (47% compared to 25%); or corresponded to an alternatively spliced retained intron with an in-frame protein-coding sequence (20% compared to 7.8%; Fig 3D). The proportion of FLEXIs with these characteristics was even higher for the 44 most highly conserved FLEXIs (phastCons scores ≥0.99; Fig 3D). For the 44 most highly conserved FLEXIs, we found no conserved motifs using the discovery algorithm MEME (https://meme-suite.org/meme/) nor differences in the presence of splicing enhancers or repressors sequences [36, 37] compared to less conserved FLEXIs. Collectively, these findings suggest that the sequence conservation of many FLEXIs with high phastCons scores reflects evolutionary constraints on different protein isoform resulting from alternative splicing, but leaves open the possibility that some FLEXIs have another conserved sequence-dependent function.

### FLEXI RNAs have binding sites for RBPs with diverse cellular functions

**Fig 4:**
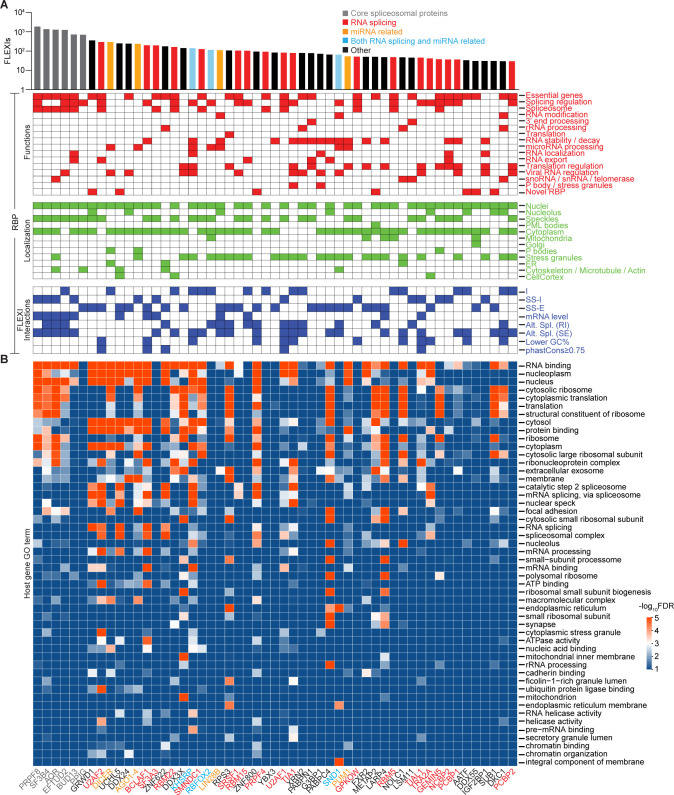
FLEXI interactions with RNA-binding proteins. (A) Bar graph showing the number of different FLEXI RNAs that have a CLIP-seq-identified binding site for each of the 53 RBPs with binding sites for ≥30 different FLEXIs in a merged dataset for the K-562, HEK-293T, HeLa S3, and UHRR cellular RNA samples. An extended version of the bar graph for all 126 RBPs is shown in S10A Fig. Bar graph RBPs are color coded by protein function as shown above the bar graph. The grids below the bar graph show reported RBP Functions (red boxes), Intracellular Localization (green boxes), and FLEXI Interactions (blue boxes). RNA-binding sites: I, within introns corresponding to FLEXIs; SS-I and SS-E, bimodally enriched across the 5’- and 3’ splice sites in unspliced pre-mRNAs with the midpoints in the intron or flanking exon (SS-I and SS-E, respectively; Figs 4A and S11). mRNA levels, RBPs whose knockdown resulted in significant difference in mRNA levels (increased or decreased, DESeq2 |LFC|≥1, adjusted p≤0.05) from host gene of FLEXIs with a binding site for that RBP compared to those genes whose transcripts lacked a binding site for the same RBPs based on ENCODE RBP-knockdown datasets (p≤0.05 calculated by Fisher’s exact test; S13 Fig). Alt. Spl., RBPs whose knockdown resulted in significant changes (p≤0.05 calculated by Kolmogorov–Smirnov test) in proximate alternative splicing (retained intron (RI) or adjacent skipped exon (SE)) for FLEXIs containing a binding site for that RBP compared to FLEXIs that lacked a binding site for the same RBP (S14 Fig). Lower GC%, percentage of RBPs whose binding sites were significantly enriched in FLEXIs having lower GC% content than other FLEXIs (density plots, S16 Fig); phastCons scores ≥0.75, RBPs whose binding sites were significantly enriched in evolutionarily conserved FLEXIs with phastCons ≥0.75 (Fig 3C). RBP functions and localization for the 51 RBPs in ENCODE eCLIP datasets were based Table S2 of ref. [41] and annotations in the RNA Granule and Mammalian Stress Granules Proteome (MSGP) databases [42, 43], and those for AGO1-4 and DICER were based on the UniProt database (https://www.uniprot.org) and references [44–48]. RBP-FLEXI Interactions were based on annotated binding sites and data in ENCODE eCLIP and knockdown datasets and PAR-CLIP datasets for AGO1-4 and DICER [38–40]. (B) Heatmap of GO terms enriched in host genes of FLEXI RNAs containing binding sites for different RBPs. GO enrichment analysis was performed with DAVID bioinformatics tools, using all FLEXI host genes as the background. The color scale shown at the right is based on -log_10_-transformed false discovery rate (FDR).

To explore possible biological functions of FLEXIs, we searched for RBPs that have a binding site within or at the splice sites of FLEXIs in ENCODE eCLIP datasets for K-562 and Hep G2 cells [38] and for AGO1-4 and DICER PAR-CLIP datasets for HEK-293 cells [39,40]. We found that more than half of the detected FLEXIs (4,505; 55%) had a CLIP-seq-identified binding site for one or more of 126 different RBPs, with 53 of these RBPs (51 in the eCLIP datasets plus DICER and AGO1-4 in the PAR-CLIP datasets) having such binding sites for ≥30 different FLEXIs (Figs 4A and S10A). Based on density plots of the mid-point of published annotated binding sites inferred from cross-links in eCLIP and PAR-CLIP datasets (S11 Fig) [38–40], we classified binding sites for each of these 53 RBPs as being enriched (*i*.*e*., having a peak or peaks) within the region corresponding to the FLEXI (I) or bimodally enriched across the 5’- and 3’- splice sites in unspliced pre-mRNAs with peaks centered in the intron or flanking exons (SS-I and SS-E, respectively; Fig 4A). Despite processing of the cross-linked samples via treatment with RNase, examination of sequence reads in the ENCODE eCLIP datasets showed that 32 of the 51 RBPs with binding sites for ≥30 different FLEXIs were enriched >2-fold compared to the control eCLIP dataset (S12 Fig and S5 Table).

The 53 RBPs with binding sites for ≥30 different FLEXIs included 6 core spliceosomal proteins (PRPF8, SF3B4, AQR, EFTUD2, BUD13, and PPIG) and other proteins that function in RNA splicing; DICER, AGO1-4, and other proteins that have miRNA-related functions; and a surprising number of proteins that function in other cellular processes, including transcriptional regulation, chromatin assembly and disassembly, cellular growth regulation, and stress responses (Fig 4A; protein functions described in S6 Table). To assess functional interactions of these RBPs with FLEXIs, we focused on 51 of these RBPs for which data were available from ENCODE shRNA and siRNA knockdown datasets (*i*.*e*., all except AGO1-4 and DICER; https://www.encodeproject.org). This analysis found that knockdown of 30 of these 51 RBPs significantly impacted proximate alternative splicing (SE, adjacent skipped exon; RI, retained intron) of FLEXIs with an annotated binding site for that RBP compared to all FLEXIs that lacked an annotated binding site for that RBP, and that knockdown of 12 of these 51 RBPs significantly impacted (decreased and/or increased) mRNAs levels of host genes of FLEXIs with an annotated binding site for that RBP compared to protein-coding genes whose transcripts lacked an annotated binding site for that RBP (summarized in RBP-FLEXI Interactions Grid in Fig 4A; data analysis S13 and S14 Figs). Notably in addition to or instead of the nucleus, all of the RBPs with binding sites for ≥30 different FLEXIs were annotated as being present in the cytoplasm, nucleolus, or cellular condensates (PML bodies, stress granules, or nuclear speckles) (Fig 4A) [41–48]. The host genes encoding FLEXIs with binding sites for the 53 RBPs that have binding sites for ≥30 FLEXIs had a wide range of enriched Gene Ontology (GO) terms for both nuclear and cytoplasmic functions (Fig 4B).

**Fig 5:**
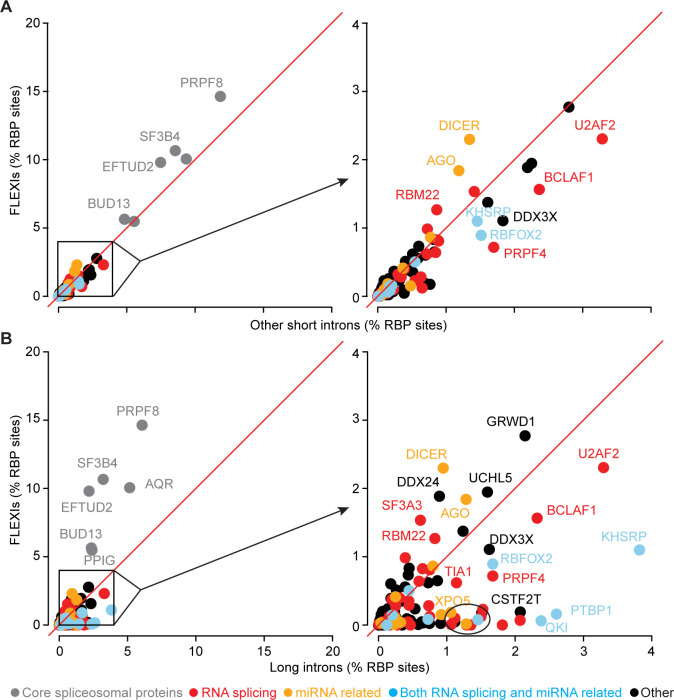
Relative abundance of annotated RBP-binding sites in FLEXIs compared to other short or long introns. (A and B) Scatter plots comparing the abundance (%) of different RBP-binding sites in FLEXIs to (A) other short introns and (B) long introns. RBPs whose binding sites constituted ≥1% of the total RBP-binding sites and whose abundance was significantly different between the compared groups (adjusted p≤0.05; calculated by Fisher’s exact test and adjusted by the Benjamini-Hochberg procedure) are indicated by the name of the protein color coded by protein function as indicated at the bottom of the Figure. For each panel, the box within the scatter plot on the left delineates a region that is expanded in the scatter plot on the right. The circled cluster of dots at the bottom left of the scatterplot on the right in panel B encompasses 12 additional RBPs (FAM120A, GTF2F1, HNRNPC, HNRNPK, HNRNPL, HNRNPM, ILF3, MATR3, NONO, PCBP2, SUGP2, and TARDBP) whose binding sites met the abundance and significance criteria for enrichment in long introns compared to FLEXIs but were too numerous to name in the Figure.

Most of the 53 proteins with a binding site for ≥30 different FLEXIs also had CLIP-seq binding sites for hundreds of other short introns and thousands of long introns (S10B and S10C Fig). Scatter plots identified 7 RBPs whose binding sites were significantly enriched for FLEXIs compared to both other short and long introns in a merged dataset for the 4 cellular RNA samples (adjusted p≤0.05; calculated by Fisher’s exact test and adjusted by the Benjamini-Hochberg procedure; Fig 5). These included 4 core spliceosomal proteins (PRPF8, SF3B4, EFTUD2, and BUD13), miRNA-related proteins AGO1-4 and DICER, and RBM22, a spliceosome component that functions in nuclear-cytoplasmic trafficking of ALU-7 and hSlu7 proteins under stress conditions [49] (Fig 5A and 5B). Compared to long introns, FLEXIs were also significantly enriched in binding sites for DDX24, a DEAD-box protein that negatively regulates RIG-I and RIG-I-like receptors by sequestering cytosolic activator RNAs [50], and 3 proteins that interact with cytoplasmically-localized, back-spliced circular exon RNAs: GRWD1, a histone-binding protein that functions in ribosome assembly and regulates chromatin dynamics (51); UCHL5 (ubiquitin carboxy-terminal hydrolase) [51]; and SF3A3 (Splicing Factor 3a subunit 3), whose interaction with a circSCAP activates TP53 [52] (Fig 5B and S6 Table). The scatter plots also identified RBPs whose binding sites were over-represented in other short or long introns compared to FLEXIs, with larger numbers and greater differences for RBPs with binding sites in long introns (Fig 5A and 5B).

### Identification of FLEXIs enriched in binding sites for non-spliceosomal RBPs

**Fig 6:**
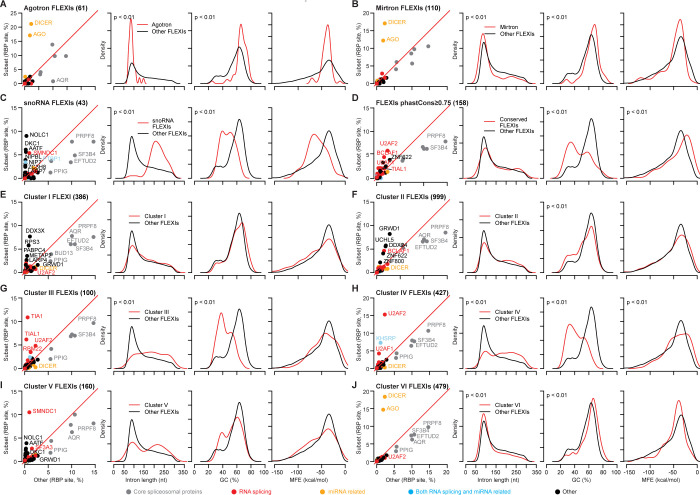
Characterization of subsets of FLEXIs enriched in binding sites for different subsets of RBPs. (A) FLEXIs annotated as agotrons; (B) FLEXIs that are pre-miRNAs of annotated mirtrons; (C) FLEXIs encoding an annotated snoRNA. Binding sites for AATF were found only in FLEXIs encoding C/D-box snoRNAs, while those for DKC1, a core H/ACA-box snoRNA-binding protein [76], were found in FLEXIs encoding both H/ACA- and C/D-box snoRNAs. (D) Conserved FLEXIs with phastCons score ≥0.75; (E-J) FLEXIs identified by hierarchical clustering as being enriched in binding sites for different subsets of RBPs (see Fig 7 below). The different subsets of FLEXIs are named above the plots with the number of FLEXIs in that subset indicated in parentheses. The leftmost panels show scatter plots for CLIP-seq-identified binding sites in the indicated subset of FLEXIs (y-axis) compared to all other FLEXIs (x-axis) in a merged dataset for the K-562, HEK-293T, HeLa S3, and UHRR cellular RNA samples, and the three panels to the right show density plots comparing different characteristics of that subset of FLEXIs to all other FLEXIs in the same merged datasets. RBP-binding site annotations in the scatter plots were based on the ENCODE eCLIP datasets for 150 RBPs [38] and AGO1-4 and DICER PAR-CLIP datasets [39, 40]. In the scatter plots, FLEXI RBP-binding sites whose abundance was ≥2% of all RBP-binding sites and significantly different between the compared subset and all other FLEXIs (adjusted p≤0.05; calculated by Fisher’s exact test and adjusted by the Benjamini-Hochberg procedure) are labeled with the name of the RBP color coded by protein function as indicated at the bottom of the Figure. The density distribution plots compare the length, GC content, and MFE for the most stable secondary structure predicted by RNAfold for the subset of FLEXIs (red) compared to all other detected FLEXIs (black). p-values for significant differences are shown at the top left of those density plots in which the distribution for the subset of FLEXIs differed significantly from other FLEXIs (p<0.01 by Kolmogorov–Smirnov test and false positive rate <5% as determined by 1,000 Monte-Carlo simulations). Similar plots for each individual RBP associated with each cluster are shown in S16 Fig.

Although FLEXIs as a whole were enriched in CLIP-seq-identified binding sites for 6 core spliceosomal proteins compared to other RBPs (Fig 5A and 5B), scatter plots readily identified subsets of FLEXIs that were significantly enriched in binding sites for other subsets of RBPs (≥2% of RBP-binding sites; adjusted p≤0.05 calculated by Fisher’s exact test and adjusted by the Benjamini-Hochberg procedure; Fig 6). These included: (i and ii) FLEXIs annotated as agotrons (n = 61) or pre-miRNAs of annotated mirtrons (n = 110), which were significantly enriched in binding sites for AGO1-4 and DICER (Fig 6A and 6B); (iii) snoRNA-encoding FLEXIs (n = 43), which were significantly enriched in binding sites for snoRNA-related proteins DKC1 (Dyskerin) and NOLC1 (Nucleolar and Coiled Body Phosphoprotein 1, alias Nopp140) [53,54]; SMNDC1 (Survival of Motor Neuron Domain Containing 1, alias SPF30); and AATF (Apoptosis Antagonizing Transcription Factor, as well as several other proteins (Fig 6C); and (iv) highly conserved, FLEXIs (phastCons scores ≥ 0.75, n = 158), which were significantly enriched in binding sites for splicing factors U2AF1 and U2AF2, the splicing regulator TIAL1, ZNF622, which functions in cytoplasmic maturation of 60S ribosomal subunits [55], and the transcription factor BCLAF1 (Fig 6D). In some cases, the FLEXIs in these 4 subsets differed significantly (p ≤ 0.01) from FLEXIs as a whole in length, GC content, and/or MFE for the most stable secondary structure predicted by RNAfold (Fig 6A–6D, right hand panels). snoRNA-encoding FLEXIs (Fig 6C) were notable for having both lower GC contents and lower MFEs than other FLEXIs, suggesting that the lower MFEs might be due to higher stability of the folded snoRNA portion or longer lengths of these FLEXIs. All 4 major proteins with binding sites in snoRNA-encoding FLEXIs (AATF, DKC1, NOLC1, and SMNDC1) were found previously to bind mature snoRNAs [54,56–58]. Although most of the binding sites for these 4 proteins (82–88%) were likewise in the snoRNA-encoding portion of the FLEXI, knockdown of all 4 proteins impacted proximate alternative splicing of snoRNA-encoding FLEXIs, suggesting that these RBPs bound initially to unspliced pre-mRNA (Figs 4A and S13). AATF was found previously to bind to both DKC1 mRNA and snoRNAs, potentially linking the ribosome biosynthesis function of snoRNAs to the regulation of cell proliferation and apoptosis by suppressing TP53 activity [56], and BCLAF1, which we found significantly enriched in conserved FLEXIs, was previously suggested to link RNA splicing to transcriptional and other cell regulatory pathways [59,60].

### Computational identification of FLEXIs co-enriched in binding sites for subsets of RBPs

**Fig 7:**
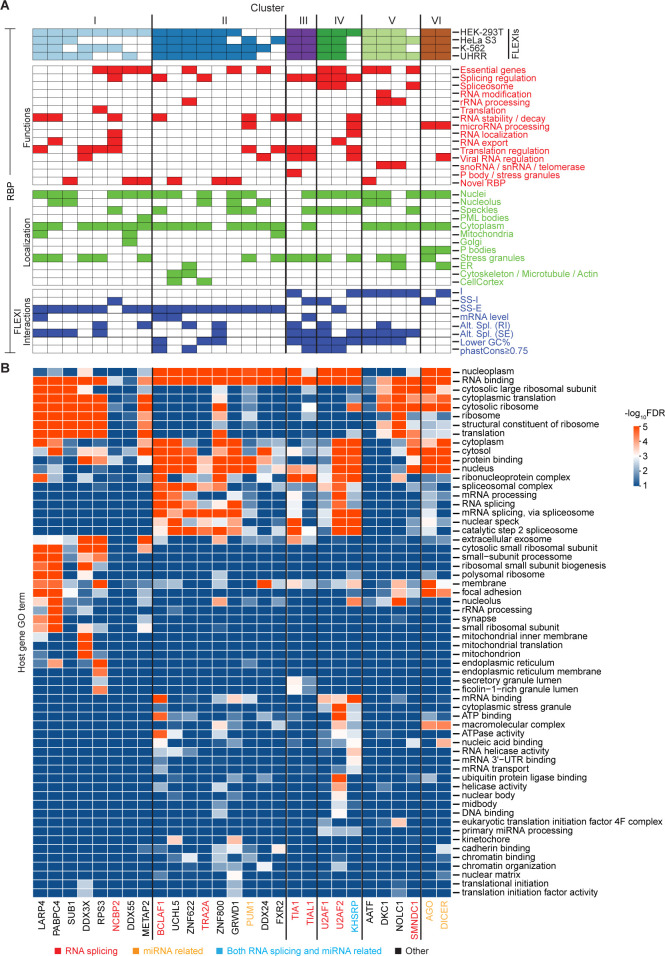
Identification of RBPs whose binding sites were co-enriched in clusters of FLEXI RNAs. (A) Hierarchical clustering of FLEXI RNAs based on patterns of over- and under-represented RBP-binding sites (see Materials and Methods and S15 Fig). Clusters I to VI are delineated at the top of panel A, and the names of the RBPs associated with each cluster are shown at the bottom of panel B color coded by protein function as indicated at the bottom of the Figure. The RBPs that met the abundance and significance criteria for association with the clusters in HEK-293T, HeLa S3, K-562, and UHRR are indicated by filled boxes in the grid at the top. The grids below summarize RBP Functions, Localization, and Interactions with FLEXIs based on data sources and analysis indicated in the legend of Fig 4. (B) Heatmap of GO terms enriched in host genes of FLEXI RNAs containing binding sites for RBPs associated with different clusters. GO enrichment analysis was performed with DAVID bioinformatics tools, using all FLEXI host genes as the background. The color scale to the right is based on -log_10_-transformed false discovery rate (FDR).

Encouraged by the above findings, we used hierarchical clustering as described in Materials and Methods to computationally identify subsets of RBPs whose binding sites were co-enriched in different subsets of FLEXIs without *a priori* assumptions about the function of the proteins or associated FLEXIs. This approach identified 6 clusters of RBPs whose binding sites were significantly co-enriched (relative abundance ≥2%, adjusted p≤0.05) in the same subsets of FLEXI in all or some of the 4 human cell lines (Figs 7A and S15). Scatter plots confirmed the enrichment of binding sites for each subset of RBPs relative to those for core spliceosomal proteins in each cluster of FLEXIs (Fig 6E–6J), and scatter plots for each individual RBP in each cluster confirmed co-enrichment of binding sites for the other RBPs in the same cluster (S16 Fig).

Clusters I and II had the largest numbers of FLEXIs and were the most revealing of novel RBP-FLEXI associations. Cluster I was comprised of 386 FLEXIs with two RBPs (LARP4 and PABPC4) that satisfied the relative abundance and significance criteria for association with the cluster in all 4 cell lines and 6 other RBPs (Transcription Regulator SUB1, DDX3X, RPS3, NCBP2, DDX55, and Methionine Aminopeptidase 2 (METAP2)) that satisfied these criteria in HEK-293T cells but not in some of the other cell lines (Fig 7A). LARP4 and PABPC4 function in regulating the stability and translation of mRNAs [61,62], and the other 6 proteins in this cluster either have mRNA synthesis or translation-related functions or could link these functions to other cellular regulatory processes (S6 Table). Analysis of ENCODE eCLIP and RBP-knockdown datasets showed that all of the proteins in Cluster I have annotated binding sites that extend across the splice sites in pre-mRNAs with knockdown of 5 of the 8 proteins impacting proximate alternative splicing of potentially interacting FLEXIs (retained intron (RI) or skipped adjacent exon (SE); Fig 7A, RBP-FLEXI Interactions Grid; based on data in S11, S13, and S14 Figs). All but one of these proteins (SUB1) were annotated as being present in the cytoplasm in addition to or instead of the nucleus, with 5 of the 8 proteins also annotated as being present in stress granules (Fig 7A, RBP Localization Grid).

Cluster II was comprised of 999 FLEXIs with 5 proteins (BCLAF1, UCHL5, ZNF622, TRA2A, ZNF800) satisfying the abundance and significance criteria for association with this cluster in all 4 cell lines, two (GRWD1 and PUM1) satisfying these criteria in 3 of the 4 cell lines, and two others (DDX24 and FXR2) satisfying these criteria in only one of the cell lines (Fig 7A). All of the proteins associated with this cluster function in regulation of transcription or RNA splicing (S6 Table) and have binding sites at FLEXI splice sites in pre-mRNAs, with knockdown of 4 of these proteins impacting proximate alternative splicing of potentially interacting FLEXIs or FLEXI host gene mRNA levels (Figs 7A, S11, S13 and S14). All but one of these proteins were annotated as being localized in the cytoplasm in addition to or instead of the nucleus, the sole exception being ZNF800, whose cellular localization was not available in ENCODE.

Clusters III and IV were comprised of 100 and 427 FLEXIs, respectively, that were enriched in binding sites for the splicing regulators TIA1 and TIAL1 (Cluster III) or splicing factors U2AF1 and U2AF2 (Cluster IV), with splicing regulator KHSRP satisfying the abundance and significance criteria for association with Cluster IV in HEK-293T cells (Fig 7A). Three of these proteins (TIAL1, U2AF1, and U2AF2) have binding sites at the splice sites in pre-mRNAs and the other two (TIA1 and KHSRP) have binding sites within the region corresponding to the FLEXI, with knockdown of 4 of these proteins impacting proximate alternative splicing of potentially interacting FLEXIs and/or FLEXI host gene mRNA levels (Fig 7A). In addition to or in one case instead of the nucleus, all 5 proteins in these clusters were annotated as being present in nuclear speckles and/or stress granules condensates [42,63], possibly reflecting post-splicing destinations.

Cluster V was comprised of 160 FLEXIs that were enriched in binding sites for the 4 proteins that were identified in Fig 6C as associated with snoRNA-encoding FLEXIs (DKC1, NOLC1, AATF, and SMNDC1), with the first 3 proteins satisfying the relative abundance and significance criteria for association with this cluster in all 4 of the cell lines and SMNDC1 satisfying these criteria in 2 of the cell lines (Fig 7A). Only 43 of the 160 FLEXIs in this cluster encoded an annotated snoRNA. All 4 of the RBPs in this cluster have binding sites within the region corresponding to the FLEXI with their knockdown affecting proximate alternative splicing of pertinent FLEXIs (Fig 7A).

Finally, Cluster VI was comprised of 479 relatively GC-rich FLEXIs that were enriched in binding sites for AGO1-4 and DICER (Fig 7A). Only 23 of the 250 FLEXIs in this cluster with a binding site for AGO1-4 corresponded to an annotated agotron [19], and only 44 of the 308 FLEXIs in this cluster with a binding site for DICER corresponded to a pre-miRNA of an annotated mirtron [64]. The remaining FLEXIs with a binding site for these proteins could be unannotated agotrons or mirtrons, could be cleaved by DICER to yield unannotated short regulatory RNAs, or could reflect binding of AGO1-4 and DICER to structured introns that were derived from or could evolve into agotrons or mirtrons, an evolutionary scenario suggested previously for mirtrons [18]. Analysis of PAR-CLIP datasets showed that DICER binding sites were localized within the region corresponding to the FLEXI, while those for AGO1-4 were at FLEXI splice sites in pre-mRNAs (Fig 7), potentially enabling regulation of host gene RNA splicing [65,66]. Although we continue to focus below on the subsets of RBPs that met the criteria for hierarchical clustering in Clusters I-VI, heat maps showed that numerous other RBPs had overlapping binding sites in FLEXIs belonging to these clusters, suggesting more complex regulatory networks to explore in subsequent studies (S17 Fig).

### Clusters of FLEXI RNAs identified by co-enrichment of RBP-binding sites originate from host genes with related biological functions

To assess if the host genes encoding FLEXIs in each of the 6 identified clusters might be functionally related, we analyzed GO terms for host genes encoding FLEXIs that contain binding sites for each RBP in each cluster using DAVID bioinformatic tools and displayed the results as a heat map (Fig 7B). The heat map showed that enriched GO terms for FLEXI host genes within each of the 6 clusters were more congruent with each other than those for host genes of the full set of 53 RBPs with binding sites for ≥30 FLEXIs in Fig 4B. The host genes for FLEXIs with binding sites for AGO1-4 and DICER (Cluster VI) were enriched in GO terms cytoplasmic translation and focal adhesion, while those for FLEXIs in Clusters II, III, and IV were enriched in GO terms RNA splicing, mRNA processing, and nuclear speckles, with some host genes for FLEXIs in Clusters I, II, IV, and VI enriched in the GO term protein binding. The host genes for FLEXIs with a binding site for the snoRNA-related proteins in Cluster V have functions related to cytosolic ribosomes and cytoplasmic translation, with the host genes of FLEXIs with a binding site for NOLC1 also involved in focal adhesion.

Notably, enriched GO terms for the host genes of FLEXIs in Cluster I included cytosolic ribosome and cytoplasmic translation, which were also enriched in host genes of the snoRNA-related FLEXIs in Cluster V (Fig 7B). Consistent with these GO terms, we found that the Cluster I and V FLEXIs were enriched in host genes encoding cytoplasmic ribosomal proteins (9.6% and 11.2% of FLEXI host genes, respectively, compared to 1.6% of all FLEXI host genes; p<1x10^-11^ by Fisher’s exact test). These findings raised the possibility that Clusters I and V RBPs function in parallel pathways for regulating the expression of host genes involved in ribosome synthesis and translation. Collectively, the GO term analysis indicated that the host genes for FLEXIs that contain binding sites for the same subset of RBPs are functionally related and might be coordinately regulated by these RBPs.

### Host genes for FLEXIs are enriched in other short and long introns with binding sites for same subsets of RBPs

**Fig 8:**
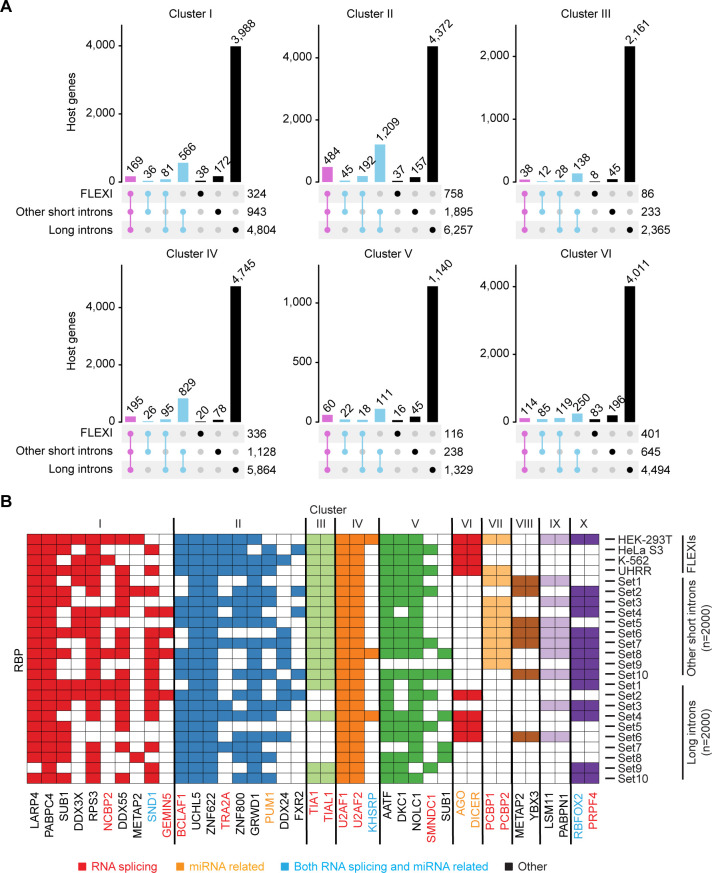
**Host genes for FLEXIs are enriched in other short and long introns with binding sies from the same subsets of RBPs,** (A) UpSet plots showing the distribution of other short (≤300 nt) and long (>300 nt) introns in host genes for FLEXIs with CLIP-seq identified binding sites for RBPs belonging to Cluster I to VI. (B) Hierarchical clustering for co-enrichment of RBP-binding sites in other short and long introns was done as in Fig 7 for 10 subsets of 2,000 randomly selected other short introns or long introns (Set1-10). Hierarchical clustering of other short and long introns with binding sites for different subsets of RBPs recapitulated Clusters I to VI found for FLEXIs with additional RBPs associated with these clusters as well as additional clusters of RBPs (VII to X) found for some subsets of other short or long introns. RBPs whose binding sites were enriched in subsets of FLEXIs in HEK-293T, HeLa S3, K-562, and UHRR are shown at the top for comparison. Names of RBPs are color coded by protein function as indicated at the bottom of the Figure.

**Fig 9:**
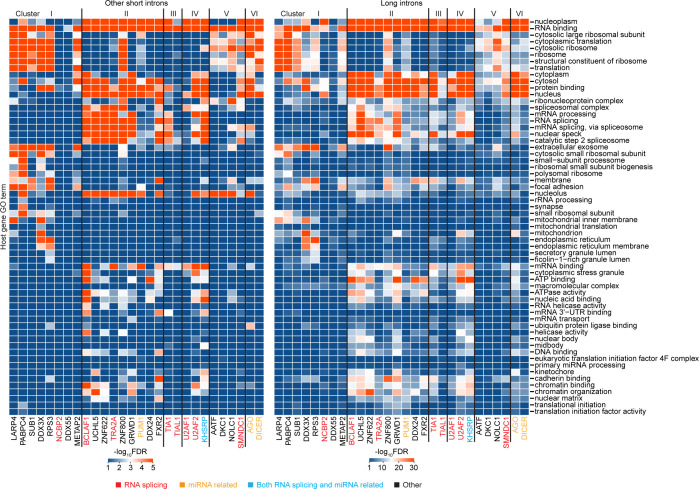
GO terms for host genes of non-FLEXI introns with binding sites for Clusters I to VI RBPs. GO term enrichment analysis was done using DAVID bioinformatics tools for host genes containing other short (left) or long (right) introns. The color scales at the bottom of each heat map were based on -log_10_-transformed FDR. RBP names are color coded by protein function as indicated at the bottom of the Figure.

All of the RBPs identified as having annotated binding sites in FLEXIs also had annotated binding sites in other short and long introns (S10B and S10C Fig). UpSet plots showed that most FLEXIs containing binding sites for the RBPs in Clusters I to VI were enriched in host genes that also contained other short or long introns with binding sites for the same RBPs, while the majority of other short introns and the vast majority of long introns were present in host genes that lacked FLEXIs with binding sites for Cluster I to VI RBPs (Fig 8A). Consistent with these findings, hierarchical clustering, done similarly to that for FLEXIs using 10 subsets of 2,000 randomly selected other short or long introns, largely recapitulated Clusters I to VI for FLEXIs with some simulations finding additional RBPs associated with these clusters, as well as additional clusters of other short and long introns co-enriched in binding sites for other RBPs (Clusters VII to X; Fig 8B). The host genes for Clusters I and V identified by hierarchical clustering of other short or long introns included those with binding sites for the same RBPs identified by hierarchical clustering for FLEXIs and were likewise enriched in GO terms for cytoplasmic translation and cytosolic ribosome (Fig 9, left and right heat maps, respectively, compare with Fig 7B). Analysis of CLIP-seq and knockdown datasets showed that in most cases the Cluster I to VI RBPs had binding sites in other short and long introns at similar locations at splice sites or within the introns as they did for FLEXIs, with knockdown of some RBPs impacting proximate alternative splicing of the associated introns or their host gene mRNA levels (S11, S13 and S14 Figs). Collectively, these findings indicate that different subsets of host genes are enriched not only in FLEXIs but also other short introns and long introns with binding sites for similar subsets of RBPs, potentially reinforcing coordinate regulation of gene expression by these RBPs.

### The intracellular localization of FLEXIs correlates with that of their CLIP-seq-identified RNA-binding proteins

Most of the RBPs associated with Clusters I and II were annotated as being present in both the cytoplasm and nucleus, with a number of these proteins having known cytoplasmic functions (Fig 7A). To investigate the intracellular location of FLEXIs with binding sites for different RBPs, we used TGIRT-seq to analyze RNAs in nuclear and cytoplasmic fractions prepared from 4 different cell lines (K-562, HeLa S3, MDA-MB-231, and MCF7). The breast cancer cell lines MDA-MB-231 and MCF7 were included in this analysis to assess the generality of conclusions based on the initially analyzed cellular RNA samples, as well as the potential utility of FLEXIs as breast cancer biomarkers. TGIRT-seq of MDA-MB-231 and MCF7 whole-cell RNAs identified 1,288 additional FLEXIs that were not detected in any of the previously analyzed cellular RNA samples (501 in MCF7, 599 in MDA-MB-231, and 188 in both MCF7 and MDA-MB-231), including cell-type specific FLEXIs from oncogenes and tumor suppressor genes (S18 Fig and S7 Table). The clean separation of nuclear and cytoplasmic fractions from the 4 cell lines was confirmed by scatter plots comparing different RNA biotypes in each fraction from each cell type (S19 Fig). Principal Component Analysis (PCA) and a sample distance heat map showed that the TGIRT-seq datasets clustered mainly by cell type, with nuclear, cytoplasmic, and whole-cell RNAs from each cell type forming separate but closely spaced subclusters (S20 Fig).

**Fig 10:**
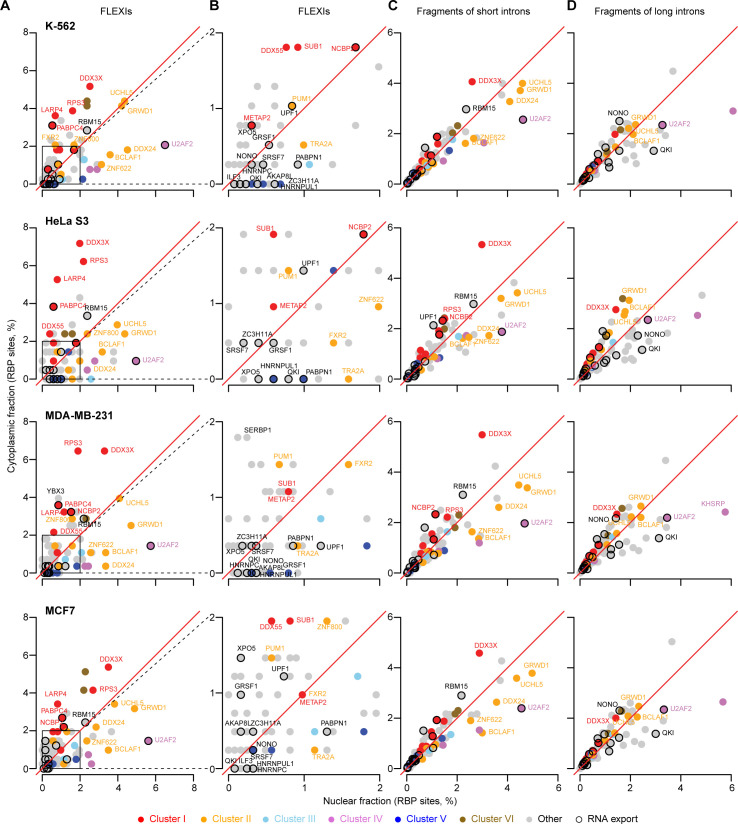
The intracellular localization of FLEXI RNAs correlates with that of their CLIP-seq-identified RNA-binding proteins. Scatter plots comparing the relative abundance of annotated RBP-binding sites in FLEXIs and fragments of other short introns and long intron RNAs in nuclear or cytoplasmic fraction from K-562, HeLa S3, MDA-MB-231, and MCF7 cells. (A and B) Scatter plots for FLEXIs. The box within the scatter plot in panel A delineates a region that is expanded in panel B. (C and D) Scatter plots for other short and long introns, respectively. Each dot in the scatter plots represents the relative abundance of RBP-binding sites in intron RNAs in the nuclear and cytoplasmic fractions, with dots for different categories of RBPs color coded as shown below the plots. In panels A and B, only FLEXIs that were differentially enriched in the nuclear or cytoplasmic fractions (Fold Change >1.5 by DESeq2 analysis; denoted nuclear and cytoplasmic FLEXIs, respectively) were subject to this analysis. For A and B, RBPs that belong to Clusters I and II or function in nuclear RNA export are indicated by named dots in the scatter plots. Other named dots are RBPs whose binding sites constituted ≥2% of all RBP-binding sites and were significantly enriched in FLEXI RNAs in the nuclear or cytoplasmic fractions (adjusted p≤0.05; calculated by Fisher’s exact test and adjusted by the Benjamini-Hochberg procedure). In panels C and D, named RBPs are those that belong to Clusters I and II, function in nuclear RNA export and whose binding sites were significantly enriched in RNA fragments of other short or long introns in the nuclear or cytoplasmic fractions (abundance ≥2%, adjusted p≤0.05; calculated by Fisher’s exact test and adjusted by the Benjamini-Hochberg procedure).

**Fig 11:**
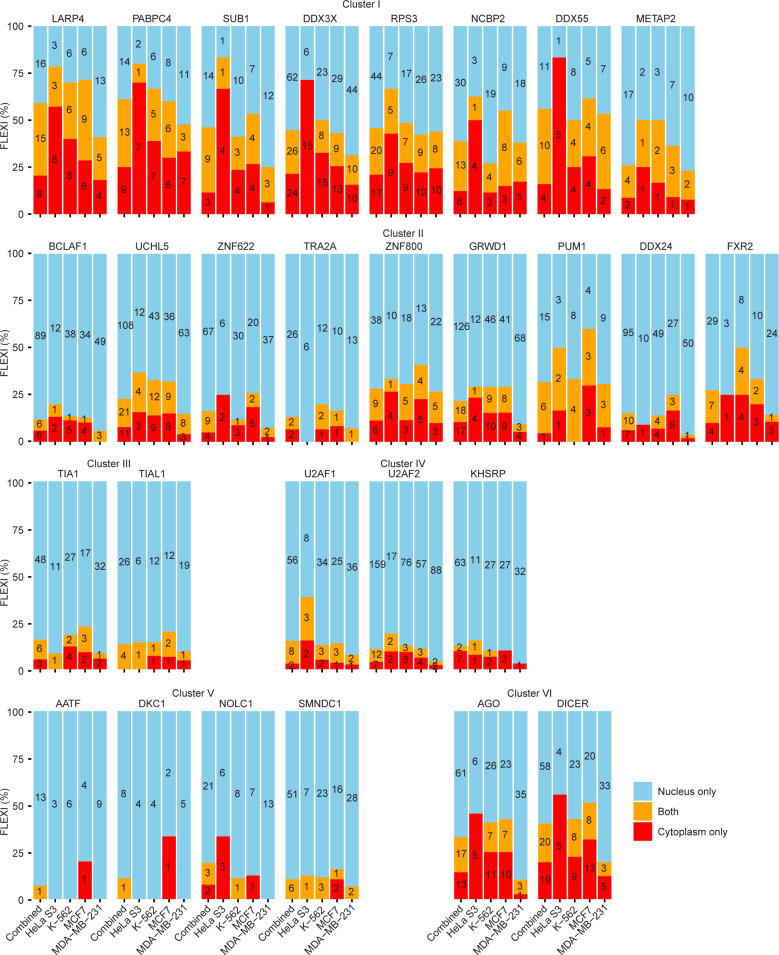
Distribution of FLEXI RNAs between nuclear, cytoplasmic, or both fractions. Stacked bar graphs show the percentages of FLEXI RNAs containing binding sites for RBPs associated with Clusters I to VI in nuclear, cytoplasmic, or both fractions color coded as shown at the bottom right in combined (left bar) or separate (right bars) TGIRT-seq datasets for 4 different cell lines. The number of FLEXIs identified in either or both fractions is indicated within each segment of the stacked bar graph.

Fig 10A and 10B show scatter plots for each of the 4 cell lines comparing the relative abundance of CLIP-seq identified RBP-binding sites in FLEXIs enriched in the nuclear or cytoplasmic fractions (fold change (FC)>1.5 via DESeq2 analysis, denoted nuclear or cytoplasmic FLEXIs, respectively). Fig 11 shows the percentages and numbers of different FLEXIs in the nuclear, cytoplasmic or both fractions with a binding site for each RBP associated with Clusters I to VI in all cell lines (Combined, left bar) or each cell line (right bars) in the order shown at the bottom of the Figure. Both Figures show that FLEXIs with binding sites for all Cluster I RBPs were enriched in the cytoplasmic fraction (name-labeled red dots in scatterplots of Fig 10 and red and orange bars in Fig 11). These included not only proteins with known cytoplasmic functions (LARP4, PABPC4, RPS3, and METAP2), but also DDX3X, which functions in nuclear RNA export [65–67], NCBP2 (nuclear cap binding protein 2), and SUB1 (a transcriptional regulator). Cytoplasmic FLEXIs in all 4 cell lines also appeared enriched with those having binding sites for AGO1-4 and DICER, consistent with the cytoplasmic functions of agotrons and mirtrons (Fig 10A and 10B, unlabeled brown dots, and Fig 11 red bars).

The FLEXIs with binding sites for Cluster II-associated proteins (name-labeled orange dots in the scatter plots) had more variable distributions. Those with binding sites for PUM1, FXR2, and ZNF800 had the highest degree of cytoplasmic enrichment in at least 3 of the 4 cell lines; those with binding sites for UCHL5 and GRWD1 were more moderately enriched in the cytoplasm; and those with binding sites for BCLAF1, ZNF622, TRA2A, and DDX24, and TRA2A had the highest degrees of enrichment in the nuclear fraction (Figs 10 and 11). FLEXIs with binding sites for RBPs associated with Clusters III, IV, and V (light blue, purple and dark blue points in scatter plots) were enriched in the nuclear fraction (Figs 10 and 11).

Unlike FLEXIs as a whole (Fig 5), the scatter plots showed that cytoplasmic FLEXIs in one or more of the cell lines were also enriched in binding sites for 9 other proteins with functions consistent with their presence in the cytoplasm [38,68] (Fig 10A and 10B and S6 Table). These include 7 proteins that function in nuclear RNA export (AKAP8L, GRSF1, RBM15, SRSF7, UPF1, XPO5, and ZC3H11A) plus SERBP1, which stabilizes inactive ribosomes and is involved in PML-body formation, and YBX3, a non-specific RNA-binding protein and homolog of YBX1, which functions in exosomal packaging of sncRNAs [26].

In contrast to FLEXIs, other short or long introns were comprised of RNA fragments. Scatter plots comparing the distribution of binding sites in RNA fragments of other short and long introns in the nuclear and cytoplasmic fractions showed at most moderate enrichment in one or the other fraction in most cases (Fig 10C and 10D). An exception was DDX3X, whose binding sites were significantly enriched in RNA fragments of other short introns in the cytoplasmic fraction from all 4 cells lines (Fig 10C). RNA fragments of long introns in the nuclear fraction were significantly enriched in binding sites for the RNA export protein QKI in all 4 cell lines, while RNA fragments with binding sites for the RNA export protein NONO were significantly enriched in the cytoplasmic fraction from 3 of the 4 cell lines (Fig 10D).

**Fig 12:**
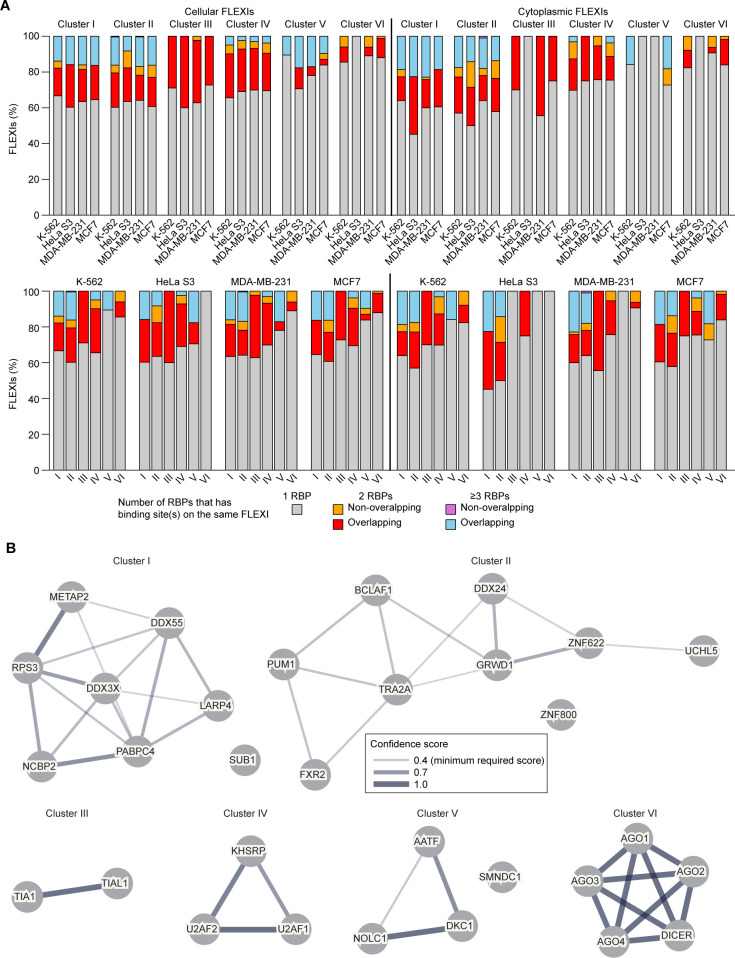
Numbers of CLIP-seq binding sites for Cluster I to VI RBPs in cellular and cytoplasmically enriched FLEXI RNAs. (A) Stacked bar graphs organized by cluster (top row) or cell type (bottom row) showing the percentage of all cellular (left) and cytoplasmically enriched (right) FLEXI RNAs that have an annotated CLIP-seq-identified binding site for a single RBP, 2 RBPs, or ≥3 RBPs associated with Clusters I to VI. Bars for FLEXI RNAs with binding sites for ≥2 RBPs are subdivided by whether or not the CLIP-seq-identified binding sites were overlapping or non-overlapping. Cytoplasmically enriched FLEXIs were defined as FLEXIs that have LFC>0 using DESeq2 in each cell line. (B) Protein-protein interactions of RBPs associated with Clusters I to VI based solely on protein-interaction network analysis using the STRING database (https://string-db.org/). Interacting RBPs are connected by lines whose thickness indicates the confidence level of the interaction score.

Stacked bar graphs based on CLIP-seq datasets showed that the majority of cellular and cytoplasmically enriched FLEXIs in Clusters I to VI have a binding site for a single RBP associated with that cluster (gray), with smaller percentages having overlapping or non-overlapping binding sites for two or more RBPs associated with the same cluster (red and blue, respectively; Fig 12A). A precedent for this situation is the alternative splicing factor RBFOX2, which binds at some splice sites via a primary conserved sequence motif and at others by binding at secondary sites or by interacting with partner proteins bound at those sites [69]. Consistent with the latter, STRING protein-network database analysis independently revealed protein-protein interactions between nearly all of the proteins in each of the 6 Clusters identified by hierarchical clustering of RNA-binding sites (Fig 12B).

## Discussion

Here, we discovered that FLEXI RNAs, previously identified as potential RNA biomarkers in human plasma [20], are members of a much larger class of cellular non-coding RNAs, with the present work identifying >9,000 different FLEXI RNAs expressed from >4,000 host genes in different human cell lines and with these numbers likely to increase further with analysis of different cellular RNA samples. This discovery was made possible by TGIRT-seq, which enables full-length, end-to-end sequencing reads of highly structured RNAs [25,32]. The identified FLEXIs were skewed toward higher GC content and more stable predicted secondary structures than those of other annotated human short introns (Fig 1C and 1D), likely contributing along with bound proteins to their higher stability in cells and plasma. We also found that FLEXIs have cell-type specific expression patterns that were more discriminatory than those of host gene mRNAs, reflecting that FLEXI abundance is dictated not only by transcription, but also by alternative splicing and differences in the rates of RNA splicing and turnover of excised intron RNAs (Figs 3 and S8). Reflecting these differences, we identified subsets of relatively abundant FLEXIs that were differentially expressed in biological and technical replicates in different cell lines (Figs 3 and S9), supporting their potential utility as cellular RNA biomarkers. We previously identified FLEXI RNAs as potential biomarkers that were differentially expressed in matched tumor and neighboring healthy breast tissues samples [70].

By searching CLIP-seq datasets, we identified 126 different RBPs that have binding sites within or at the splice sites of FLEXIs, including 53 RBPs that have such binding sites for ≥30 different FLEXIs (Figs 4A and S10. These included AGO2 and DICER, with miRNA-related functions [17,19]; snoRNA-related proteins [53,54]; and RBPs that function in transcription (AATF, BCLAF1, and SUB1), chromatin remodeling (GRWD1), translation (LARP4, METAP2, PABPC4, RPS3), ribosome maturation (ZNF622), and cellular growth regulation (DDX3X and IGF2BP1; Fig 4A and S6 Table). The wide variety of RBPs that have binding sites for FLEXIs suggests that the effect of RBP knockdowns on FLEXI host gene mRNA levels (Figs 4 and S13) could occur by multiple mechanisms, including chromatin remodeling, transcription, RNA splicing, or other post-transcriptional processes. Notably, numerous FLEXIs with binding sites for AGO1-4 and DICER did not correspond to an annotated agotron or pre-miRNAs of an annotated mirtrons, and numerous FLEXIs with binding sites for snoRNA-related proteins did not encode an annotated snoRNA (Fig 7). It will be of interest to investigate if these FLEXIs are unannotated members of these classes of sncRNAs or novel RNAs that function by base pairing to different target nucleic acids.

Analysis of annotated RBP-binding sites in eCLIP and PAR-CLIP datasets indicated that 37 of the 53 RBPs that have binding sites for ≥30 FLEXIs bind at the splice sites of unspliced pre-mRNAs with midpoints of these binding sites in the flanking exons or intron (denoted SS-E or SS-I, respectively), while the remaining 16 RBPs had binding sites within the region corresponding to the FLEXI (denoted I; Fig 4). Analysis of ENCODE datasets found that knockdown of 13 of these 16 RBPs significantly impacted proximate alternative splicing of FLEXIs with a binding site for that RBP compared to FLEXIs that lacked such a binding site, suggesting that these RBPs also bound initially to the intron in an unspliced pre-mRNA. The 3 remaining RBPs were PRPF4 and RBM5, which function in regulating RNA splicing [71,72] and DICER, which process nuclear exported pre-miRNAs in the cytoplasm [73]. Collectively, these findings indicate that most of the 53 RBPs that have binding sites for ≥30 FLEXIs bind initially to the intron in an unspliced pre-mRNA. These included 4 Cluster I RBPs with cytoplasmic functions (LARP4, PAPC4, RPS3, METAP2) that bind at splice sites in unspliced pre-mRNAs and whose knockdown impacted proximate alternative splicing of bound FLEXIs (Fig 7), making them prime candidates for confirmation and further analysis by RNA Immunoprecipitation Sequencing (RIP-seq) without RNase treatment.

Most of the 53 RBPs that have binding sites for ≥30 different FLEXIs in Fig 4A were also annotated in published datasets as being present in the cytoplasm and/or nuclear or cytoplasmic condensates in addition to or instead of the nucleus (Fig 4A). These included a number of RBPs that function in nuclear RNA export (BUD13, NCBP2, PABPC4, PABPN1, RBM15, and U2AF2) as well as AKAP8L, GRSF1, HNRNPA1, HNRNPC, HNRNPUL1, ILF3, KHDRBS1, NONO, QKI, SRSF7, UPF1, XPO5, ZC3H11A among the remaining RBPs that have binding sites in smaller numbers of FLEXIs (S10 Fig). Importantly, cell fractionation experiments confirmed cytoplasmic enrichment of subsets of FLEXIs RNAs with binding sites for RBPs that function in the cytoplasm, including AGO1-4, DDX3X, DICER, LARP4, PABCPC4, PUM1, RPS3, METAP2, and YBX3; Fig 10). FLEXIs may provide a vehicle for stabilizing these RBPs until they can bind their physiological substrates, and the bound RBPs may in turn contribute to the stability of FLEXIs as well as shield against cellular inflammatory responses to structured cytoplasmic RNAs.

By using hierarchical clustering, we identified 6 clusters of FLEXIs (Clusters I to VI) that were significantly co-enriched in binding sites for 6 subsets of RBPs, including regulatory proteins whose knockdown impacted the proximate alternative splicing of FLEXIs and/or FLEXI host gene mRNA levels (Fig 7). We then found that other short and long introns formed hierarchical clusters similar to Clusters I to VI for FLEXIs and that the host genes for FLEXIs in Clusters I to VI were enriched in other short and long introns with binding sites for the same subsets of RBPs (Fig 8). Collectively, these findings suggest that different subsets of functionally related host genes are coordinately regulated by different subsets of RBPs whose binding sites are enriched in different classes of introns within these gene sets. Particularly suggestive was Cluster I, which included two proteins, LARP4 and PABPC4, that function in regulating the stability and translation of mRNAs [62], along with additional proteins that have translation-related functions (NCBP2, METAP2, and RPS3), or that could link these functions to other cell regulatory pathways (SUB1, RPS3, DDX3X, and DDX55).

As noted previously in Results, the host genes encoding the FLEXIs in Cluster I were enriched in GO terms similar to those for the snoRNA-related FLEXIs in Cluster V (Fig 7B), suggesting that the FLEXIs and RBPs of Cluster I may function in parallel with snoRNAs in regulating ribosome synthesis (Fig 7B). Cluster II included 3 proteins (UCHL5, ZNF622, and TRA2A) that were distinguished from other proteins associated with the FLEXI-RBP clusters as being localized to the endoplasmic reticulum, cytoskeleton, and cell cortex, possibly reflecting a cytoskeleton or membrane-associated function. The RBPs associated with Clusters III and IV, which function in alternative splicing, were found localized not only in the nucleus but also in stress granules and nuclear speckles, the former possibly a mechanism for muting inflammatory responses to naked cytoplasmic RNAs, and the latter possibly a mechanism for storing alternative splicing factors for subsequent use [74]. The specificity of the CLIP-seq-identified binding sites that formed the basis for our analysis is supported by the findings that host genes for FLEXIs with binding sites for RBPs in Clusters I to VI were co-enriched with other short and long introns with binding sites for the same RBPs (Fig 8B); that knockdown of subsets of RBPs in each cluster impacted proximate alternative splicing and/or host gene mRNA levels of FLEXIs in the clusters; and that most of the RBPs in each cluster were confirmed to interact with each other by protein-interaction network analysis independently of their interactions with RNA (Fig 12B).

Lewis *et al*. [75] recently reported that 5 of the 8 RBPs that we identified as belonging to Cluster I (LARP4, PABPC4, DDX3X, RPS3, and SUB1) have eCLIP-identified binding sites enriched in mRNAs of nuclear genes encoding mitochondrially localized proteins. They also found that LARP4-binding sites were enriched near the 3’ end of mRNAs encoding components of respiratory chain complexes and mitochondrial ribosomal proteins [75]. Prompted by these findings, we found that FLEXIs with binding sites for LARP4 and other Cluster I RBPs were also enriched in host genes encoding mitochondrially localized proteins annotated in MitoCarta3.0 (8.3 to 25.5% of FLEXIs host genes compared to 7.5% of all FLEXI host genes and 5.7% of all GRCh38 annotated protein-coding genes; S21A and S21B Fig). We also found that FLEXIs with binding sites for LARP4 and other Cluster I RBPs were enriched in host genes encoding cytoplasmic ribosomal proteins (8.3 to 42.6%), consistent with enrichment for the GO terms cytoplasmic ribosome and cytoplasmic translation for FLEXI host genes in this cluster (Fig 7B). Additionally, some but not all of the FLEXIs with binding sites for Cluster I RBPs were enriched in genes encoding mitochondrial ribosomal proteins (DDX3X, 6.8%; LARP4, PABPC4, RPS3, 1.9–2.2% compared to 0% for the other 4 RBPs), with similar trends holding for other short and long introns with binding sites for the same RBPs (S21A Fig), suggesting overlapping regulatory mechanisms. Consistent with the findings of Lewis *et al*. [75], we found that binding sites for LARP4 and Cluster I proteins PABPC4, DDX55 and SUB1 were localized near the 3’ end of mRNAs (defined as UTRs + coding sequences (CDS)). Extending these findings, we found that binding sites for these 4 proteins were also localized to 3’-proximal introns, suggesting that they bind initially to unspliced pre-mRNAs and that their localization, possibly as a complex (Fig 12B), might be dictated by proximity to 3’-poly(A) tails (S21C Fig). METAP2- and RPS3-binding sites were more uniformly distributed across the length of the mRNAs, but remained concentrated in 3’-proximal introns, while DDX3X- and NCBP2-binding sites were localized near the 5’ end of mRNAs and in 5’-proximal introns, with DDX3X still showing a smaller peak for 3’-proximal introns (S21C Fig). Collectively, these findings suggest that the RBPs that have binding sites in FLEXIs in Cluster I function in at least two overlapping regulatory pathways, one for cytoplasmic translation and cytosolic ribosome biogenesis parallel to that for snoRNAs, and the other for mitochondrial function, both potentially related to regulation of cell growth and proliferation.

Overall, our findings suggest a model in which newly synthesized or excess RBPs that are not associated with their primary physiological substrates bind FLEXIs as well as other short or long introns in pre-mRNAs from subsets of genes with related functions, contributing to their coordinate regulation. After splicing, some of the RBPs may remain stably bound to FLEXIs and co-localize with them to the cytoplasm or other intracellular locations, where they may dissociate from FLEXIs to perform different cellular functions. FLEXI RNAs may serve simply as transit vehicles that stabilize RNA-binding proteins until they reach a desired intracellular location or could be actively engaged in cellular functions. Although only 6 subsets of RBPs met the arbitrary significance criteria for co-enrichment in the same subsets of FLEXIs, a heat map of overlapping RBP-binding sites identified multiple additional RBPs whose binding sites overlapped those of RBPs within each cluster of FLEXIs (S17 Fig), potentially enabling cross talk between different gene sets. Our findings provide a comprehensive road map for the further studies of the biological functions of FLEXIs and the involvement of different subsets of RBPs in coordinately regulating the expression of functionally related genes.

## Materials and methods

### Ethics statement

In this study, we obtained and analyzed TGIRT-seq datasets for RNA isolated from different human cell lines (see Methods) and re-analyzed TGIRT-seq datasets obtained in a previous study [20] for RNA isolated from commercial human plasma prepared by apheresis from multiple unidentified healthy individuals (dataset accession number PRJNA640428). This study did not use human subjects.

### RNA samples and cultured cells

RNA samples used in this work are summarized in S1 Table. Universal Human Reference RNA (UHRR) was purchased from Agilent under the product name Quantitative PCR Human Reference Total RNA. HeLa S3 and MCF7 RNAs used for initial FLEXI analysis (Figs 1–9) were purchased from Thermo Fisher. All other cellular RNAs, including all those in cell fractionation experiments (Fig 10), were isolated from cultured cells by using a mirVana miRNA Isolation Kit (Thermo Fisher).

For RNA isolation, K-562 cells (ATCC CTL-243) were propagated from an ATCC source vial and maintained in Iscove’s Modified Dulbecco’s Medium (IMDM; Thermo Fisher 12440–053) supplemented with 10% Fetal Bovine Serum (FBS; Gemini Bioscience). HEK-293T/17 cells (ATCC CRL-11268) were propagated from an ATCC source vial and maintained in Dulbecco’s Modified Eagle Medium (DMEM; Thermo Fisher 11965–065) supplemented with 10% FBS. HeLa S3 cells (ATCC CCL-2.2) were propagated from an ATCC source vial and maintained in F-12K (Thermo Fisher 21127–022) supplemented with 10% FBS. MDA-MB-231 cells (ATCC HTB-26) and MCF7 cells (ATCC HTB-22) were obtained from Dr. Blerta Xhelmaçe (University of Texas at Austin) and maintained in DMEM (Thermo Fisher 11965–092) supplemented with 10% FBS. All cultures were grown in an Eppendorf CellXpert C170i incubator at 37°C with 5% CO_2_ atmosphere. Cells (2 to 4 x10^6^) were harvested by centrifugation (300 x g for 10 min at 4°C in an Eppendorf FA-24x2-PTFE rotor) without (K-562 cells) or after trypsinization (all other cell lines) with 0.25% Trypsin-EDTA (Thermo Fisher 25200–056). The cells were then washed twice by centrifugation as above with cold Dulbecco’s Phosphate Buffered Saline (D-PBS; Thermo Fisher 14190–144). The final cell pellets were resuspended in 600 μL of mirVana Lysis Buffer, and RNA was isolated according to the kit manufacturer’s protocol with elution in a final volume of 100 μL.

### DNA and RNA oligonucleotides

DNA and RNA oligonucleotides were purchased from Integrated DNA Technologies (IDT). Those used for TGIRT-seq are listed in S8 Table. R2R DNA oligonucleotides with 3’ A, C, G, and T residues were hand-mixed in equimolar amounts prior to annealing to the R2 RNA oligonucleotide to generate RNA/DNA heteroduplexes with a 1-nt 3’-DNA overhang for RNA-seq adapter addition by TGIRT template switching.

Oligonucleotides used as reverse transcriptase or PCR primers for exonuclease and ddPCR experiments are listed in S9 Table. The sequence specificity of all primer sets was confirmed by gel purifying a representative subset of qPCR amplicons and Sanger sequencing them either directly or after TOPO-TA cloning (Thermo Fisher; S10 Table). A circular form of the 3I_RAN FLEXI synthetic oligonucleotide control was generated by incubating the 5’ phosphorylated-3I_RAN FLEXI oligonucleotide (500 ng) with T4 RNA Ligase I (10 U; New England Biolabs) for 2 h at 25°C and 2 min at 95°C. The circularized oligonucleotide was cleaned up with an RNA Clean & Concentrator kit (Zymo Research), using 8 sample volumes of ethanol added during purification (denoted 8X ethanol method) to maximize the recovery of small RNAs together with the longer RNAs.

### Exonuclease assay

Unfragmented UHRR or HEK-293T cell RNAs (5 μg) were treated with 2 U TURBO DNase (Invitrogen) and purified using an RNA Clean & Concentrator kit (Zymo Research) following the manufacturer’s small-size selection method to separate RNAs ≤200 nt. The inputs of small size-selected RNAs were scaled for optimal FLEXI detection in each sample (HEK-293T, 100 ng; UHRR, 70 ng). The size-selected RNAs or synthetic versions of FLEXIs were subjected to exonuclease treatment (1 U Terminator exonuclease (Lucigen), 20 U RNase R (Abcam), 20 U murine RNase Inhibitor (New England Biolabs) in 1X RNase R reaction buffer (Abcam), supplemented with MgCl_2_ to a final concentration of 1 mM MgCl_2_ or mock-treated under the same reaction conditions without the enzymes for 1 h at 42°C, with 3 replicates for each RNA type. The exonuclease reactions were cleaned up with the RNA Clean & Concentrator kit (8X ethanol method; Zymo Research). An aliquot of the material for synthesized FLEXIs was analyzed on a Novex 10% Tris-Borate-EDTA-urea polyacrylamide gel (Thermo Fisher) to separate linear and circular RNAs and imaged with SYBR Gold (Thermo Fisher). Reverse transcription was performed by using a gene specific primer (1 μM final concentration) located near the 3’ end of the FLEXI intron with 100 U Maxima H reverse transcriptase (ThermoFisher) in the manufacturer’s buffer at 25°C for 10 min, 50°C for 30 min, 65°C for 30 min and 85°C for 5 min. qPCR reactions run in triplicate were carried out with 1 μL cDNA, 0.3 μM primers for all primer sets, except 4I_JUP which used 0.8 μM primers, and PowerUp SYBR Green master mix (Life Technologies) using a ViiA 7 Real Time PCR system (Thermo Fisher). Matched control samples omitting the reverse transcriptase (RT-) were performed for every reaction. Mean delta cycle threshold (CT) values were calculated to obtain a fold change of the RNA levels in the treated compared to the mock-treated samples and converted into a percentage of RNA remaining after exonuclease treatment. In two cases, the exonuclease treatment led to no measurable CT value and in these cases a pseudocount CT value of 41 was added to perform the calculation. Primer sequences used in these experiments are listed in S9 Table.

### Droplet digital PCR

Unfragmented HeLa S3, UHRR or HEK-293T RNAs (5 μg) were treated with 2 U TURBO DNase (Invitrogen) and purified using an RNA Clean & Concentrator kit (Zymo Research), using the manufacturer’s small-size selection method to separate RNAs ≤200 nt. The inputs of the size-selected RNAs for reverse transcription reactions were scaled to have a similar amplification to a SNORD14B control when compared with the original total RNA inputs (HEK-293T and HeLa S3; 5 ng; UHRR, 1 ng). The RNAs were reverse transcribed by using 1 μM final concentration of a gene specific primer (S9 Table) located at the 3’ end of the FLEXI RNA with 100 U Maxima H Reverse Transcriptase (Thermo Fisher) in the manufacturer’s buffer and incubating at 25°C for 10 min, 50°C for 30 min, 65°C for 30 min and 85°C for 5 min. Matched control samples containing the small size-selected RNA, but omitting the RT (RT-) were performed for every reaction. Droplet digital PCR (ddPCR) was performed on 1 μL cDNA with 0.2 μM primers for all primer sets, except 4I_JUP which used 0.5 μM primers, in QX200 ddPCR EvaGreen Supermix (Biorad) using the QX200 Droplet Digital PCR system (BioRad). Primer sets (S9 Table) were designed to encompass either the FLEXI sequence or to span the upstream 5’ exon-FLEXI junction in order to quantify the FLEXI RNA compared to any precursor or mature mRNA with a retained FLEXI. The same gene-specific cDNA was used as the PCR template for quantifying both the FLEXI and 5’ exon-FLEXI junctions. Due to the short length of the FLEXI sequences, primer design was constrained to the best available primer location that maximized length and sequence specificity. As a result of the primer constraints, some primers amplified a smaller region of the FLEXI sequence than the full-length intron, possibly contributing to differences between ddPCR and TGIRT-seq quantification. 5’ exon-FLEXI junction primer pairs for quantitation of unspliced pre-mRNA used a reverse primer that was inset closer to the 5’ exon-FLEXI junction than that used for FLEXI quantitation, so that the size of the amplicon would be more similar to the size of the compared FLEXI amplicon. As a consequence of these constraints, some primer pairs amplified background primer dimer artifacts in addition to verified products. For each reaction replicate, any RT- signal was subtracted, and three background-subtracted reaction replicates were averaged for each primer pair. The majority of the 5’ exon-FLEXI junction reactions either gave no product or amplified non-specific products. However, a few 5’ exon-FLEXI junction reactions produced validated products sequenced by direct Sanger sequencing and/or TOPO-TA cloning and Sanger sequencing of gel extracted, matched qPCR amplicons (HEK-293T, *RPS2*; HeLa S3, *ACTB*; UHRR, *POLG* and *ACTB*). These sequence-validated 5’ exon-FLEXI junction ddPCR signals were subtracted from the FLEXI ddPCR signals to obtain the final ddPCR estimate for abundance of the FLEXI. FLEXI copies per reaction were then normalized to sncRNA standards performed in parallel: U7 (chosen for similar TGIRT-seq abundance to FLEXIs), SNORD14B [77], and SNORD44 [78]. Finally, the normalized FLEXI copies per reaction were converted to copy number per cell estimates by multiplying control values generated by the linear regression model.

### Cell fractionation

Cell cultures were grown to ~80% confluency (HeLa S3, MDA-MB-231 and MCF7) or to a density of 9 x 10^5^ cells/mL (K-562) in T-75 flasks. To prepare cell pellets, adherent cells were either washed 3X with cold D-PBS and then scraped in D-PBS and pelleted by centrifugation at 1,000 x g for 5 min at 4°C in an Eppendorf FA-24x2-PTFE rotor (HeLa S3 cells) or trypsinized (MDA-MB-2231 and MCF7 cells) and collected by centrifugation at 1,000 RPM for 5 min at room temperature in a Centra CL2 centrifuge with Aerocarrier rotor (Thermo Fisher). The cells were then resuspended in cold D-PBS and washed 3X by centrifugation at 1,000 x g for 5 min at 4°C in an Eppendorf FA-24x2-PTFE rotor. Suspension cells (K-562) were collected by centrifugation at 1,000 RPM for 5 min at room temperature in a Centra CL2 centrifuge with Aerocarrier rotor and then placed on ice until resuspended in cold D-PBS and then washed 3X by centrifugation at 1,000 x g for 5 min 4°C in an Eppendorf FA-24x2-PTFE rotor.

Cellular fractionation was performed as described by Rio *et al*. [79]. Cell pellets were resuspended in 1.0 mL of cell disruption buffer and then incubated for 10 min on ice and monitored for swelling by light microscopy. Swollen cells were transferred to a baked (400°C, 4 h) 2.0-mL glass Dounce homogenizer and broken with 10 strokes of a Type B pestle. Cell breakage was confirmed by microscopy. Cell homogenates were transferred to Eppendorf tubes and brought to 0.1% Triton X-100 by the addition of 10% (w/v) Triton X-100 (Sigma). Samples were then centrifuged immediately at 1,500 x g for 5 min at 4°C in an Eppendorf FA-24x2-PTFE rotor.

Nuclear fraction pellets and cytoplasmic fraction supernatants were transferred to fresh tubes and placed on ice. RNA was extracted by using a mirVana RNA extraction kit following the manufacturer’s instructions. The nuclear fraction pellets were resuspended in 600 μL of kit lysis buffer and processed on a mirVana column. The cytoplasmic samples were split such that 200 μL of cytoplasm was solubilized in 400 μL of kit lysis buffer and then combined and processed together on the same column. RNA samples were evaluated using an Agilent 2100 Bioanalyzer with a Eukaryotic Total RNA Pico kit using the pre-programmed mRNA assay and processed for TGIRT-seq library construction as described below.

### Thermostable Group II Intron Reverse Transcriptase sequencing (TGIRT-seq)

TGIRT-seq libraries were prepared from unfragmented cellular RNAs as described [21,22]. To remove residual DNA, UHRR and HeLa S3 cell RNAs (1 μg) were incubated with 20 U exonuclease I (Lucigen) and 2 U Baseline-ZERO DNase (Lucigen) in Baseline-ZERO DNase Buffer for 30 min at 37°C. K-562, MCF7, MDA-MB-231 and HEK-293T cell RNAs (5 μg) were incubated with 2 U TURBO DNase for 30 min at 37°C (Thermo Fisher). After DNase digestion, RNAs were cleaned up with an RNA Clean & Concentrator kit (8X ethanol method; Zymo Research). The eluted RNAs were then rRNA-depleted by using the rRNA removal section of a TruSeq Stranded Total RNA Library Prep Human/Mouse/Rat kit (Illumina), with the supernatant from the magnetic-bead separation cleaned up by using a Zymo RNA Clean & Concentrator kit with 8X ethanol. After checking RNA concentration and length by using an Agilent 2100 Bioanalyzer with a 6000 RNA Pico chip, RNAs were aliquoted into ~20 ng portions and stored at -80°C until use.

TGIRT-seq libraries were prepared as described [21, 22] using 20–50 ng of rRNA-depleted unfragmented cellular RNAs. Template-switching and reverse transcription reactions were done with 1 μM TGIRT-III (InGex, currently available upon request from the Lambowitz laboratory) and 100 nM pre-annealed R2 RNA/R2R DNA starter duplex in 20 μL of reaction medium containing 450 mM NaCl, 5 mM MgCl_2_, 20 mM Tris-HCl, pH 7.5 and 5 mM DTT. Reactions were set up with all components except dNTPs, pre-incubated for 30 min at room temperature, a step that increases the efficiency of RNA-seq adapter addition by TGIRT template switching and initiated by adding dNTPs (final concentrations 1 mM each of dATP, dCTP, dGTP, and dTTP). The reactions were incubated for 15 min at 60°C and then terminated by adding 1 μL 5 M NaOH and heating at 95°C for 5 min to degrade RNA followed by neutralization with 1 μL 5 M HCl and one round of MinElute column clean-up (Qiagen). The R1R DNA adapter was adenylated by using a 5’ DNA Adenylation kit (New England Biolabs) and then ligated to the 3’ end of the cDNA by using Thermostable 5’ App DNA/RNA Ligase (New England Biolabs) for 2 h at 65°C. The ligation products were purified by using a MinElute Reaction Cleanup Kit and amplified by PCR with Phusion High-Fidelity DNA polymerase (Thermo Fisher; denaturation at 98°C for 5 s followed by 12 cycles of 98°C for 5 s, 60°C for 10 s, and 72°C for 15 s and held at 4°C until processed further. The PCR products were cleaned up by using Agencourt AMPure XP beads (1.4X volume; Beckman Coulter) and sequenced on an Illumina NextSeq 500 to obtain 2 x 75 nt paired-end reads or on an Illumina NovaSeq 6000 to obtain 2 x 150 nt paired-end reads at the Genome Sequence and Analysis Facility of the University of Texas at Austin.

TGIRT-seq datasets for human plasma RNAs were obtained in a previous study using RNA isolated from commercial human plasma prepared by apheresis from multiple unidentified healthy individuals (20) and reanalyzed bioinformatically in this study.

### Sequencing data processing

Illumina TruSeq adapters and PCR primer sequences were trimmed from the reads with Cutadapt v2.8 (https://github.com/marcelm/cutadapt, sequencing quality score cut-off at 20; p-value <0.01) and reads <15 nt after trimming were discarded. To minimize mismapping, we used a sequential mapping strategy. First, reads were mapped to the human mitochondrial genome (Ensembl GRCh38 Release 93) and the *Escherichia coli* genome (GeneBank: NC_000913) using HISAT2 v2.1.0 (http://daehwankimlab.github.io/hisat2/) with customized settings (-k 10—rfg 1,3—rdg 1,3—mp 4,2—no-mixed—no-discordant—no-spliced-alignment) to collect reads derived from mitochondrial and *E*. *coli* RNAs (Pass 1). Unmapped read from Pass1 were then mapped to a collection of customized reference sequences for human sncRNAs and rRNAs (2.2-kb 5S rRNA repeats from the 5S rRNA cluster on chromosome 1 (1q42, GeneBank: X12811) and 43-kb 45S rRNA containing 5.8S, 18S and 28S rRNAs from clusters on chromosomes 13,14,15, 21, and 22 (GeneBank: U13369), using HISAT2 with the following settings -k 20—rdg 1,3—rfg 1,3—mp 2,1—no-mixed—no-discordant—no-spliced-alignment—norc (Pass 2). Unmapped reads from Pass 2 were then mapped to the human genome reference sequence (Ensembl GRCh38 Release 93) using HISAT2 with settings optimized for non-spliced mapping (-k 10—rdg 1,3—rfg 1,3—mp 4,2—no-mixed—no-discordant—no-spliced-alignment) (Pass 3) followed by splice aware mapping (-k 10—rdg 1,3—rfg 1,3—mp 4,2—no-mixed—no-discordant—dta) (Pass 4). Finally, the remaining unmapped reads were mapped to Ensembl GRCh38 Release 93 by Bowtie 2 v2.2.5 (https://github.com/BenLangmead/bowtie2) using local alignment (-k 10—rdg 1,3—rfg 1,3—mp 4—ma 1—no-mixed—no-discordant—very-sensitive-local) to improve mapping rates for reads containing post-transcriptionally added 5’ or 3’ nucleotides, short untrimmed adapter sequences, and non-templated nucleotides added to the 3’ end of the cDNAs by TGIRT-III during TGIRT-seq library preparation (Pass 5). For reads that mapped to multiple genomic loci with the same mapping score in Passes 3 to 5, the alignment with the shortest distance between the two paired ends (*i*.*e*., the shortest read span) was selected. In the case of ties (*i*.*e*., reads with the same mapping score and read span), reads mapping to a chromosome were selected over reads mapping to scaffold sequences, and in other cases, the read was assigned randomly to one of the tied choices. The filtered multiply mapped reads were then combined with the uniquely mapped reads from Passes 3–5 by using SAMtools v1.10 (https://github.com/samtools/samtools).

Read counts for human mitochondrial tRNAs and rRNAs were obtained from Pass 1 by intersecting mapped reads with Mitochondrial gene annotations from Ensembl GRCh38 Release 93. Read counts for sncRNAs (miRNA, tRNA, Y RNA, Vault RNA, 7SL RNA, 7SK RNA) and rRNAs were obtained directly from mapped reads from Pass 2. To obtain read counts for sncRNAs that were not included in Passes 1 and 2, the mapped reads (from Passes 3 to 5) were intersected with sncRNA annotations from Ensembl GRCh38 Release 93 gene annotations supplemented with the *RNY5* gene and its 10 pseudogenes, which were not annotated in this release. After removal of these reads, the remaining reads were intersected with the annotations for protein-coding gene RNAs, lincRNAs, antisense RNAs, and other lncRNAs to obtain read counts for these features. All of these read counts were then combined to generate a complete table for all detected human gene and features in the dataset. Coverage plots and read alignments were created by using Integrative Genomics Viewer v2.6.2 (IGV, https://igv.org). Where indicated in Figure Legends, genes with >100 mapped reads were down sampled to 100 mapped reads in IGV for visualization. Where indicated, down sampling of reads mapping to all protein coding genes was done by randomly picking mapped reads to match the read depth of FLEXI RNA reads using SAMtools.

### Identification and characterization of FLEXIs

To identify short introns that could give rise to FLEXI RNAs, intron annotations were extracted from Ensemble GRCh38 Release 93 gene annotations using a customized script and filtered to remove introns >300 nt as well as duplicate intron annotations from different mRNA isoforms. Mapped reads were then intersected with short intron annotations using BEDTools, and read pairs (Read 1 and Read 2) ending at or within 3 nucleotides of annotated 5’- and 3’-splice sites, typically columns of stacked reads with discrete boundaries in IGV alignments (Figs 2A and S4 and S8 Figs), were identified as putatively corresponding to FLEXI RNAs. Exon-junction reads were defined as those in which one of the paired-end reads mapped within the intron and the other within a flanking exon.

UpSet plots of FLEXI RNAs from different sample types were plotted by using the ComplexHeatmap package v2.2.0 in R (https://jokergoo.github.io/ComplexHeatmap-reference/book/). For UpSet plots of FLEXI host genes, FLEXI RNAs were aggregated by Ensemble ID, and different FLEXI RNAs from the same gene were combined into one entry. Density distribution plots and scatter plots were plotted by using R.

FLEXI RNAs corresponding to annotated mirtrons or agotrons were identified by intersecting FLEXI RNA coordinates with those of annotated mirtrons [64] and agotrons [19]. FLEXI RNAs containing embedded snoRNAs were identified by intersecting the FLEXI RNA coordinates with those of annotated snoRNAs and scaRNAs from Ensembl GRCh38 annotations.

5’- and 3’-splice sites (SS) and branch-point (BP) consensus sequences of human U2- and U12-type spliceosomal introns were obtained from previous publications [29,30]. FLEXI RNAs corresponding to U12-type introns were identified by searching for: (i) FLEXI RNAs with AU-AC ends and (ii) the 5’-splice site consensus sequence of U12-type introns with GU-AG ends [29] using FIMO (https://meme-suite.org/meme/tools/fimo) with the following settings: FIMO—text—norc <GU_AG_U12_5SS motif file> <sequence file>. Splice-site consensus sequences of FLEXI RNAs were determined from nucleotide frequencies at the 5’ and 3’ ends of the introns. The U2-type intron branch-point consensus sequence of FLEXI RNAs was identified by searching for the U2-type branch-point consensus using FIMO. The U12-type intron branch-point sequence of FLEXI RNAs was identified by searching for conserved sequences enriched within 40 nt of the 3’ end of the introns using MEME (https://meme-suite.org/meme/tools/meme) with settings: meme <sequence file> -rna -oc <output folder> -mod anr -nmotifs 100 -minw 6 -minsites 100 -markov order 1 -evt 0.05. The branch-point consensus sequences of U12-type FLEXI RNAs (2 with AU-AC ends and 34 with GU-AG matching the 5’ sequence of GU-AG U12-type introns) were determined by manual sequence alignment and calculation of nucleotide frequencies. Motif logos were plotted from the nucleotide frequency tables of each motif using Ceqlogo from the MEME suite (https://meme-suite.org/meme/doc/ceqlogo.html).

The length and GC content of each FLEXI was calculated from its GRCh38-annotated intron sequence. Minimum free energy (MFE) was calculated for the most stable secondary structure predicted by RNAfold for each FLEXI RNA. The significance of differences in the distributions of length, GC content and MFE of different FLEXI subsets compared to those of all other detected FLEXIs was calculated by Kolmogorov–Smirnov (KS) test. To test if the significant differences in distributions were due to the small sample size of FLEXI subsets, those with p-values <0.01 (denoted test subset) were further tested by 1,000 Monte-Carlo simulations. In each simulation, a randomly selected subset of all FLEXIs with the same number of FLEXIs as the test subset was compared to all other FLEXIs by the same KS test. If more than 50 of the 1,000 simulations (≥95%) yielded a calculated p<0.01, the significance in the original test subset was rejected and considered to be a false hit.

Reads corresponding to near full-length FLEXI RNAs (>95% of intron length) in ENCODE eCLIP datasets were identified by intersecting mapped eCLIP reads with FLEXIs using BEDTools.

### PCA, t-SNE, and ZINB-WaVE analysis

Principal component analysis (PCA) of FLEXI RNA profiles in replicate cellular RNA datasets was plotted using R, and PCA-initialized t-SNE and ZINB-WaVE analyses of these datasets were plotted using the Rtsne (https://github.com/jkrijthe/Rtsne) and zinbwave (https://github.com/drisso/zinbwave) packages in R. Batch effect correction was done by using ZINB-WaVE to include the batch information as a sample-level covariate. The normalized counts outputted by ZINB-WaVE were used for PCA analysis, and the *W* matrix outputted by ZINB-WaVE was used for visualization directly or by t-SNE.

### Linear regression model

A linear regression model used to estimate copy number per cell values for FLEXIs was built by using log_10_-transformed copy number per cell values for sncRNAs reported in the literature [33] and log_10_-transformed RPM of sncRNAs in combined datasets for each of the 4 cellular initial cellular RNA samples (S6A Fig). The linear regression was plotted in R with 95% confidence intervals. sncRNA used in the linear regression were spliceosomal snRNAs U1, U2, U4, U5, and U6; minor spliceosomal snRNAs U4ATAC and U6ATAC; U7, 7SL and 7SK RNA; MRP and RNase P RNA (S4 Table) [33].

### RBP-binding site analysis

Functional annotations and localization patterns of RBPs in the ENCODE eCLIP dataset were based on Table S2 of ref. [41]. RBPs found in stress granules were as annotated in the RNA Granule and Mammalian Stress Granules Proteome (MSGP) databases [42, 43]. The functional annotations, and localization patterns of AGO1-4 and DICER were from the UniProt database (https://www.uniprot.org) and published findings for AGO1-4 and Dicer [44–48].

Introns containing RBP-binding sites were identified by intersecting FLEXI, other short intron, or long intron coordinates with ENCODE annotated binding sites for 150 RBPs based on cross-links in eCLIP datasets with irreproducible discovery rate analysis [38]. Crosslink-centered regions for agotrons and pre-miRNAs of annotated mirtrons were identified from DICER PAR-CLIP [40] and AGO1-4 PAR-CLIP [39] datasets by using BEDTools. Any binding site that overlapped with any portion of the intron in the annotated orientation was included.

The location of the binding site with respect to normalized intron length was calculated from the distance between the mid-point of the annotated binding site based on cross-links in published CLIP datasets [38–40] relative to the 5’ and 3’ ends of the intron. Data were displayed as a density plot of the mid-point of the binding sites normalized by intron length (S11 Fig).

For hierarchical clustering of RBP-binding site co-enrichment, subsets of FLEXIs that have a binding site for each of the 47 RBPs identified as having binding sites in ≥30 different FLEXI RNAs were extracted from datasets for each of the cellular RNA samples. Over- and under-represented RBPs in each of the 47 subsets for each cellular RNA sample were identified by generating a contingency table comparing the frequency of binding sites for all 126 RBPs that have an identified binding site in a FLEXI RNA (S10 Fig) in the tested subset to its frequency in all FLEXIs. Multiple binding sites for the same RBP in the same FLEXI were counted as one binding site. p-values were calculated using Fisher’s exact test and then adjusted by the Benjamini-Hochberg procedure and log_10_-transformed. RBP-binding sites were then key-coded as not significantly different or as significantly over- or under-represented (≥2% abundance, adjusted p≤0.05) in the tested subset of FLEXIs compared to all FLEXIs. Matrices of the Gower’s distance [80] were calculated using the key-coded information from the above analyses and used as input for hierarchical clustering by the complete linkage clustering method [81]. The results were displayed as a heat map plotted using the R package Cluster and ComplexHeatmap, respectively. For hierarchical clustering analysis of the RBP-binding site co-enrichment in other short introns and long intron, 2,000 randomly selected other short or long introns for each category of intron were analyzed as above, with the process repeated 10 times for each of the cellular RNA samples.

Gene expression changes in RBP-knockdown datasets were calculated from changes in mRNA abundance measured by using DESeq2 (https://github.com/thelovelab/DESeq2) after GC correction with Salmon (https://github.com/COMBINE-lab/salmon) and conditional quantile normalization (CQN) [82]. Significantly differentially expressed (DE) genes were identified as those with |LFC|≥1 and adjusted p≤0.05 in RBP knock-down dataset compared to the control dataset. The proportion of DE genes among host genes of FLEXIs, other short introns, and long introns containing a binding site for an RBP upon knockdown was then compared to that for all genes whose transcripts lacked a binding site for that RBP by Fisher’s exact test. Data for RBPs whose knockdowns resulted in significant differences (p-value≤0.05) upon knockdowns were shown as volcano plots generated by plotting log_2_-transformed fold changes in the mRNA levels between the knockdown and control datasets versus -log_10_-transformed adjusted p-values (S13 Fig). A significant bias towards increased or decreased mRNA levels was defined as p-value ≤0.05 determined by Fisher’s exact test comparing the ratio of significantly up-regulated (log2FC>0, adjusted p≤0.05) or down-regulated (log2FC<0, adjusted p≤0.05) host genes whose FLEXIs, other short introns, or long introns contained a binding site for the RBP to that in all significantly changed genes whose transcripts lacked a binding site for the same RBP.

Changes in alternative splicing were calculated by using rMATS (https://rnaseq-mats.sourceforge.io). Coordinates of adjacent exons or of retained introns from splicing change files (MATS output) were intersected with coordinates of FLEXIs that contain a binding site for an RBP and FLEXIs that do not contain a binding site for the same RBP to obtain the inclusion level values of skipped exon (SE) and/or retained intron (RI) for each FLEXI. The results were displayed as an empirical cumulative density function (ECDF) plot showing the inclusion level difference for the skipped exon or retained intron between the knockdown and control datasets and tested for significance by Kolmogorov-Smirnov test.

### Analysis of RBP-binding sites in FLEXIs and RNA fragments of other short and long introns in nuclear and cytoplasmic fractions

FLEXIs detected by TGIRT-seq in nuclear and cytoplasmic fractions were analyzed by DESeq2. FLEXIs that have a fold change (FC) value >1.5 in one fraction compared to the other were considered as differentially enriched in the corresponding fraction and were denoted as nuclear or cytoplasmic FLEXIs, respectively. RNA fragments derived from other short and long introns were identified by intersecting mapped reads with other short intron or long intron coordinates extracted from Ensemble GRCh38 Release 93 gene annotations using BEDTools. Read pairs (Read 1 and Read 2) within the annotated introns were identified as intron RNA fragments and merged into a single bed file using BEDTools. RBP-binding sites in these RNAs were identified by intersecting the coordinates of FLEXI RNAs or merged short or long intron RNA fragments, with ENCODE annotated binding sites of 150 RBPs from eCLIP-seq with irreproducible discovery rate analysis [38] or with crosslink-centered regions identified from the DICER PAR-CLIP [40] and AGO1-4 PAR-CLIP [39] datasets by using BEDTools. Multiple binding sites for the same RBP in an intron were collapsed and counted as one binding site for that RBP.

### GO term enrichment analysis

GO term enrichment analysis of host genes encoding FLEXIs with binding sites of different RBPs was performed by using DAVID bioinformatics tools (https://david.ncifcrf.gov) with all FLEXI host genes as the background. Hierarchical clustering was performed based on p-values for GO term enrichment of host genes of FLEXIs having binding sites of the same RBPs using the gplots package in R (https://www.rdocumentation.org/packages/gplots/versions/3.1.3.1).

### Protein annotation and network analysis

Proteins identified as being encoded by oncogenes or tumor suppressor gene or mitochondrially localized proteins were based on Liu et al. [83], Zhao. et al. [84], and human MitoCarta3.0 (https://www.broadinstitute.org/mitocarta/mitocarta30-inventory-mammalian-mitochondrial-proteins-and-pathways). Protein network analysis was done by using the STRING database (https://string-db.org) with default settings.

## Supporting information

S1 Fig Classes of RNAs identified by TGIRT-seq of rRNA-depleted unfragmented cellular RNAs. Stacked bar graphs showing the percentages of sequencing reads or bases that mapped to different categories of Ensembl GRCh38 Release 93 annotated genomic features in combined technical replicates for the indicated cellular RNA samples. (A) Percentages of sequencing reads that mapped to different classes of cellular RNAs. rRNA includes cellular and mitochondrial (Mt) rRNAs, and protein-coding gene RNAs includes all transcripts of protein-coding genes from both the nuclear and mitochondrial genomes. (B) Percentages of sequencing reads that mapped to different sncRNAs. Miscellaneous (misc) RNAs include ribozymes, small NF90-associated RNAs (snaRs), promoter-associated RNAs (pRNAs), and other ncRNAs that do not fall into other categories. (C) Percentage of bases that mapped to different regions of the sense strand of protein-coding genes in the nuclear genome. Because the cellular RNAs had not been chemically fragmented, reads mapping to protein-coding genes comprised only a low percentage of total reads (0.7–5.3%). Abbreviations: CDS, coding sequences; intergenic, regions upstream or downstream of transcription start and stop sites of protein-coding genes; Intron, intronic regions; UTR, 5’- or 3’-untranslated regions.
(PDF)

S2 Fig Full-length, end-to-end sequencing reads of sncRNAs in TGIRT-seq cellular RNA datasets. Integrative Genomics Viewer (IGV) screenshots showing coverage tracks and read alignments for sncRNAs detected in TGIRT-seq datasets of unfragmented cellular RNAs. The name of the sncRNA is shown at the top with its length indicated in parentheses and the arrow indicating the 5’ to 3’ orientation of the RNA. Coverage tracks (gray) are followed by read alignments from combined technical replicates for each cellular RNA sample type color coded as shown at the top right. Reads were down sampled to a maximum of 100 for display in IGV. Gray in the coverage tracts indicates bases in the read that matched the reference base, and other colors indicate bases in the read that did not match the reference base (red, thymidine; green, adenosine; blue, cytidine; and brown, guanosine). Misincorporation at known sites of post-transcriptionally modified bases in tRNAs are highlighted in the alignments: m^1^A58: 1-methyladenosine at position 58; I: inosine. CCA indicates the post-transcriptionally added 3’ CCA sequences of tRNAs mapped against a reference set of mature tRNA sequences. NTA, non-templated nucleotides added to the 3’ end of cDNAs during TGIRT-seq library preparation.
(PDF)

S3 Fig Characteristics of FLEXI RNAs. (A) Density plots showing the length distribution of FLEXI RNAs (≤300 nt; red) that were the focus of our analysis and smaller numbers of long FLEXI RNAs (>300 nt, blue) in combined datasets for each of the 4 cellular RNA samples. The inset density plots compare the abundance (RPM) of FLEXIs ≤300 nt and >300 nt in the same datasets. (B) Three-dimensional bar graphs showing the percentage of FLEXI RNA reads ending at different positions around intron-exon junctions in datasets for each of the 4 cellular RNA samples. Arrows indicate the 5’- and 3’ splice sites (5’ SS and 3’ SS, respectively).
(PDF)

S4 Fig FLEXI RNAs corresponding to U12-type introns or introns with non-GU-AG 5’- and 3’-splice sites. (A) 5’- and 3’-splice sites (5’SS and 3’SS, respectively) and branch-point (BP) consensus sequences of GU-AG and AU-AC U12-type FLEXIs in a combined datasets for the 4 cellular RNA samples (HEK-293T, HeLa S3, K-562, and UHRR) compared to literature consensus sequences for human U12-type introns (top) [28, 30] and consensus sequences for other U12-type short (≤300 nt) and long (>300 nt) introns in the same combined datasets. The number of introns for each consensus sequence is indicated to the right of the sequence. (B) IGV screenshots showing read alignments for U12-type and non-canonical splice-site FLEXI RNAs in RNA samples from different cell lines. FLEXIs are named at the top above an arrow indicating the 5’ to 3’ orientation of the RNA with the tracks below showing gene annotations (exons, thick lines; introns, thin lines) followed by alignments of FLEXI reads in different cellular RNA samples color coded by sample type. The length of the intron that is the focus of each panel is shown above the read alignments, and the fraction of FLEXI reads (continuous reads that began and ended within 3 nt of the annotated 5’- and 3’-splice sites) for that intron in different cell lines is shown within the read alignments. Reads were down sampled to a maximum of 100 for display in IGV. NTA, non-templated nucleotides added to the 3’ end of cDNAs during TGIRT-seq library preparation.
(PDF)

S5 Fig FLEXI RNAs are degraded by 5’- and 3’-exonucleases. DNase-treated, size-selected (≤200 nt) HEK-293T and UHRR RNAs were incubated with (+) or without (mock treatment; -) Terminator and RNase R exonucleases, and the products for 6 different FLEXI RNAs and 3 sncRNAs products were analyzed by RT-qPCR using SYBR Green with gene-specific DNA oligonucleotide primers near the 3’ end of the RNA (S9 Table; see Materials and Methods). (A) Cycle threshold (CT) values for qPCRs of exonuclease- and mock-treated FLEXIs and sncRNAs (top panel) and calculated percentages of RNAs remaining after exonuclease treatment relative to the parallel mock treatment (bottom panel). Three experimental replicates of exonuclease digestion and matched mock-treatments were performed for each RNA type. Each experimental replicate was quantified as the CT mean of 3 qPCR technical replicates and displayed as a box plot. The specificity of PCR products was confirmed by extracting gel bands corresponding to representative qPCR amplicons followed by Sanger sequencing directly or after TOPO-TA cloning (S10 Table). (B) Denaturing PAGE of synthetic 3I_RAN FLEXI RNA that was (i) circularized *in vitro* by T4 RNA Ligase 1; (ii) linear with a 5’ phosphate required for circularization; or (iii) linear with a 5’ OH after mock (-) or exonuclease treatment (+) with Terminator and RNase R exonucleases. Size markers in the left-most lane are a Low range ssRNA ladder (New England Biolabs), and those in the right-most lane are an RNA Century Ladder (Ambion). Circular and linear forms are indicated by arrowheads color coded as shown to the right of the gel.
(PDF)

S6 Fig Estimates of FLEXI RNA abundance using a linear regression model and droplet digital PCR. (A) Linear regression models (*lrm*) of molecule per cell values based on RPM values in TGIRT-seq datasets of sncRNAs with literature reported copy number per cell values. The scatter plots show the relationship between log_10_-transformed copy number per cell values reported in the literature [33] and log_10_-transformed RPM of sncRNAs in combined TGIRT-seq datasets for each of the 4 cellular RNA samples. The linear regression was plotted as a light blue line with 95% confidence intervals shown as dashed blue lines. sncRNAs used in the linear regression were major spliceosomal snRNAs U1, U2, U4, U5, and U6; minor spliceosomal snRNAs U4ATAC and U6ATAC; U7, 7SL and 7SK RNA; MRP and RNase P RNA (S4 Table)[33]. Pearson (*r*) correlation coefficients are shown in the upper left of each panel. The Table below the plots shows literature values for three sncRNAs used as standards for droplet digital PCR (ddPCR) and copy number per cell values for these RNAs in different cellular RNA samples based on the *lrm*. (B) Comparison of abundance estimates for FLEXI RNAs based on the TGIRT-seq *lrm* and droplet digital PCR (ddPCR). The left-hand columns (TGIRT-seq) show RPM and copy number per cell values of FLEXIs estimated using the TGIRT-seq *lrm* for the cellular RNA samples. The right-hand columns (ddPCR) show copy number per cell values based on ddPCR abundance relative to 3 different sncRNA standards run in parallel (U7, SNORD14B, and SNORD44). For ddPCR, reverse transcription was performed on DNase-treated, size-selected (≤200 nt) RNA preparations using Maxima H reverse transcriptase with a gene-specific primer at the 3’ end of the putative FLEXI or sncRNA (S9 Table). ddPCR was then performed using primer sets within the FLEXI sequence or spanning the 5’-exon-FLEXI junction, with the latter subtracted from the FLEXI signal when applicable (S9 Table). Copy number per cell values for FLEXIs were calculated based on copy number per cell values for U7, SNORD14B, and SNORD44 in each cellular RNA sample obtained using the *lrm* (Table at the bottom of panel A).
(PDF)

S7 Fig Reproducibility of cell-type specific expression patterns of FLEXIs. (A) and (B) PCA-initialized t-SNE [85] and ZINB-WaVE [34] analysis of FLEXI RNAs detected at ≥1 read (panel A) or ≥0.01 RPM (panel B) in biological and technical replicates of TGIRT-seq for rRNA-depleted unfragmented RNAs from different cellular RNA samples (S1 Table). t-SNE and ZINB-WaVE are widely used for the analysis of single cell RNA-seq datasets with zero-inflated counts. The top row of each panel shows clustering of datasets for technical replicates for different cell types used in this study. The middle and bottom rows of each panel show clustering of datasets for biological replaces of rRNA-depleted unfragmented HEK-293T, K-562, MDA-MB-231, and UHRR RNAs from this study (biological replicate 1 (Bio 1, circles) and other studies (biological replicate 2 (Bio 2), triangles) before (middle) and after (bottom) batch effect correction by ZINB-WaVE. The compared datasets are listed in S1 Table.
(PDF)

S8 Fig FLEXI RNAs from the same host gene can differ widely in expression levels. (A) IGV screenshots showing examples of differences in abundance of FLEXI RNAs transcribed from the same host gene reflecting post-transcriptional differences in splicing efficiency, alternative splicing, or differential stability of FLEXI RNAs. Gene maps for different RNA isoforms generated by alternative splicing of FLEXI RNAs are shown at the top and bottom with introns from the 5’ to 3’ RNA end named A to F. Reads mapping to exons or non-FLEXI introns were omitted for clarity. Reads for FLEXIs from different cellular RNA samples are color coded as shown in the Figure. NTA, non-templated nucleotides added to the 3’ end of cDNAs during TGIRT-seq library preparation. Two FLEXIs in the top panel had indels at short runs of 3’ U residues, likely reflecting a previously reported tendency for TGIRT enzyme slippage at such locations [86]. (B) IGV screen shots showing differences in expression levels for long genes with labels omitted for better visualization of multiple FLEXIs from the same host gene.
(PDF)

S9 Fig Heat map and box plots comparing the relative abundance of FLEXIs in TGIRT-seq biological and technical replicates of cellular RNA samples. (A) Heatmap comparing the abundance of 200 FLEXI RNAs in biological replicates (1 and 2, top) and technical replicates (separated by black vertical lines) of TGIRT-seq datasets for rRNA-depleted unfragmented cellular RNA samples (S1 Table). FLEXI abundance was color coded by log_2_-transformed RPM values. Clusters of FLEXIs that were highly expressed in different cell lines are highlighted in boxes with yellow borders. (B) Box plots showing examples of the relative abundance of cell-type specific FLEXIs in the TGIRT-seq datasets of panel A. UHRR, which is comprised of RNAs from multiple human cell lines, was omitted from this panel.
(PDF)

S10 Fig Quantitation of RBP-binding sites in different classes of intron RNAs. Bar graphs showing the number of (A) FLEXI RNAs, (B) Other Ensembl GRCh38-annotated short introns (≤300 nt), and (C) Ensembl GRCh38-annotated long introns (>300 nt) that have a CLIP-seq-identified binding site for the indicated RBP in a merged dataset for the K-562, HEK-293T, HeLa S3, and UHRR cellular RNA samples. Bars graphs are color coded by RBP function as shown at the top. Asterisks above the bars in panels B and C indicate the 53 proteins identified as binding ≥30 different FLEXIs in Fig 4A.
(PDF)

S11 Fig Locations of RBP-binding sites in FLEXIs and other classes of introns. Density plots of ENCODE eCLIP-identified RBP-binding sites in K-562 or Hep G2 cells and PAR-CLIP identified binding sites for AGO1-4 and DICER in HEK-293 cells [38–40] for the 53 RBPs with binding sites for ≥30 FLEXIs (≤300 nt; red line) compared to other short introns (≤300 nt; blue line) and long introns (>300 nt; back lines). RBP-binding sites overlapping introns (≥1 nt) were identified by intersecting annotated RBP-binding sites with intron coordinates using BEDTools and plotted as the mid-point of the annotated RBP-binding sites normalized as a percentage of intron length with 0% and 100% corresponding to the 5’ and 3’ end of the intron, respectively. Vertical red dashed lines indicate the position of peaks in the density plots for FLEXI RNAs. RBP names are color coded by protein function as indicated at the bottom of the Figure. Blank spaces were left for datasets that were not available for one of the two cell lines used to obtain the eCLIP datasets.
(PDF)

S12 Fig Enrichment of sequencing reads corresponding to near full-length FLEXIs found in ENCODE eCLIP datasets despite RNase treatment used in processing cross-linked samples. eCLIP datasets for Hep G2 and K-562 cells were downloaded from ENCODE for all 51 RBPs with binding sites for ≥30 FLEXIs, and reads in those datasets that were ≥95% of the length of a FLEXI were identified by intersecting the eCLIP reads with FLEXI coordinates. Data are shown as bar graphs for fold enrichment of ≥95% full-length intron reads for each RBP in the eCLIP dataset versus the control antibody dataset. The dashed line indicates 2-fold enrichment. Names of proteins are color coded by protein function as indicated below the bar graph. For those RBPs in which more than one dataset was available, mean values were calculated for the fold enrichment in eCLIP replicates over controls. Only those RBPs that have ≥2-fold enrichment are shown (S5 Table).
(PDF)

S13 Fig Changes in mRNA levels of host genes of FLEXIs, other short, and long introns with binding sites for different RBPs in RBP-knockdown versus control datasets. Volcano plots showing -log_10_-transformed adjusted p-values versus log_2_-transformed fold changes for ENSEMBL-annotated genes in ENCODE knockdown versus control datasets for the indicated RBPs in K-562 and Hep G2 cells. Host genes of (A) FLEXIs, (B) other short introns, and (C) long introns that contain an annotated binding site for the indicated RBP that have significant differential expression (DE) in mRNA levels (adjusted p≤0.05, |LFC|≥1) in the knockdown datasets are shown as red dots. Other genes with or without significant expression changes are shown as black or gray dots, respectively. RBPs whose knockdown resulted in a significant bias towards increased or decreased mRNA levels from host genes encoding FLEXIs, other short introns, and long introns with an annotated binding site for the RBP compared to genes whose transcript lack an annotated binding site for the same RBP are indicated by up (light blue) or down (red) arrows, respectively next to the RBP name. For these comparisons, significant bias is defined as p-value ≤0.05 determined by Fisher’s exact test comparing the ratio of significantly up-regulated (log_2_FC>0, adjusted p≤0.05) or down-regulated (log_2_FC<0, adjusted p≤0.05) host genes whose FLEXIs, other short introns, or long introns contain an annotated binding site for the RBP to those in all significantly changed genes whose transcripts lack an annotated binding site for the same RBP. RBP knockdowns that resulted in significant changes in mRNA levels from host genes containing a FLEXI, other short introns, and long introns with a binding site for the knocked down RBP, but no significant directional bias, are indicated by a (gray) bi-directional arrow next to the RBP name. Plots are shown only from those RBPs whose knockdown resulted in a significant difference (p≤0.05) in the number of DE genes whose FLEXIs, other short introns, or long introns contain an annotated binding site for the RBP. Datasets that were not available for an RBP in one of the two cell lines were left as a blank space. RBP names are color coded by protein function as shown at the bottom of the Figure.
(PDF)

S14 Fig Splicing changes at or adjacent to different classes of introns in RBP-knockdown versus control datasets. Splicing changes for (A) FLEXIs, (B) other short introns, or (C) long introns in ENCODE knockdown datasets for Hep G2 and K-562 cells were calculated using rMATS (https://rnaseq-mats.sourceforge.io). The Figure shows Empirical Cumulative Distribution Function (ECDF) plots for inclusion of retained introns (RI) or skipped exons (SE) adjacent to introns that have (red) or do not have (gray) a CLIP-seq-identified binding site for the indicated RBP in ENCODE knockdown datasets. Red curves shifted to the right or left of the control (gray) indicate an increase or decrease, respectively, in retained introns and skipped exons as indicated at the top of each set of plots. Statistical significance was calculated by Kolmogorov-Smirnov test. Plots are shown only from those RBPs whose knockdown resulted in a significant change (p≤0.05). Names of RBPs are color coded by protein function as indicated in the Figure. Blank spaces were left for datasets that were not available for an RBP in one of the two cell lines. Axes labels are shown in the key at the bottom right.
(PDF)

S15 Fig Hierarchical clustering of FLEXIs in different cell lines based on over- and under-represented RBP-binding sites. FLEXIs containing at least one annotated binding site for any RBP in any of the 4 cell lines (1,261 to 2,404 FLEXIs in different cell lines) were used in this analysis. For hierarchical clustering, subsets of FLEXIs (6 to 1,086) that have binding sites for each of the 47 non-core spliceosomal RBPs with binding sites for ≥30 different FLEXI in a merged TGIRT-seq dataset for the 4 cell lines were extracted for each of the cellular RNA sample types. Contingency tables were then generated by comparing the frequency of binding sites for all 126 RBPs that have an identified binding site in a FLEXI RNA in each of these subsets to those in all FLEXI RNAs. Multiple binding sites for the same RBP in the same FLEXI were counted as one binding site. p-values for over- and under-represented RBP-binding sites calculated by Fisher’s exact test were adjusted by the Benjamini-Hochberg procedure. RBP-binding sites (≥2% abundance) were then key-coded as not significantly different or as significantly over- or under-represented in the tested subset of FLEXIs compared to all FLEXIs (adjusted p≤0.05 calculated by Fisher’s exact test and adjusted by the Benjamini-Hochberg procedure), and the key-coded information was used to construct matrices of the Gower’s distance between the 47 subsets in each of the cellular RNA samples that were used as input for hierarchical clustering by the complete linkage clustering method [81]. The results were displayed as a two-dimensional heat map to identify subsets of FLEXIs showing similar patterns of significantly over- and under-represented RBP-binding sites in each of the cellular RNA samples, with the color scale at the bottom based on -log_10_-transformed adjusted p-values. Significantly co-enriched RBPs are indicated by an X in the heatmap box, and clusters of co-enriched RBPs are delineated in larger boxes. RBP names are color coded by protein function as indicated at the bottom of the Figure.
(PDF)

S16 Fig Enriched RBP-binding sites and characteristics of subsets of FLEXIs with a binding site for each of the RBPs in Clusters I-VI. Scatter plots and density plots are shown for FLEXIs with binding sites for each protein in (A) Cluster I, (B) Cluster II, (C) Cluster III, (D) Cluster IV, (E) Cluster V, and (F) Cluster VI. In the scatter plots (left), RBPs whose binding sites were significantly over- or under-represented compared to those for other RBPs in the subset of FLEXIs compared to all other FLEXIs (≥2% abundance, p≤0.05 calculated by Fisher’s exact test and adjusted by the Benjamini-Hochberg procedure) are labeled by name color coded by protein function as shown at the bottom of the Figure. The density distribution plots (right) compare the length, GC content, and MFE for the most stable secondary structure predicted by RNAfold for subsets of FLEXIs with binding sites for each RBP associated with Clusters I to VI (red) compared to all other FLEXIs (black). The number of FLEXIs comprising each subset is indicated in parentheses next to the name of the RBP. p-values are shown at the top left of those density plots in which the distribution for the subset of FLEXIs differed significantly from other FLEXIs (p<0.01 and FDR≤0.05 as determined by 1,000 Monte-Carlo simulations).
(PDF)

S17 Fig Patterns of overlapping RBP-binding sites in FLEXI RNAs. Heat maps of FLEXI RNAs associated with Clusters I-VI that have overlapping binding sites for each of the 47 non-core spliceosomal RBPs with a binding site in ≥30 FLEXIs (columns and rows, respectively). The number of FLEXIs containing overlapping binding sites for each compared pair of RBPs (columns and rows) were log_10_-transformed, clustered and color-coded. Names of RBPs associated with Clusters I-VI are color coded as shown at the bottom of the Figure.
(PDF)

S18 Fig Cell-type specific FLEXI RNAs and their host genes in MCF7 and MDA-MB-231 cancer cell lines. UpSet plots showing the distribution of (A) FLEXI RNAs, (B) FLEXI host genes, (C) FLEXI host oncogenes, and (D) FLEXI host tumor suppressor genes in MCF7 cells and MDA-MB-231 cells, compared to those in a combined dataset (Other) for HEK-293T, HeLa S4, K-562 and UHRR cellular RNA samples. FLEXI RNAs from known oncogenes [83] or tumor suppressor genes [84] that were detected only in MCF7 and/or MDA-MB-231 are listed below the plots. ATF3 and KLF4 were annotated as both oncogenes and tumor suppressor genes [83, 84].
(PDF)

S19 Fig Analysis of RNAs in nuclear and cytoplasmic fractions from cultured cells. Scatter plots comparing enrichment of all and subtypes of cellular RNAs in cytoplasmic and nuclear fractions from HeLa S3, K-562, MDA-MB-231, and MCF7 cells. RNA subtypes are color coded as shown to the right of the scatter plots. Mitochondrial RNAs, tRNAs, Vault RNAs, Y RNA, and 7SL RNA were enriched in the cytoplasmic RNA fraction, while 7SK RNA and snRNAs were enriched in the nuclear RNA fraction. Most snoRNAs (357 to 457 in different cell types) had higher normalized counts in nuclear fractions than in cytoplasmic fractions with only 20–49 snoRNAs having higher normalized counts in cytoplasmic fractions.
(PDF)

S20 Fig Clustering analysis of RNAs in nuclear and cytoplasmic fractions from cultured cells. (A) and (B) Principal Component Analysis (PCA) plots for RNAs detected by TGIRT-seq in whole-cell (squares), nuclear fractions (Nucleus; triangles), and cytoplasmic fractions (Cytoplasm; circles) for HeLa S3, K-562, MCF7 and MDA-MB-231 cells color coded as shown in the Figure. (C) Heatmap of Euclidean distance between datasets shaded as being more (darker blue) or less (lighter blue) similar as shown by color scale in the Figure.
(PDF)

S21 Fig Characterization of RBP-binding sites in intron RNAs encoded by different categories of host genes. (A) Numbers and percentages of host genes encoding mitochondrial proteins annotated in MitoCarta3.0 or cytoplasmic or mitochondrial ribosomal proteins annotated in GRCh38 that contain FLEXIs (top), other short introns (middle), or long introns (bottom) with binding sites for Cluster I RBPs. (B) Numbers and percentages of all host genes for different subsets of introns compared to those for all GRCh38 annotated protein-coding genes. (C) Density plots showing the average coverage of RBP-binding sites across the gene body (Exons CDS + UTRs; black) or intron body (red) calculated using Picard tools normalized by percent length. In the gene body plots, 0% and 100% indicate the 5’ and 3’ ends the mRNAs, respectively. In the intron body plots, 0% indicates the 5’ end of the first intron and 100% indicates the 3’ end of the last intron of the same gene.
(PDF)

S1 Table Summary of TGIRT-seq datasets obtained in this study. (PDF)

S2 Table Genomic locations and characteristics of FLEXIs detected in human cell lines, UHRR, and human plasma. (XLSX)

S3 Table qPCR results for S5 Fig. (XLSX)

S4 Table Abundance of sncRNAs (RPM) detected by TGIRT-seq in cellular RNA samples compared to literature copy number per cell values for these RNAs. (PDF)

S5 Table Enrichment of FLEXI reads in ENCODE eCLIP dataset for S12 Fig. (XLSX)

S6 Table Cellular functions of RBPs with binding sites for ≥30 different FLEXIs. (PDF)

S7 Table Genomic locations and characteristics of FLEXIs detected in nuclear and cytoplasmic fractions in different cell lines. (XLSX)

S8 Table Oligonucleotides used for construction of TGIRT-seq libraries. (PDF)

S9 Table Oligonucleotides used for ddPCR and exonuclease digestion assays. (PDF)

S10 Table Sanger sequencing of qPCR amplicons in S5 and S6 Figs. (XLSX)
