# Supplementary material for: Human cells contain myriad excised linear intron RNAs with links to gene regulation and potential utility as biomarkers
Source: PLoS Genet. 2024 Sep 26;20(9):e1011416. doi: 10.1371/journal.pgen.1011416 (PMC11460701; doi:10.1371/journal.pgen.1011416)
Supplement: S1 Fig — Stacked bar graphs showing the percentages of sequencing reads or bases that mapped to different categories of Ensembl GRCh38 Release 93 annotated genomic features in combined technical replicates for the indicated cellular RNA samples. (A) Percentages of sequencing reads that mapped to different classes of cellular RNAs. rRNA includes cellular and mitochondrial (Mt) rRNAs, and protein-coding gene RNAs includes all transcripts of protein-coding genes from both the nuclear and mitochondrial genomes. (B) Percentages of sequencing reads that mapped to different sncRNAs. Miscellaneous (misc) RNAs include ribozymes, small NF90-associated RNAs (snaRs), promoter-associated RNAs (pRNAs), and other ncRNAs that do not fall into other categories. (C) Percentage of bases that mapped to different regions of the sense strand of protein-coding genes in the nuclear genome. Because the cellular RNAs had not been chemically fragmented, reads mapping to protein-coding genes comprised only a low percentage of total reads (0.7–5.3%). Abbreviations: CDS, coding sequences; intergenic, regions upstream or downstream of transcription start and stop sites of protein-coding genes; Intron, intronic regions; UTR, 5’- or 3’-untranslated regions. (PDF) [file pgen.1011416.s001.pdf]

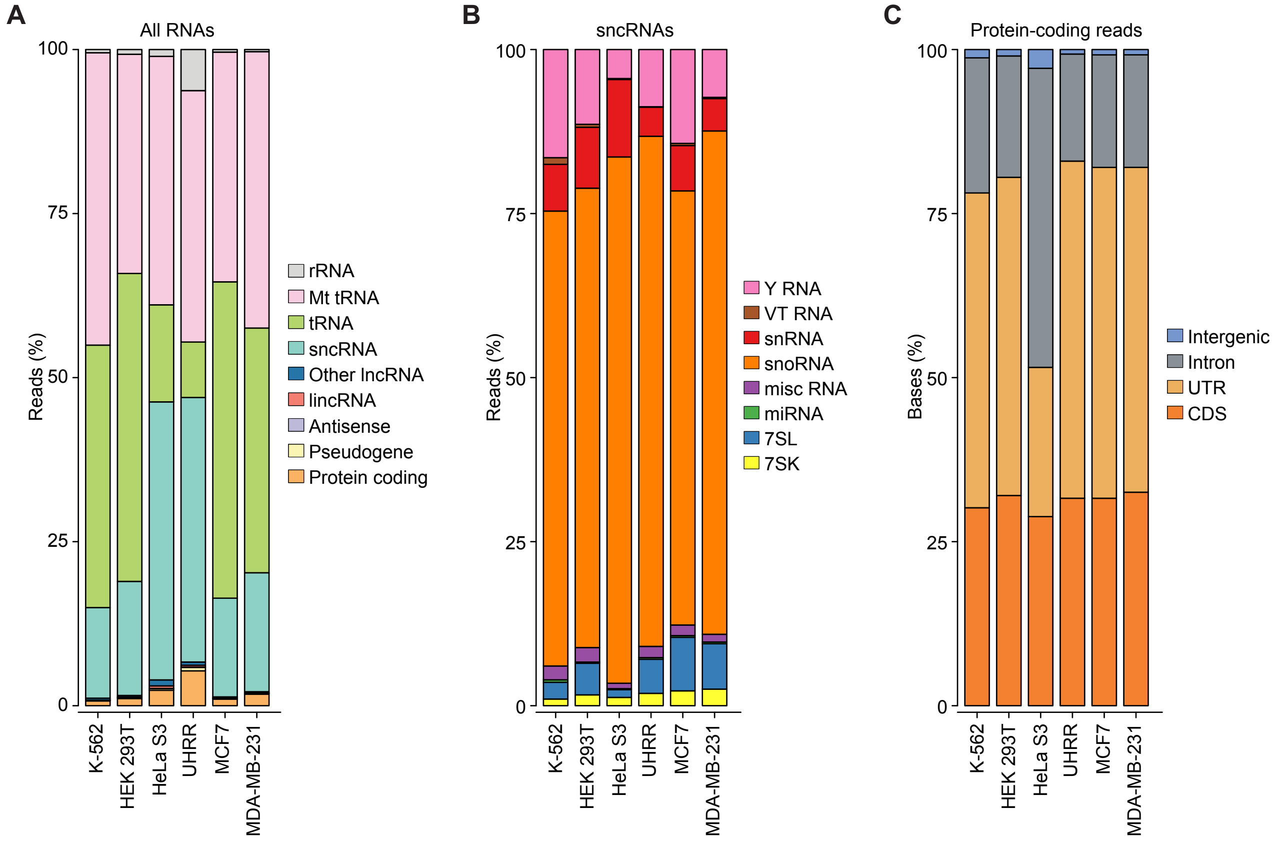

S1 Fig. Classes of RNAs identified by TGIRT-seq of rRNA-depleted unfragmented cellular RNAs. Stacked bar graphs showing the percentages of sequencing reads or bases that mapped to different categories of Ensembl GRCh38 Release 93 annotated genomic features in combined technical replicates for the indicated cellular RNA samples. **(A)** Percentages of sequencing reads that mapped to different classes of cellular RNAs. rRNA includes cellular and mitochondrial (Mt) rRNAs, and protein-coding gene RNAs includes all transcripts of protein-coding genes from both the nuclear and mitochondrial genomes. **(B)** Percentages of sequencing reads that mapped to different sncRNAs. Miscellaneous (misc) RNAs include ribozymes, small NF90-associated RNAs (snaRs), promoter-associated RNAs (pRNAs), and other ncRNAs that do not fall into other categories. **(C)** Percentage of bases that mapped to different regions of the sense strand of protein-coding genes in the nuclear genome. Because the cellular RNAs had not been chemically fragmented, reads mapping to protein-coding genes comprised only a low percentage of total reads (0.7-5.3%). Abbreviations: CDS, coding sequences; intergenic, regions upstream or downstream of transcription start and stop sites of protein-coding genes; Intron, intronic regions; UTR, 5'- or 3'-untranslated regions.
