# Supplementary material for: Human cells contain myriad excised linear intron RNAs with links to gene regulation and potential utility as biomarkers
Source: PLoS Genet. 2024 Sep 26;20(9):e1011416. doi: 10.1371/journal.pgen.1011416 (PMC11460701; doi:10.1371/journal.pgen.1011416)
Supplement: S2 Fig — Integrative Genomics Viewer (IGV) screenshots showing coverage tracks and read alignments for sncRNAs detected in TGIRT-seq datasets of unfragmented cellular RNAs. The name of the sncRNA is shown at the top with its length indicated in parentheses and the arrow indicating the 5’ to 3’ orientation of the RNA. Coverage tracks (gray) are followed by read alignments from combined technical replicates for each cellular RNA sample type color coded as shown at the top right. Reads were down sampled to a maximum of 100 for display in IGV. Gray in the coverage tracts indicates bases in the read that matched the reference base, and other colors indicate bases in the read that did not match the reference base (red, thymidine; green, adenosine; blue, cytidine; and brown, guanosine). Misincorporation at known sites of post-transcriptionally modified bases in tRNAs are highlighted in the alignments: m1A58: 1-methyladenosine at position 58; I: inosine. CCA indicates the post-transcriptionally added 3’ CCA sequences of tRNAs mapped against a reference set of mature tRNA sequences. NTA, non-templated nucleotides added to the 3’ end of cDNAs during TGIRT-seq library preparation. (PDF) [file pgen.1011416.s002.pdf]

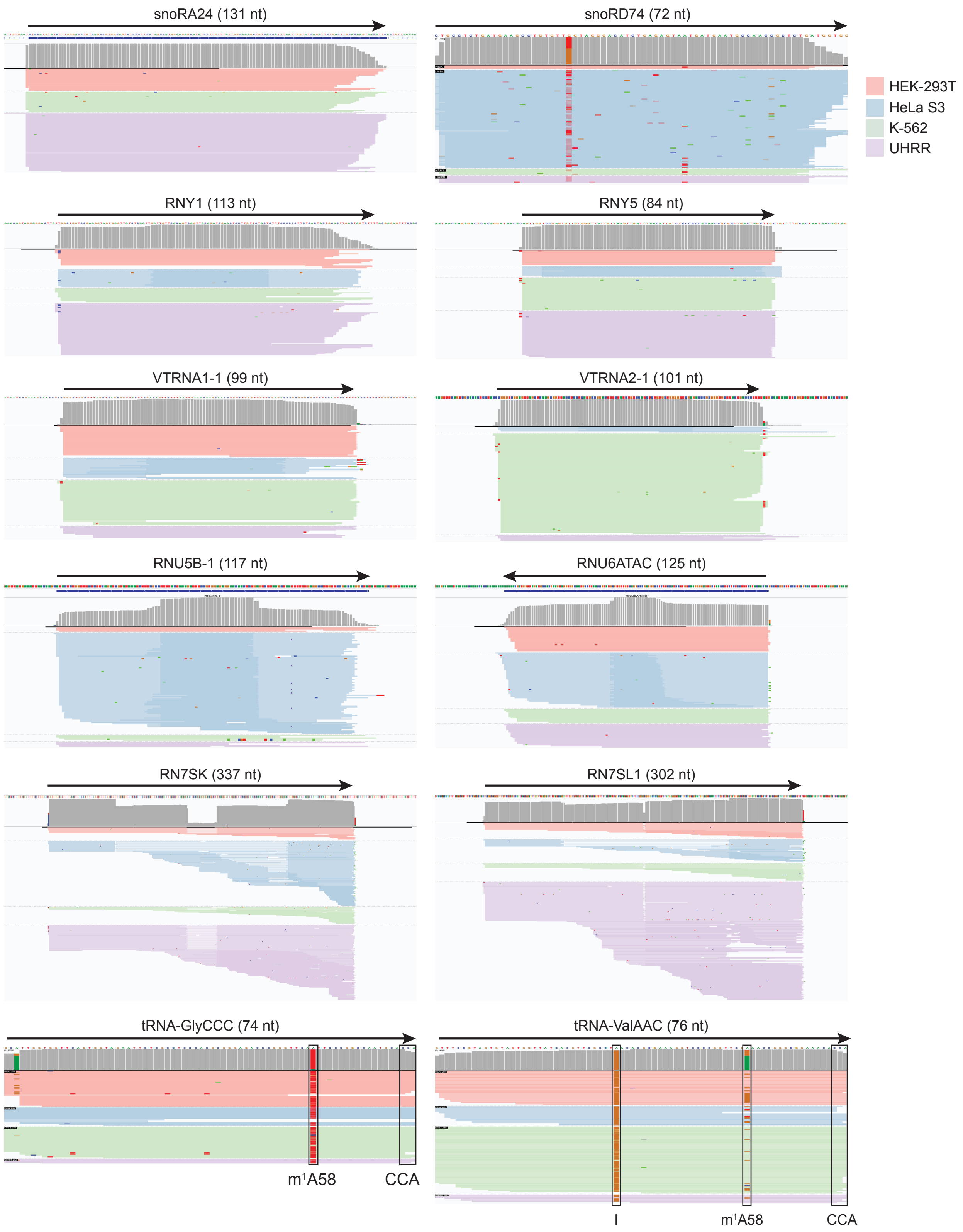

S2 Fig. Full-length, end-to-end sequencing reads of sncRNAs in TGIRT-seq cellular RNA datasets. Integrative Genomics Viewer (IGV) screenshots showing coverage tracks and read alignments for sncRNAs detected in TGIRT-seq datasets of unfragmented cellular RNAs. The name of the sncRNA is shown at the top with its length indicated in parentheses and the arrow indicating the 5' to 3' orientation of the RNA. Coverage tracks (gray) are followed by read alignments from combined technical replicates for each cellular RNA sample type color coded as shown at the top right. Reads were down sampled to a maximum of 100 for display in IGV. Gray in the coverage tracts indicates bases in the read that matched the reference base, and other colors indicate bases in the read that did not match the reference base (red, thymidine; green, adenosine; blue, cytidine; and brown, guanosine). Misincorporation at known sites of post-transcriptionally modified bases in tRNAs are highlighted in the alignments: m^{1}A58: 1-methyladenosine at position 58; I: inosine. CCA indicates the post-transcriptionally added 3' CCA sequences of tRNAs mapped against a reference set of mature tRNA sequences. NTA, non-templated nucleotides added to the 3' end of cDNAs during TGIRT-seq library preparation.
