# Supplementary material for: Human cells contain myriad excised linear intron RNAs with links to gene regulation and potential utility as biomarkers
Source: PLoS Genet. 2024 Sep 26;20(9):e1011416. doi: 10.1371/journal.pgen.1011416 (PMC11460701; doi:10.1371/journal.pgen.1011416)
Supplement: S3 Fig — (A) Density plots showing the length distribution of FLEXI RNAs (≤300 nt; red) that were the focus of our analysis and smaller numbers of long FLEXI RNAs (>300 nt, blue) in combined datasets for each of the 4 cellular RNA samples. The inset density plots compare the abundance (RPM) of FLEXIs ≤300 nt and >300 nt in the same datasets. (B) Three-dimensional bar graphs showing the percentage of FLEXI RNA reads ending at different positions around intron-exon junctions in datasets for each of the 4 cellular RNA samples. Arrows indicate the 5’- and 3’ splice sites (5’ SS and 3’ SS, respectively). (PDF) [file pgen.1011416.s003.pdf]

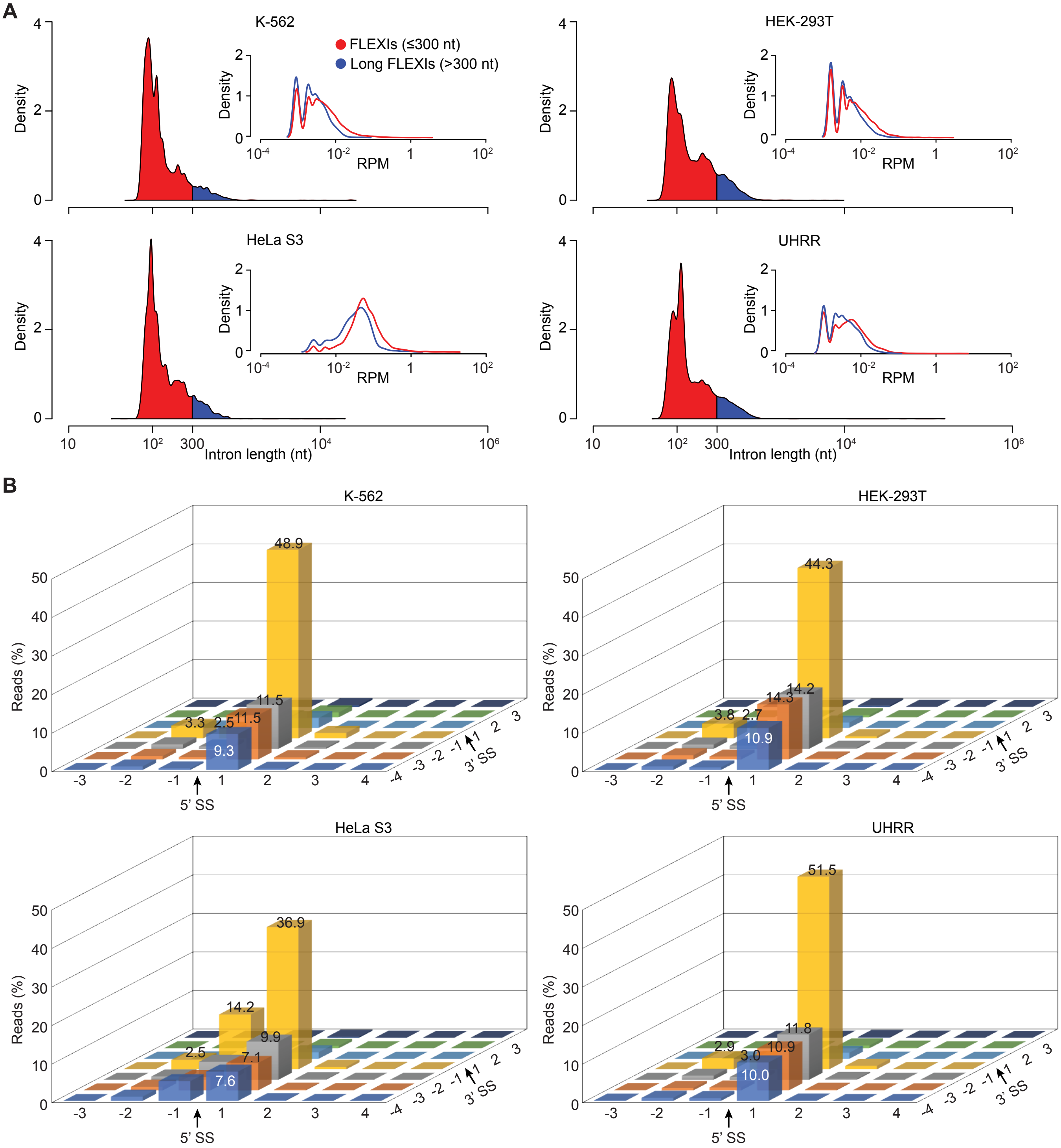

S3 Fig. Characteristics of FLEXI RNAs.
(A) Density plots showing the length distribution of FLEXI RNAs (≤300 nt; red) that were the focus of our analysis and smaller numbers of long FLEXI RNAs (>300 nt, blue) in combined datasets for each of the 4 cellular RNA samples. The inset density plots compare the abundance (RPM) of FLEXIs ≤300 nt and >300 nt in the same datasets. **(B)** Three-dimensional bar graphs showing the percentage of FLEXI RNA reads ending at different positions around intron-exon junctions in datasets for each of the 4 cellular RNA samples. Arrows indicate the 5'- and 3' splice sites (5' SS and 3' SS, respectively).
