# Supplementary material for: Human cells contain myriad excised linear intron RNAs with links to gene regulation and potential utility as biomarkers
Source: PLoS Genet. 2024 Sep 26;20(9):e1011416. doi: 10.1371/journal.pgen.1011416 (PMC11460701; doi:10.1371/journal.pgen.1011416)
Supplement: S5 Fig — DNase-treated, size-selected (≤200 nt) HEK-293T and UHRR RNAs were incubated with (+) or without (mock treatment; -) Terminator and RNase R exonucleases, and the products for 6 different FLEXI RNAs and 3 sncRNAs products were analyzed by RT-qPCR using SYBR Green with gene-specific DNA oligonucleotide primers near the 3’ end of the RNA (S9 Table; see Materials and Methods). (A) Cycle threshold (CT) values for qPCRs of exonuclease- and mock-treated FLEXIs and sncRNAs (top panel) and calculated percentages of RNAs remaining after exonuclease treatment relative to the parallel mock treatment (bottom panel). Three experimental replicates of exonuclease digestion and matched mock-treatments were performed for each RNA type. Each experimental replicate was quantified as the CT mean of 3 qPCR technical replicates and displayed as a box plot. The specificity of PCR products was confirmed by extracting gel bands corresponding to representative qPCR amplicons followed by Sanger sequencing directly or after TOPO-TA cloning (S10 Table). (B) Denaturing PAGE of synthetic 3I_RAN FLEXI RNA that was (i) circularized in vitro by T4 RNA Ligase 1; (ii) linear with a 5’ phosphate required for circularization; or (iii) linear with a 5’ OH after mock (-) or exonuclease treatment (+) with Terminator and RNase R exonucleases. Size markers in the left-most lane are a Low range ssRNA ladder (New England Biolabs), and those in the right-most lane are an RNA Century Ladder (Ambion). Circular and linear forms are indicated by arrowheads color coded as shown to the right of the gel. (PDF) [file pgen.1011416.s005.pdf]

A

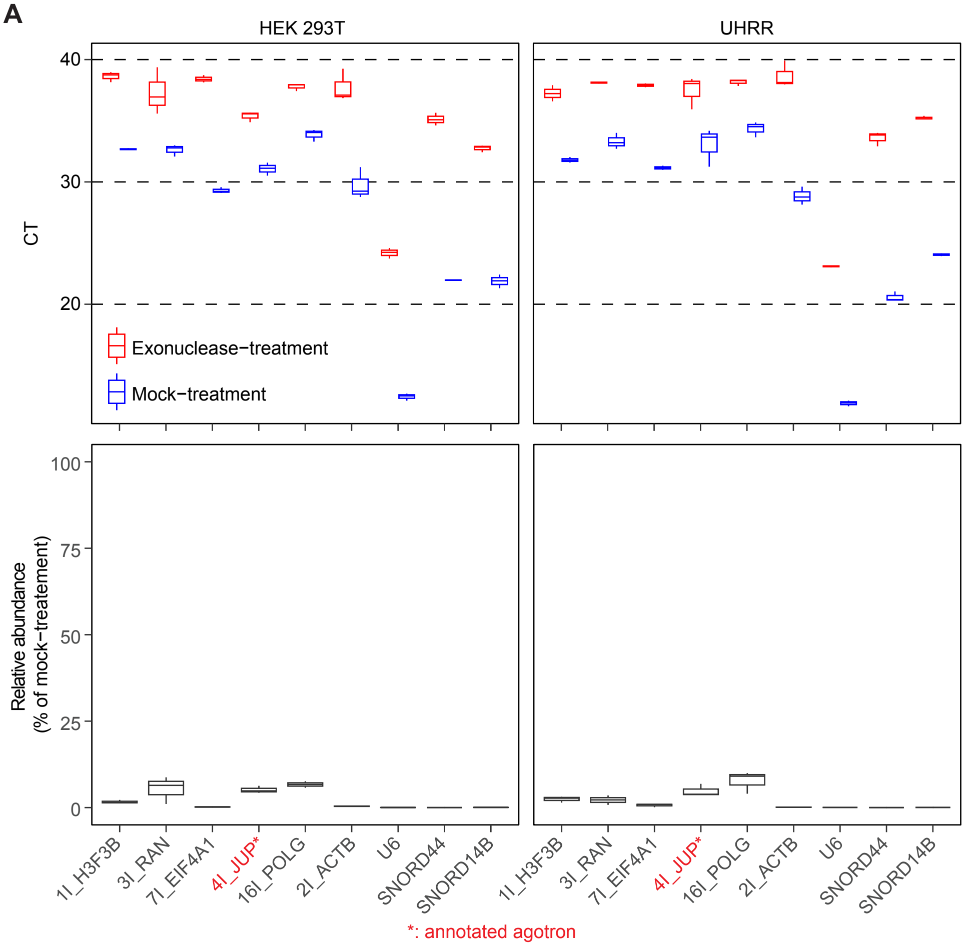
HEK 293T
UHRR
40
30
20
CT
Exonuclease−treatment
Mock−treatment
100
75
50
25
0
Relative abundance
(% of mock−treatement)
1I_H3F3B
3I_RAN
7I_EIF4A1
4I_JUP*
16I_POLG
2I_ACTB
U6
SNORD44
SNORD14B
*: annotated agotron

B

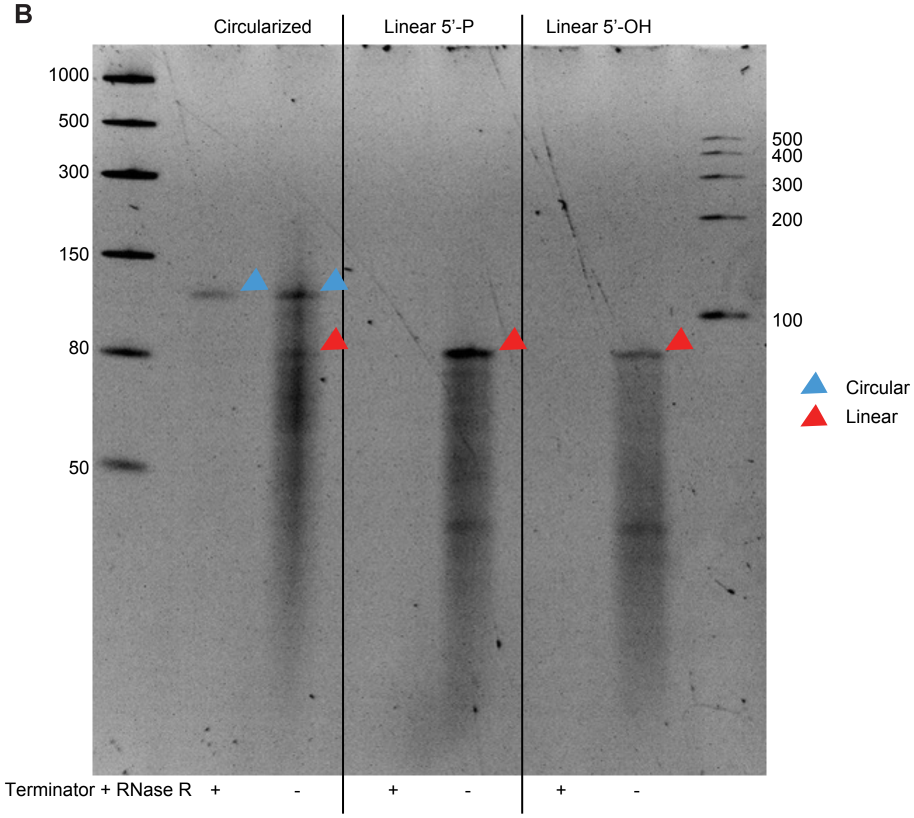
Circularized
Linear 5'-P
Linear 5'-OH
1000
500
300
150
80
50
500
400
300
200
100
Circular
Linear
Terminator + RNase R
+
-
+
-
+
-

S5 Fig. FLEXI RNAs are degraded by 5'- and 3'-exonucleases.

DNase-treated, size-selected (≤200 nt) HEK-293T and UHRR RNAs were incubated with (+) or without (mock treatment; -) Terminator and RNase R exonucleases, and the products for 6 different FLEXI RNAs and 3 sncRNAs products were analyzed by RT-qPCR using SYBR Green with gene-specific DNA oligonucleotide primers near the 3' end of the RNA (S9 Table; see Materials and Methods). **(A)** Cycle threshold (CT) values for qPCRs of exonuclease- and mock-treated FLEXIs and sncRNAs (top panel) and calculated percentages of RNAs remaining after exonuclease treatment relative to the parallel mock treatment (bottom panel). Three experimental replicates of exonuclease digestion and matched mock-treatments were performed for each RNA type. Each experimental replicate was quantified as the CT mean of 3 qPCR technical replicates and displayed as a box plot. The specificity of PCR products was confirmed by extracting gel bands corresponding to representative qPCR amplicons followed by Sanger sequencing directly or after TOPO-TA cloning (S10 Table). **(B)** Denaturing PAGE of synthetic 3I_RAN FLEXI RNA that was (i) circularized in vitro by T4 RNA Ligase 1; (ii) linear with a 5' phosphate required for circularization; or (iii) linear with a 5' OH after mock (-) or exonuclease treatment (+) with Terminator and RNase R exonucleases. Size markers in the left-most lane are a Low range ssRNA ladder (New England Biolabs), and those in the right-most lane are an RNA Century Ladder (Ambion). Circular and linear forms are indicated by arrowheads color coded as shown to the right of the gel.
