# Supplementary material for: Human cells contain myriad excised linear intron RNAs with links to gene regulation and potential utility as biomarkers
Source: PLoS Genet. 2024 Sep 26;20(9):e1011416. doi: 10.1371/journal.pgen.1011416 (PMC11460701; doi:10.1371/journal.pgen.1011416)
Supplement: S6 Fig — (A) Linear regression models (lrm) of molecule per cell values based on RPM values in TGIRT-seq datasets of sncRNAs with literature reported copy number per cell values. The scatter plots show the relationship between log10-transformed copy number per cell values reported in the literature [33] and log10-transformed RPM of sncRNAs in combined TGIRT-seq datasets for each of the 4 cellular RNA samples. The linear regression was plotted as a light blue line with 95% confidence intervals shown as dashed blue lines. sncRNAs used in the linear regression were major spliceosomal snRNAs U1, U2, U4, U5, and U6; minor spliceosomal snRNAs U4ATAC and U6ATAC; U7, 7SL and 7SK RNA; MRP and RNase P RNA (S4 Table)[33]. Pearson (r) correlation coefficients are shown in the upper left of each panel. The Table below the plots shows literature values for three sncRNAs used as standards for droplet digital PCR (ddPCR) and copy number per cell values for these RNAs in different cellular RNA samples based on the lrm. (B) Comparison of abundance estimates for FLEXI RNAs based on the TGIRT-seq lrm and droplet digital PCR (ddPCR). The left-hand columns (TGIRT-seq) show RPM and copy number per cell values of FLEXIs estimated using the TGIRT-seq lrm for the cellular RNA samples. The right-hand columns (ddPCR) show copy number per cell values based on ddPCR abundance relative to 3 different sncRNA standards run in parallel (U7, SNORD14B, and SNORD44). For ddPCR, reverse transcription was performed on DNase-treated, size-selected (≤200 nt) RNA preparations using Maxima H reverse transcriptase with a gene-specific primer at the 3’ end of the putative FLEXI or sncRNA (S9 Table). ddPCR was then performed using primer sets within the FLEXI sequence or spanning the 5’-exon-FLEXI junction, with the latter subtracted from the FLEXI signal when applicable (S9 Table). Copy number per cell values for FLEXIs were calculated based on copy number per cell values for U7, SNORD14B, and SNORD44 in each cellular [file pgen.1011416.s006.pdf]

A

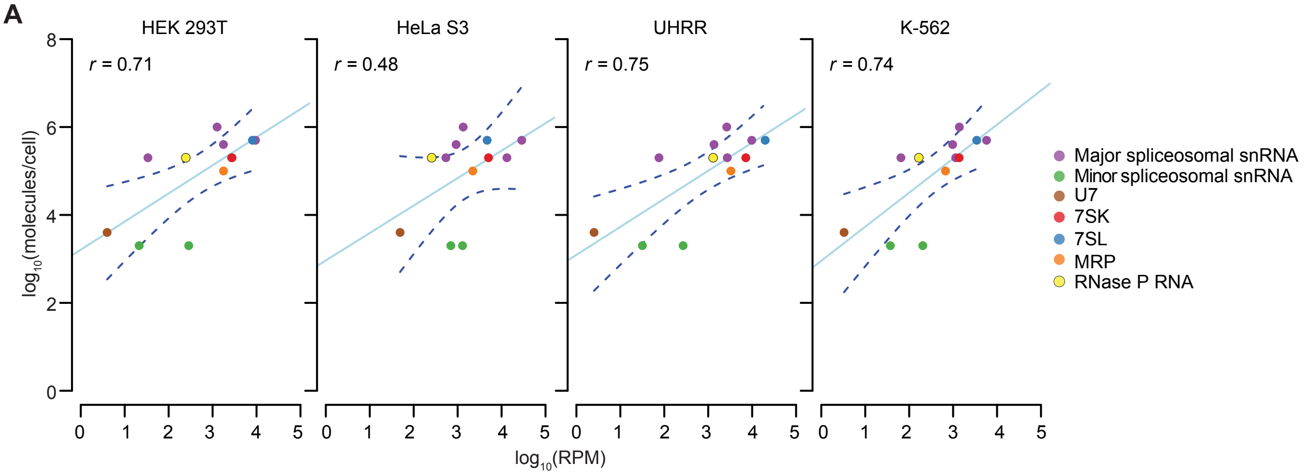

Copies/cell	Literature value	TGIRT-seq (*lrm*)		
		HEK-293T	HeLa S3	UHRR
U7	4,000	3,918	10,459	2,225
SNORD14B	10,000	19,026	69,469	15,746
SNORD44	10,000	18,739	57,072	27.924

B

HEK 293T

FLEXIs	TGIRT-seq		ddPCR		
	RPM	*lrm* Copies/cell	U7 Copies/cell	SNORD14B Copies/cell	SNORD44 Copies/cell
1I_H3F3B	0.03	180	15	1	1
3I_RAN	0.08	325	254	21	21
7I_EIF4A1	0.49	1,033	1,296	106	109
4I_JUP*	0.02	131	1,335	78	68
16I_POLG	0.24	644	25	2	2
2I_ACTB	1.32	1,933	3,393	278	285
1I_RPS2	0.47	1,001	–	–	–

Hela S3

FLEXIs	RPM	*lrm* Copies/cell	U7 Copies/cell	SNORD14B Copies/cell	SNORD44 Copies/cell
1I_H3F3B	–	–	73	9	6
3I_RAN	0.09	203	401	50	33
7I_EIF4A1	0.28	413	1,686	210	140
4I_JUP*	4.90	2,483	3,007	415	284
16I_POLG	0.19	327	–	–	–
2I_ACTB	2.11	1,468	907	113	75
1I_RPS2	0.21	346	4,310	538	358

UHRR

FLEXIs	RPM	*lrm* Copies/cell	U7 Copies/cell	SNORD14B Copies/cell	SNORD44 Copies/cell
1I_H3F3B	0.05	185	180	76	12
3I_RAN	0.06	195	186	78	12
7I_EIF4A1	0.29	565	230	97	15
4I_JUP*	0.03	127	460	158	25
16I_POLG	0.25	504	–	–	–
2I_ACTB	6.90	4,232	1,768	746	118
1I_RPS2	0.18	411	3,833	1,618	257

-: not detected
*: annotated agotron

S6 Fig. Estimates of FLEXI RNA abundance using a linear regression model and droplet digital PCR.
(A) Linear regression models (*lrm*) of molecule per cell values based on RPM values in TGIRT-seq datasets of sncRNAs with literature reported copy number per cell values. The scatter plots show the relationship between log_{10}-transformed copy number per cell values reported in the literature (33) and log_{10}-transformed RPM of sncRNAs in combined TGIRT-seq datasets for each of the 4 cellular RNA samples. The linear regression was plotted as a light blue line with 95% confidence intervals shown as dashed blue lines. sncRNAs used in the linear regression were major spliceosomal snRNAs U1, U2, U4, U5, and U6; minor spliceosomal snRNAs U4ATAC and U6ATAC; U7, 7SL and 7SK RNA; MRP and RNase P RNA (S4 Table)(33). Pearson (r) correlation coefficients are shown in the upper left of each panel. The Table below the plots shows literature values for three sncRNAs used as standards for droplet digital PCR (ddPCR) and copy number per cell values for these RNAs in different cellular RNA samples based on the lrm. **(B)** Comparison of abundance estimates for FLEXI RNAs based on the TGIRT-seq *lrm* and droplet digital PCR (ddPCR). The left-hand columns (TGIRT-seq) show RPM and copy number per cell values of FLEXIs estimated using the TGIRT-seq *lrm* for the cellular RNA samples. The right-hand columns (ddPCR) show copy number per cell values based on ddPCR abundance relative to 3 different sncRNA standards run in parallel (U7, SNORD14B, and SNORD44). For ddPCR, reverse transcription was performed on DNase-treated, size-selected (≤200 nt) RNA preparations using Maxima H reverse transcriptase with a gene-specific primer at the 3' end of the putative FLEXI or sncRNA (S9 Table). ddPCR was then performed using primer sets within the FLEXI sequence or spanning the 5'-exon-FLEXI junction, with the latter subtracted from the FLEXI signal when applicable (S9 Table). Copy number per cell values for FLEXIs were calculated based on copy number per cell values for U7, SNORD14B, and SNORD44 in each cellular RNA sample obtained using the *lrm* (Table at the bottom of panel A).
