# Supplementary material for: Human cells contain myriad excised linear intron RNAs with links to gene regulation and potential utility as biomarkers
Source: PLoS Genet. 2024 Sep 26;20(9):e1011416. doi: 10.1371/journal.pgen.1011416 (PMC11460701; doi:10.1371/journal.pgen.1011416)
Supplement: S7 Fig — (A) and (B) PCA-initialized t-SNE [85] and ZINB-WaVE [34] analysis of FLEXI RNAs detected at ≥1 read (panel A) or ≥0.01 RPM (panel B) in biological and technical replicates of TGIRT-seq for rRNA-depleted unfragmented RNAs from different cellular RNA samples (S1 Table). t-SNE and ZINB-WaVE are widely used for the analysis of single cell RNA-seq datasets with zero-inflated counts. The top row of each panel shows clustering of datasets for technical replicates for different cell types used in this study. The middle and bottom rows of each panel show clustering of datasets for biological replaces of rRNA-depleted unfragmented HEK-293T, K-562, MDA-MB-231, and UHRR RNAs from this study (biological replicate 1 (Bio 1, circles) and other studies (biological replicate 2 (Bio 2), triangles) before (middle) and after (bottom) batch effect correction by ZINB-WaVE. The compared datasets are listed in S1 Table. (PDF) [file pgen.1011416.s007.pdf]

**A All FLEXIs (≥1 read)**

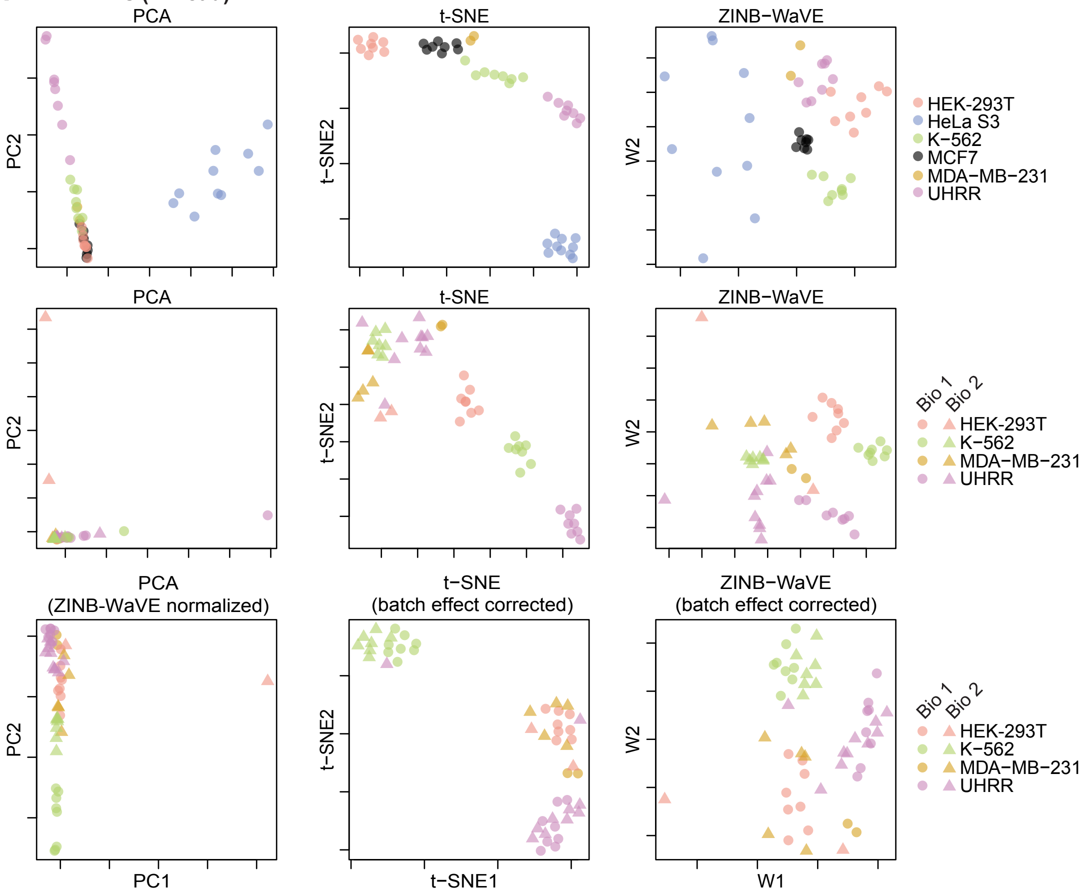


**B Abundant FLEXIs (≥0.01 RPM)**

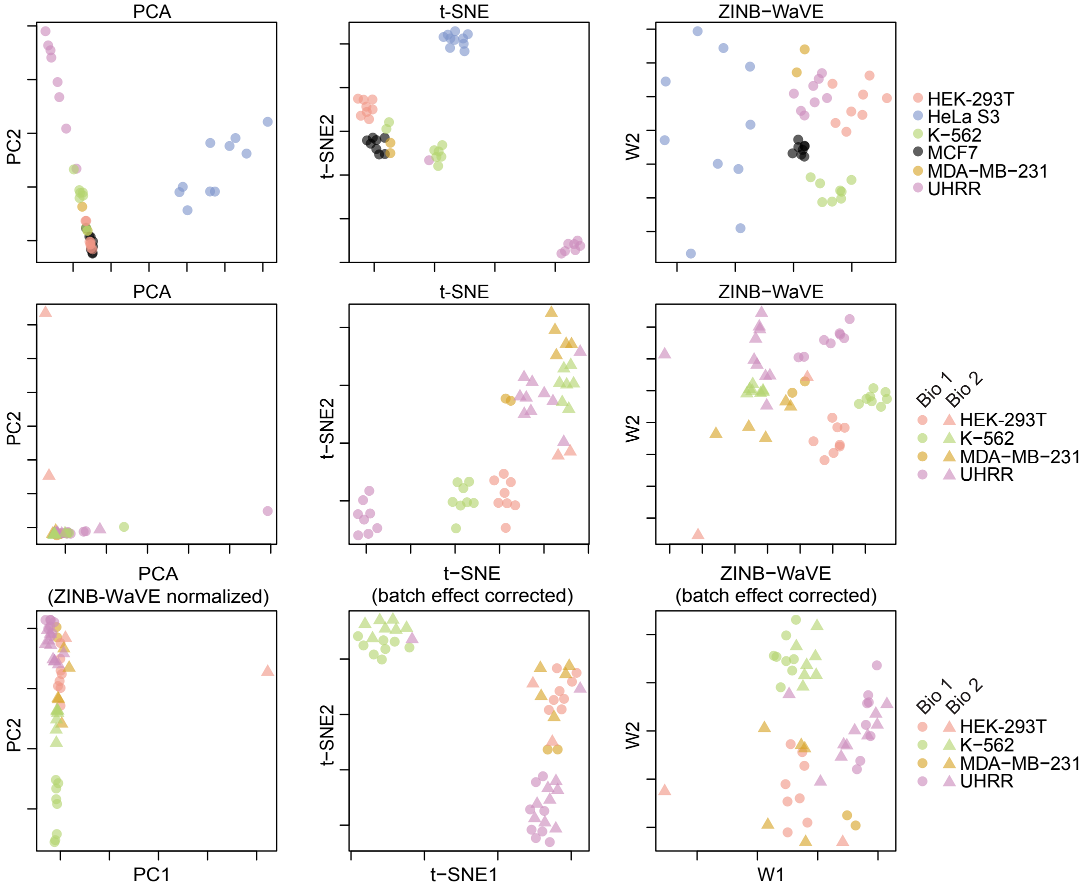

**S7 Fig. Reproducibility of cell-type specific expression patterns of FLEXIs.**
**(A)** and **(B)** PCA-initialized t-SNE (77) and ZINB-WaVE (34) analysis of FLEXI RNAs detected at ≥1 read (panel A) or ≥0.01 RPM (panel B) in biological and technical replicates of TGIRT-seq for rRNA-depleted unfragmented RNAs from different cellular RNA samples (S1 Table). t-SNE and ZINB-WaVE are widely used for the analysis of single cell RNA-seq datasets with zero-inflated counts. The top row of each panel shows clustering of datasets for technical replicates for different cell types used in this study. The middle and bottom rows of each panel show clustering of datasets for biological replaces of rRNA-depleted unfragmented HEK-293T, K-562, MDA-MB-231, and UHRR RNAs from this study (biological replicate 1 (Bio 1, circles) and other studies (biological replicate 2 (Bio 2), triangles) before (middle) and after (bottom) batch effect correction by ZINB-WaVE. The compared datasets are listed in S1 Table.
