# Supplementary material for: Human cells contain myriad excised linear intron RNAs with links to gene regulation and potential utility as biomarkers
Source: PLoS Genet. 2024 Sep 26;20(9):e1011416. doi: 10.1371/journal.pgen.1011416 (PMC11460701; doi:10.1371/journal.pgen.1011416)
Supplement: S8 Fig — (A) IGV screenshots showing examples of differences in abundance of FLEXI RNAs transcribed from the same host gene reflecting post-transcriptional differences in splicing efficiency, alternative splicing, or differential stability of FLEXI RNAs. Gene maps for different RNA isoforms generated by alternative splicing of FLEXI RNAs are shown at the top and bottom with introns from the 5’ to 3’ RNA end named A to F. Reads mapping to exons or non-FLEXI introns were omitted for clarity. Reads for FLEXIs from different cellular RNA samples are color coded as shown in the Figure. NTA, non-templated nucleotides added to the 3’ end of cDNAs during TGIRT-seq library preparation. Two FLEXIs in the top panel had indels at short runs of 3’ U residues, likely reflecting a previously reported tendency for TGIRT enzyme slippage at such locations [86]. (B) IGV screen shots showing differences in expression levels for long genes with labels omitted for better visualization of multiple FLEXIs from the same host gene. (PDF) [file pgen.1011416.s008.pdf]

A

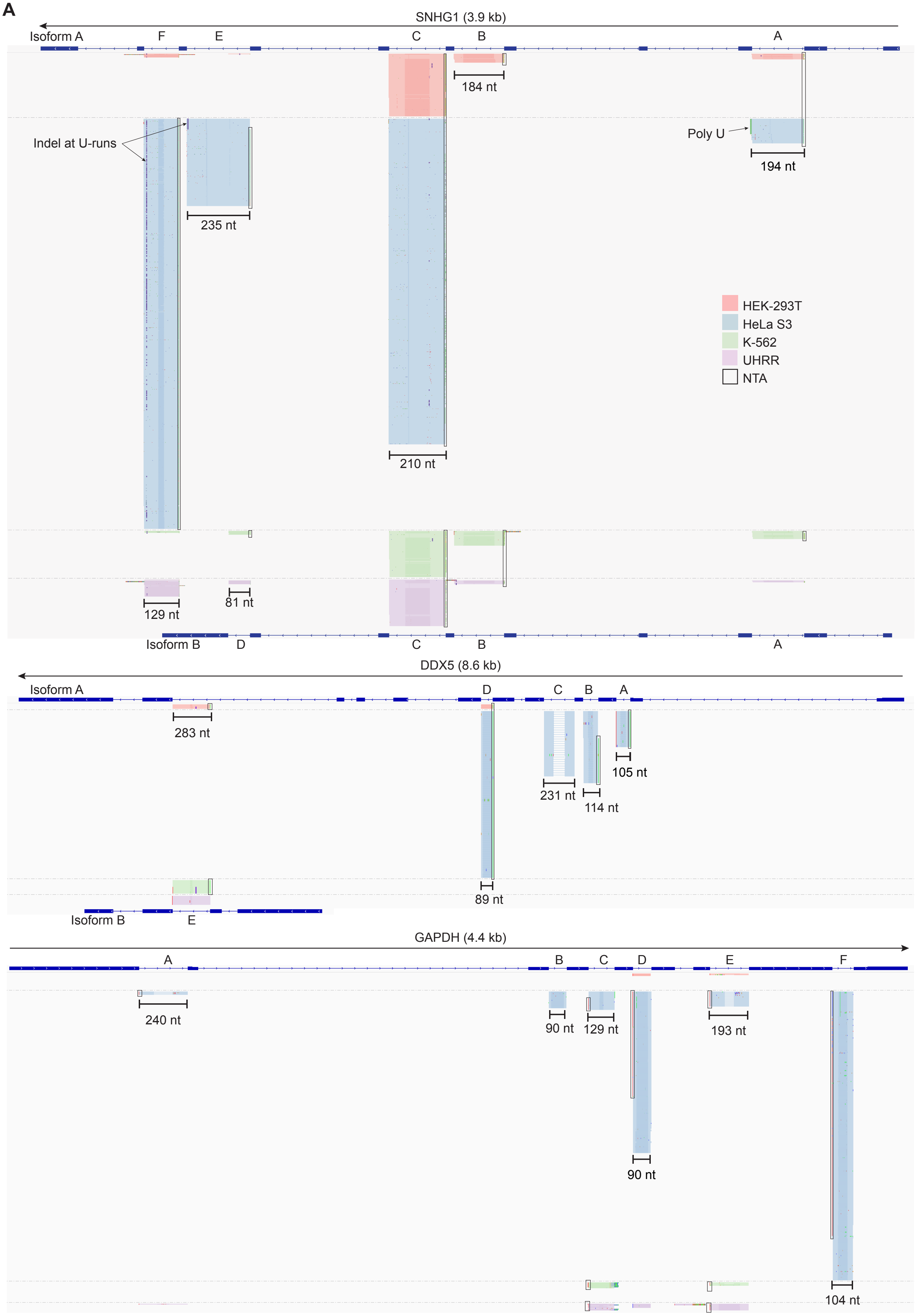
SNHG1 (3.9 kb)
Isoform A
F
E
C
B
A
184 nt
Indel at U-runs
Poly U
194 nt
235 nt
HEK-293T
HeLa S3
K-562
UHRR
NTA
210 nt
129 nt
81 nt
Isoform B
D
C
B
A
DDX5 (8.6 kb)
Isoform A
D
C
B
A
283 nt
105 nt
231 nt
114 nt
89 nt
Isoform B
E
GAPDH (4.4 kb)
A
B
C
D
E
F
240 nt
90 nt
129 nt
193 nt
90 nt
104 nt

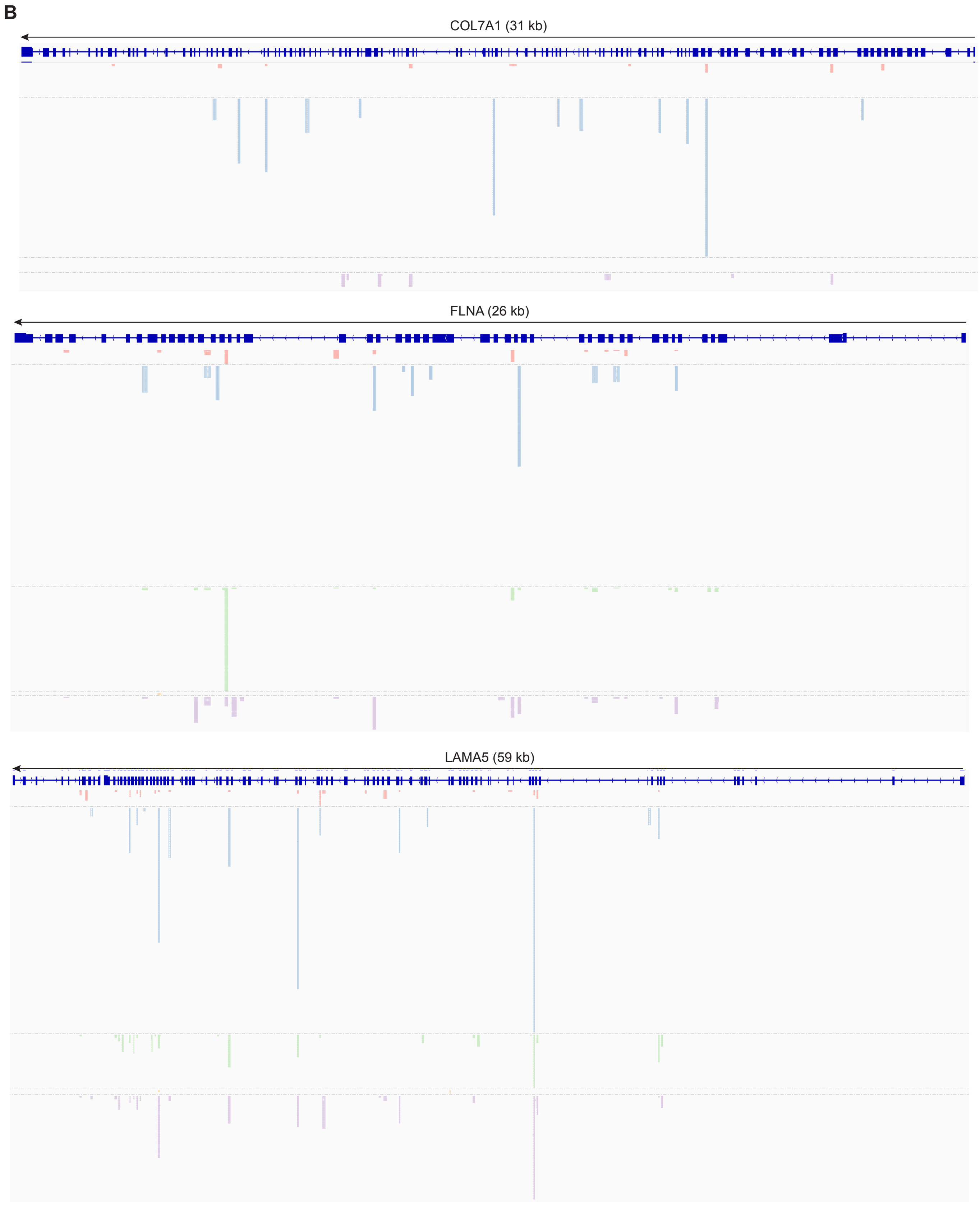
B
COL7A1 (31 kb)
FLNA (26 kb)
LAMA5 (59 kb)

S8 Fig. FLEXI RNAs from the same host gene can differ widely in expression levels.
(A) IGV screenshots showing examples of differences in abundance of FLEXI RNAs transcribed from the same host gene reflecting post-transcriptional differences in splicing efficiency, alternative splicing, or differential stability of FLEXI RNAs. Gene maps for different RNA isoforms generated by alternative splicing of FLEXI RNAs are shown at the top and bottom with introns from the 5' to 3' RNA end named A to F. Reads mapping to exons or non-FLEXI introns were omitted for clarity. Reads for FLEXIs from different cellular RNA samples are color coded as shown in the Figure. NTA, non-templated nucleotides added to the 3' end of cDNAs during TGIRT-seq library preparation. Two FLEXIs in the top panel had indels at short runs of 3' U residues, likely reflecting a previously reported tendency for TGIRT enzyme slippage at such locations (78). **(B)** IGV screen shots showing differences in expression levels for long genes with labels omitted for better visualization of multiple FLEXIs from the same host gene.
