# Supplementary material for: Human cells contain myriad excised linear intron RNAs with links to gene regulation and potential utility as biomarkers
Source: PLoS Genet. 2024 Sep 26;20(9):e1011416. doi: 10.1371/journal.pgen.1011416 (PMC11460701; doi:10.1371/journal.pgen.1011416)
Supplement: S9 Fig — (A) Heatmap comparing the abundance of 200 FLEXI RNAs in biological replicates (1 and 2, top) and technical replicates (separated by black vertical lines) of TGIRT-seq datasets for rRNA-depleted unfragmented cellular RNA samples (S1 Table). FLEXI abundance was color coded by log2-transformed RPM values. Clusters of FLEXIs that were highly expressed in different cell lines are highlighted in boxes with yellow borders. (B) Box plots showing examples of the relative abundance of cell-type specific FLEXIs in the TGIRT-seq datasets of panel A. UHRR, which is comprised of RNAs from multiple human cell lines, was omitted from this panel. (PDF) [file pgen.1011416.s009.pdf]

A

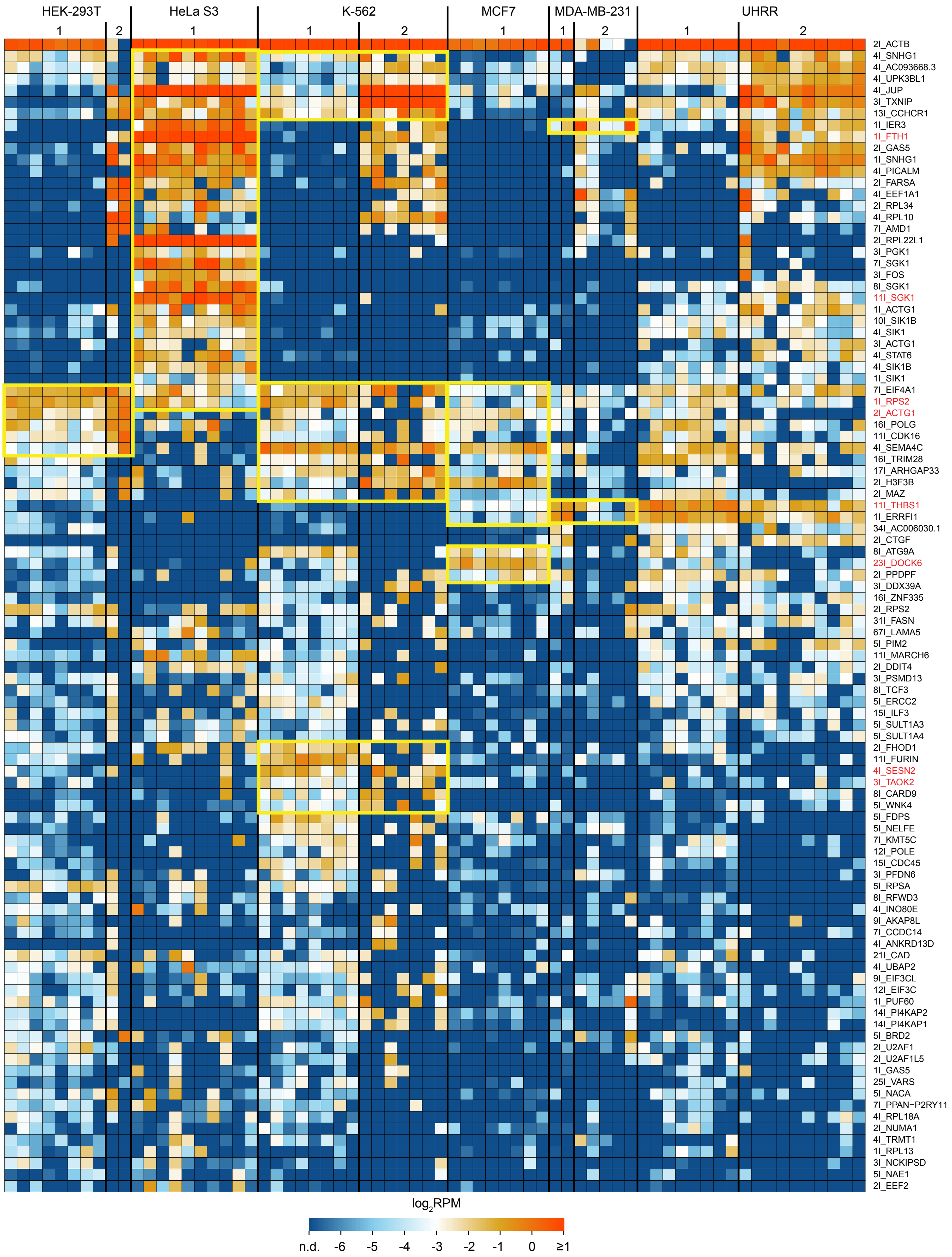

HEK-293T
HeLa S3
K-562
MCF7
MDA-MB-231
UHRR
1
2
1
1
2
1
1
2
1
2
2l_ACTB
4l_SNHG1
4l_AC093668.3
4l_UPK3BL1
4l_JUP
3l_TXNIP
13l_CCHCR1
1l_IER3
1l_FTH1
2l_GAS5
1l_SNHG1
4l_PICALM
2l_FARSA
4l_EEF1A1
2l_RPL34
4l_RPL10
7l_AMD1
2l_RPL22L1
3l_PGK1
7l_SGK1
3l_FOS
8l_SGK1
11l_SGK1
1l_ACTG1
10l_SIK1B
4l_SIK1
3l_ACTG1
4l_STAT6
4l_SIK1B
1l_SIK1
7l_EIF4A1
1l_RPS2
2l_ACTG1
16l_POLG
11l_CDK16
4l_SEMA4C
16l_TRIM28
17l_ARHGAP33
2l_H3F3B
2l_MAZ
11l_THBS1
1l_ERRFI1
34l_AC006030.1
2l_CTGF
8l_ATG9A
23l_DOCK6
2l_PPDPF
3l_DDX39A
16l_ZNF335
2l_RPS2
31l_FASN
67l_LAMA5
5l_PIM2
11l_MARCH6
2l_DDIT4
3l_PSMD13
8l_TCF3
5l_ERCC2
15l_ILF3
5l_SULT1A3
5l_SULT1A4
2l_FHOD1
11l_FURIN
4l_SESN2
3l_TAOK2
8l_CARD9
5l_WNK4
5l_FDPS
5l_NELFE
7l_KMT5C
12l_POLE
15l_CDC45
3l_PFDN6
5l_RPSA
8l_RFWD3
4l_INO80E
9l_AKAP8L
7l_CCDC14
4l_ANKRD13D
21l_CAD
4l_UBAP2
9l_EIF3CL
12l_EIF3C
1l_PUF60
14l_PI4KAP2
14l_PI4KAP1
5l_BRD2
2l_U2AF1
2l_U2AF1L5
1l_GAS5
25l_VARS
5l_NACA
7l_PPAN-P2RY11
4l_RPL18A
2l_NUMA1
4l_TRMT1
1l_RPL13
3l_NCKIPSD
5l_NAE1
2l_EEF2
log_2RPM
n.d.
-6
-5
-4
-3
-2
-1
0
≥1

B

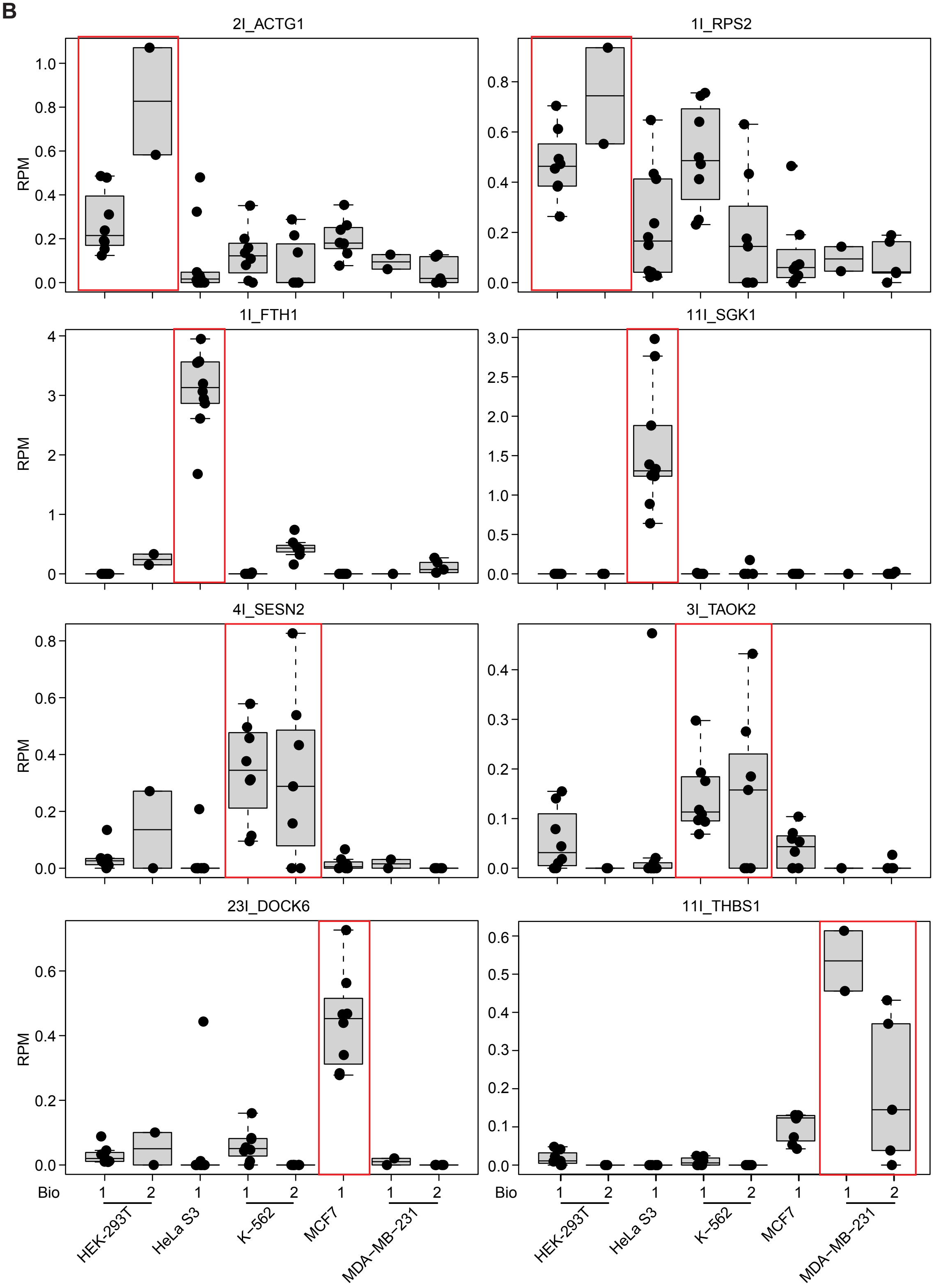

S9 Fig. Heat map and box plots comparing the relative abundance of FLEXIs in TGIRT-seq biological and technical replicates of cellular RNA samples.

(A) Heatmap comparing the abundance of 200 FLEXI RNAs in biological replicates (1 and 2, top) and technical replicates (separated by black vertical lines) of TGIRT-seq datasets for rRNA-depleted unfragmented cellular RNA samples (S1 Table). FLEXI abundance was color coded by log_2-transformed RPM values. Clusters of FLEXIs that were highly expressed in different cell lines are highlighted in boxes with yellow borders. **(B)** Box plots showing examples of the relative abundance of cell-type specific FLEXIs in the TGIRT-seq datasets of panel A. UHRR, which is comprised of RNAs from multiple human cell lines, was omitted from this panel.
