# Supplementary material for: Human cells contain myriad excised linear intron RNAs with links to gene regulation and potential utility as biomarkers
Source: PLoS Genet. 2024 Sep 26;20(9):e1011416. doi: 10.1371/journal.pgen.1011416 (PMC11460701; doi:10.1371/journal.pgen.1011416)
Supplement: S10 Fig — Bar graphs showing the number of (A) FLEXI RNAs, (B) Other Ensembl GRCh38-annotated short introns (≤300 nt), and (C) Ensembl GRCh38-annotated long introns (>300 nt) that have a CLIP-seq-identified binding site for the indicated RBP in a merged dataset for the K-562, HEK-293T, HeLa S3, and UHRR cellular RNA samples. Bars graphs are color coded by RBP function as shown at the top. Asterisks above the bars in panels B and C indicate the 53 proteins identified as binding ≥30 different FLEXIs in Fig 4A. (PDF) [file pgen.1011416.s010.pdf]

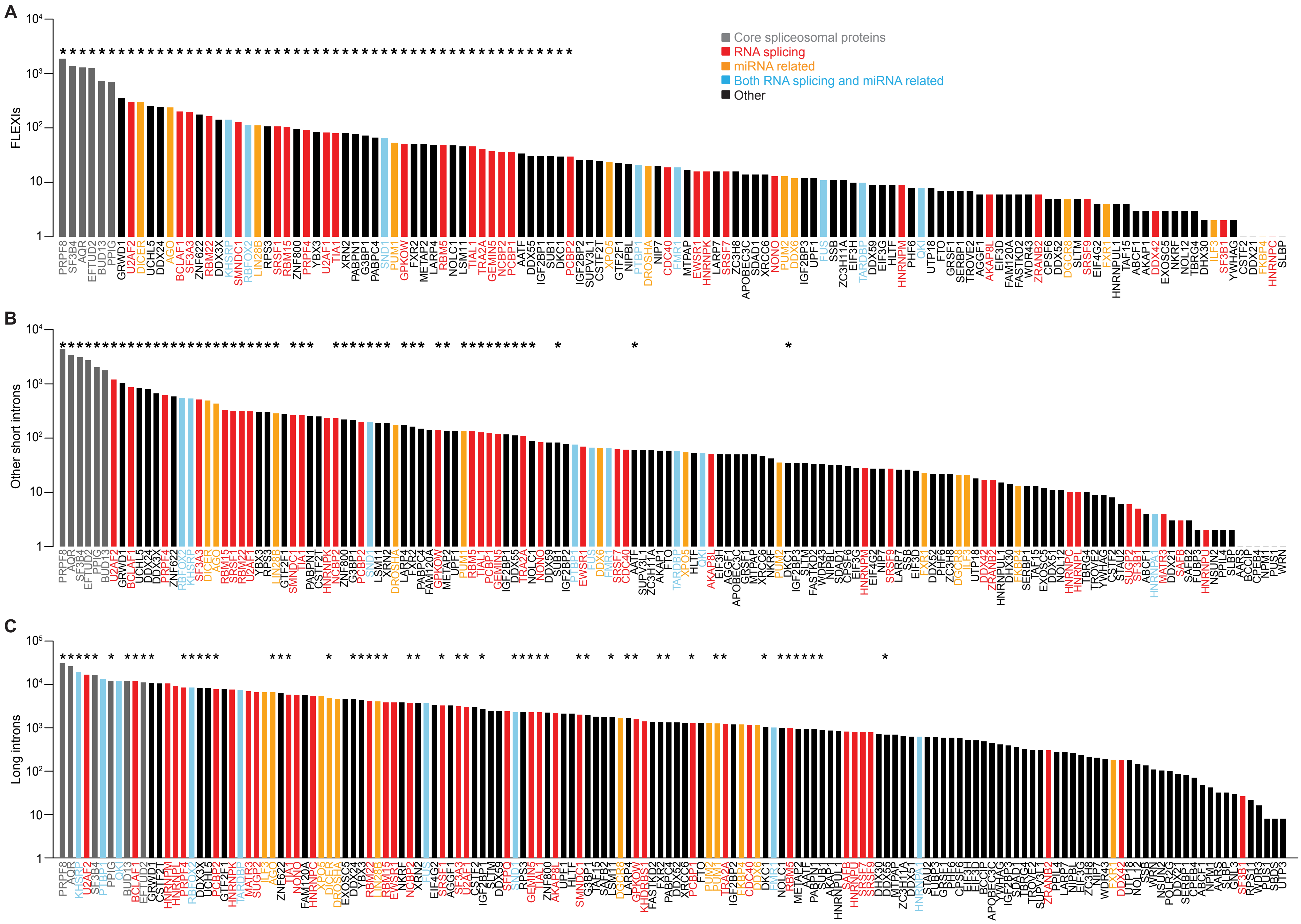

S10 Fig. Quantitation of RBP-binding sites in different classes of intron RNAs.

Bar graphs showing the number of **(A)** FLEXI RNAs, **(B)** Other Ensembl GRCh38-annotated short introns (≤300 nt), and **(C)** Ensembl GRCh38-annotated long introns (>300 nt) that have a CLIP-seq-identified binding site for the indicated RBP in a merged dataset for the K-562, HEK-293T, HeLa S3, and UHRR cellular RNA samples. Bars graphs are color coded by RBP function as shown at the top. Asterisks above the bars in panels B and C indicate the 53 proteins identified as binding ≥30 different FLEXIs in Fig 4A.
