# Supplementary material for: Human cells contain myriad excised linear intron RNAs with links to gene regulation and potential utility as biomarkers
Source: PLoS Genet. 2024 Sep 26;20(9):e1011416. doi: 10.1371/journal.pgen.1011416 (PMC11460701; doi:10.1371/journal.pgen.1011416)
Supplement: S11 Fig — Density plots of ENCODE eCLIP-identified RBP-binding sites in K-562 or Hep G2 cells and PAR-CLIP identified binding sites for AGO1-4 and DICER in HEK-293 cells [38–40] for the 53 RBPs with binding sites for ≥30 FLEXIs (≤300 nt; red line) compared to other short introns (≤300 nt; blue line) and long introns (>300 nt; back lines). RBP-binding sites overlapping introns (≥1 nt) were identified by intersecting annotated RBP-binding sites with intron coordinates using BEDTools and plotted as the mid-point of the annotated RBP-binding sites normalized as a percentage of intron length with 0% and 100% corresponding to the 5’ and 3’ end of the intron, respectively. Vertical red dashed lines indicate the position of peaks in the density plots for FLEXI RNAs. RBP names are color coded by protein function as indicated at the bottom of the Figure. Blank spaces were left for datasets that were not available for one of the two cell lines used to obtain the eCLIP datasets. (PDF) [file pgen.1011416.s011.pdf]

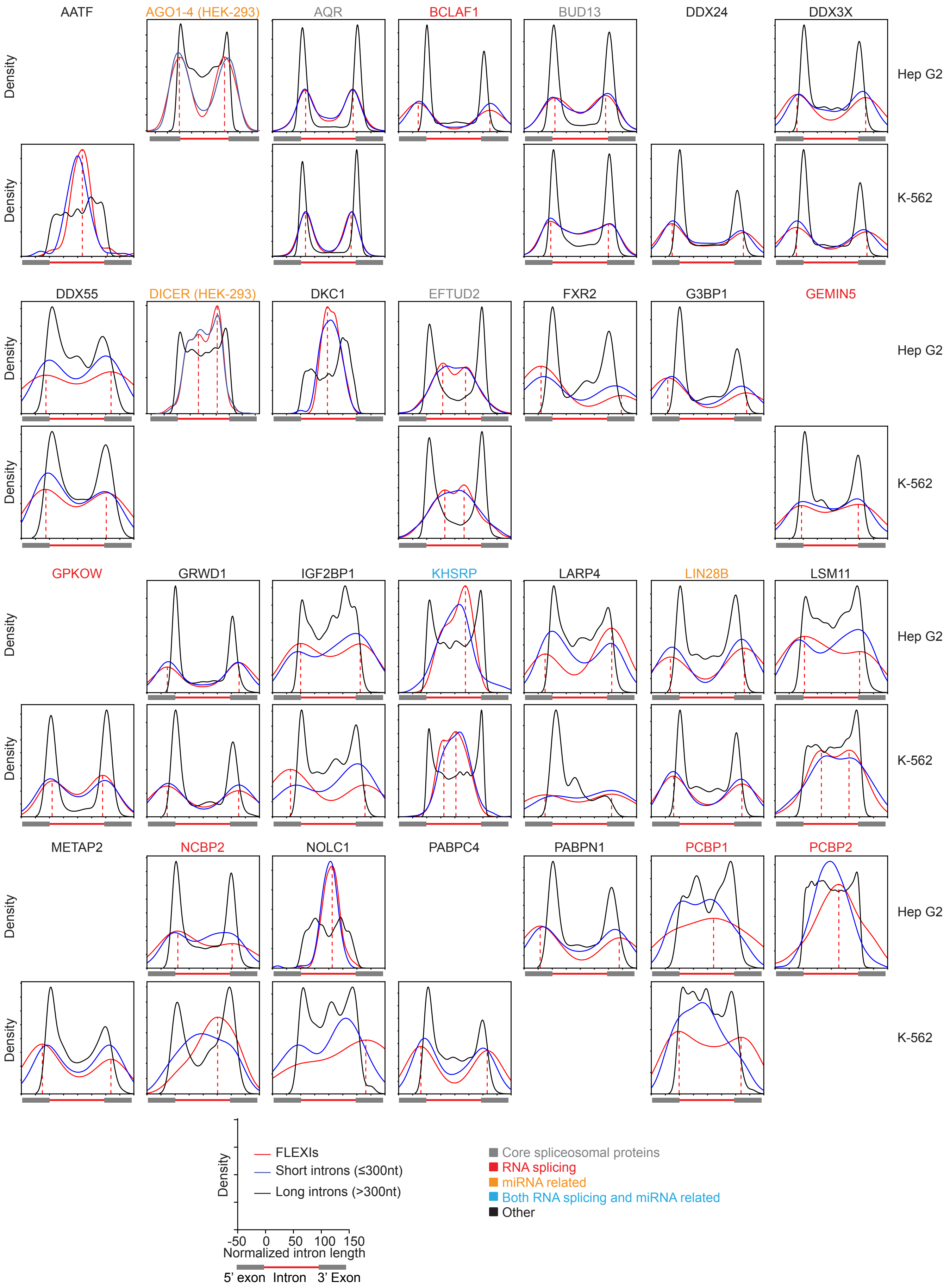
AATF
AGO1-4 (HEK-293)
AQR
BCLAF1
BUD13
DDX24
DDX3X
Hep G2
K-562
Density
DDX55
DICER (HEK-293)
DKC1
EFTUD2
FXR2
G3BP1
GEMIN5
GPKOW
GRWD1
IGF2BP1
KHSRP
LARP4
LIN28B
LSM11
METAP2
NCBP2
NOLC1
PABPC4
PABPN1
PCBP1
PCBP2
FLEXIs
Short introns (≤300nt)
Long introns (>300nt)
-50 0 50 100 150
Normalized intron length
5' exon Intron 3' Exon
Core spliceosomal proteins
RNA splicing
miRNA related
Both RNA splicing and miRNA related
Other

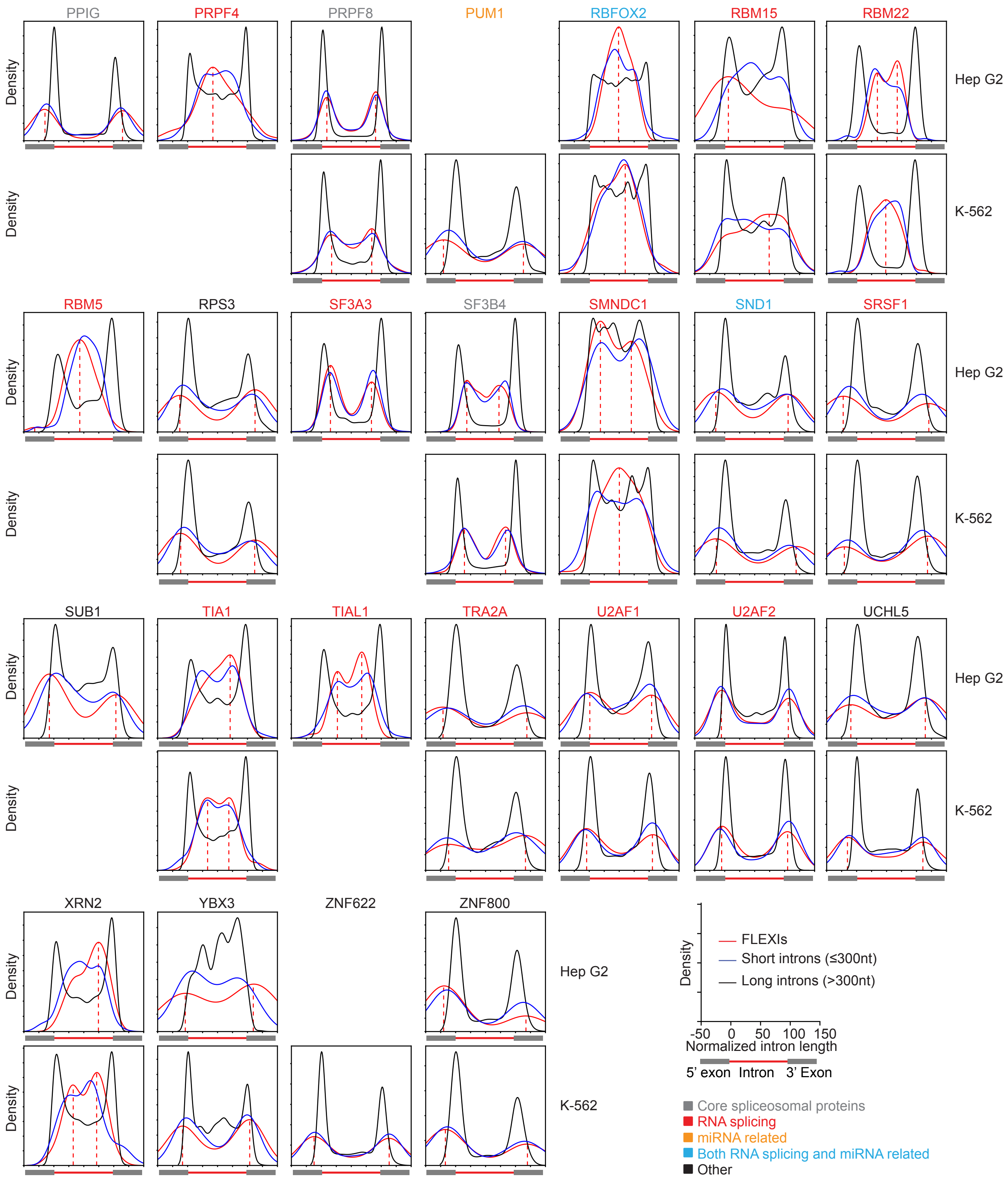

S11 Fig. Locations of RBP-binding sites in FLEXIs and other classes of introns.
Density plots of ENCODE eCLIP-identified RBP-binding sites in K-562 or Hep G2 cells and PAR-CLIP identified binding sites for AGO1-4 and DICER in HEK-293 cells (38-40) for the 53 RBPs with binding sites for ≥30 FLEXIs (≤300 nt; red line) compared to other short introns (≤300 nt; blue line) and long introns (>300 nt; back lines). RBP-binding sites overlapping introns (≥1 nt) were identified by intersecting annotated RBP-binding sites with intron coordinates using BEDTools and plotted as the mid-point of the annotated RBP-binding sites normalized as a percentage of intron length with 0% and 100% corresponding to the 5' and 3' end of the intron, respectively. Vertical red dashed lines indicate the position of peaks in the density plots for FLEXI RNAs. RBP names are color coded by protein function as indicated at the bottom of the Figure. Blank spaces were left for datasets that were not available for one of the two cell lines used to obtain the eCLIP datasets.
