# Supplementary material for: Human cells contain myriad excised linear intron RNAs with links to gene regulation and potential utility as biomarkers
Source: PLoS Genet. 2024 Sep 26;20(9):e1011416. doi: 10.1371/journal.pgen.1011416 (PMC11460701; doi:10.1371/journal.pgen.1011416)
Supplement: S12 Fig — eCLIP datasets for Hep G2 and K-562 cells were downloaded from ENCODE for all 51 RBPs with binding sites for ≥30 FLEXIs, and reads in those datasets that were ≥95% of the length of a FLEXI were identified by intersecting the eCLIP reads with FLEXI coordinates. Data are shown as bar graphs for fold enrichment of ≥95% full-length intron reads for each RBP in the eCLIP dataset versus the control antibody dataset. The dashed line indicates 2-fold enrichment. Names of proteins are color coded by protein function as indicated below the bar graph. For those RBPs in which more than one dataset was available, mean values were calculated for the fold enrichment in eCLIP replicates over controls. Only those RBPs that have ≥2-fold enrichment are shown (S5 Table). (PDF) [file pgen.1011416.s012.pdf]

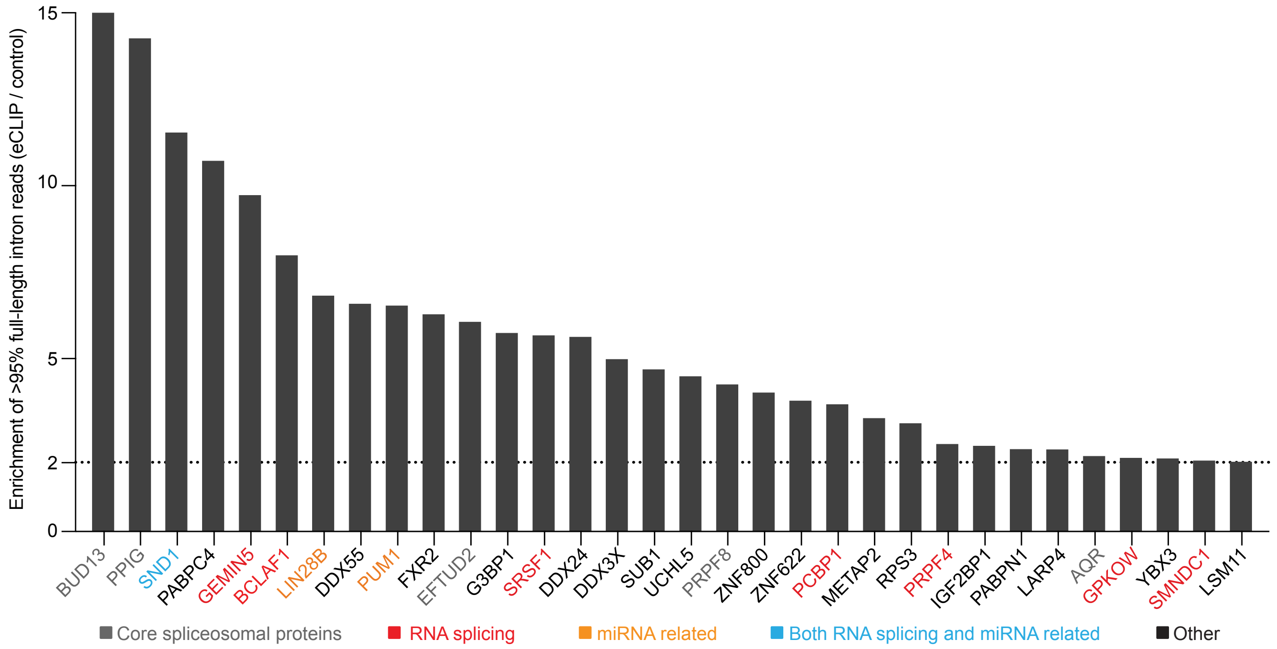

S12 Fig. Enrichment of sequencing reads corresponding to near full-length FLEXIs found in ENCODE eCLIP datasets despite RNase treatment used in processing cross-linked samples.
eCLIP datasets for Hep G2 and K-562 cells were downloaded from ENCODE for all 51 RBPs with binding sites for ≥30 FLEXIs, and reads in those datasets that were ≥95% of the length of a FLEXI were identified by intersecting the eCLIP reads with FLEXI coordinates. Data are shown as bar graphs for fold enrichment of ≥95% full-length intron reads for each RBP in the eCLIP dataset versus the control antibody dataset. The dashed line indicates 2-fold enrichment. Names of proteins are color coded by protein function as indicated below the bar graph. For those RBPs in which more than one dataset was available, mean values were calculated for the fold enrichment in eCLIP replicates over controls. Only those RBPs that have ≥2-fold enrichment are shown (S5 Table).
