# Supplementary material for: Human cells contain myriad excised linear intron RNAs with links to gene regulation and potential utility as biomarkers
Source: PLoS Genet. 2024 Sep 26;20(9):e1011416. doi: 10.1371/journal.pgen.1011416 (PMC11460701; doi:10.1371/journal.pgen.1011416)
Supplement: S13 Fig — Volcano plots showing -log10-transformed adjusted p-values versus log2-transformed fold changes for ENSEMBL-annotated genes in ENCODE knockdown versus control datasets for the indicated RBPs in K-562 and Hep G2 cells. Host genes of (A) FLEXIs, (B) other short introns, and (C) long introns that contain an annotated binding site for the indicated RBP that have significant differential expression (DE) in mRNA levels (adjusted p≤0.05, |LFC|≥1) in the knockdown datasets are shown as red dots. Other genes with or without significant expression changes are shown as black or gray dots, respectively. RBPs whose knockdown resulted in a significant bias towards increased or decreased mRNA levels from host genes encoding FLEXIs, other short introns, and long introns with an annotated binding site for the RBP compared to genes whose transcript lack an annotated binding site for the same RBP are indicated by up (light blue) or down (red) arrows, respectively next to the RBP name. For these comparisons, significant bias is defined as p-value ≤0.05 determined by Fisher’s exact test comparing the ratio of significantly up-regulated (log2FC>0, adjusted p≤0.05) or down-regulated (log2FC<0, adjusted p≤0.05) host genes whose FLEXIs, other short introns, or long introns contain an annotated binding site for the RBP to those in all significantly changed genes whose transcripts lack an annotated binding site for the same RBP. RBP knockdowns that resulted in significant changes in mRNA levels from host genes containing a FLEXI, other short introns, and long introns with a binding site for the knocked down RBP, but no significant directional bias, are indicated by a (gray) bi-directional arrow next to the RBP name. Plots are shown only from those RBPs whose knockdown resulted in a significant difference (p≤0.05) in the number of DE genes whose FLEXIs, other short introns, or long introns contain an annotated binding site for the RBP. Datasets that were not available for an RBP in one of the [file pgen.1011416.s013.pdf]

A. FLEXIs

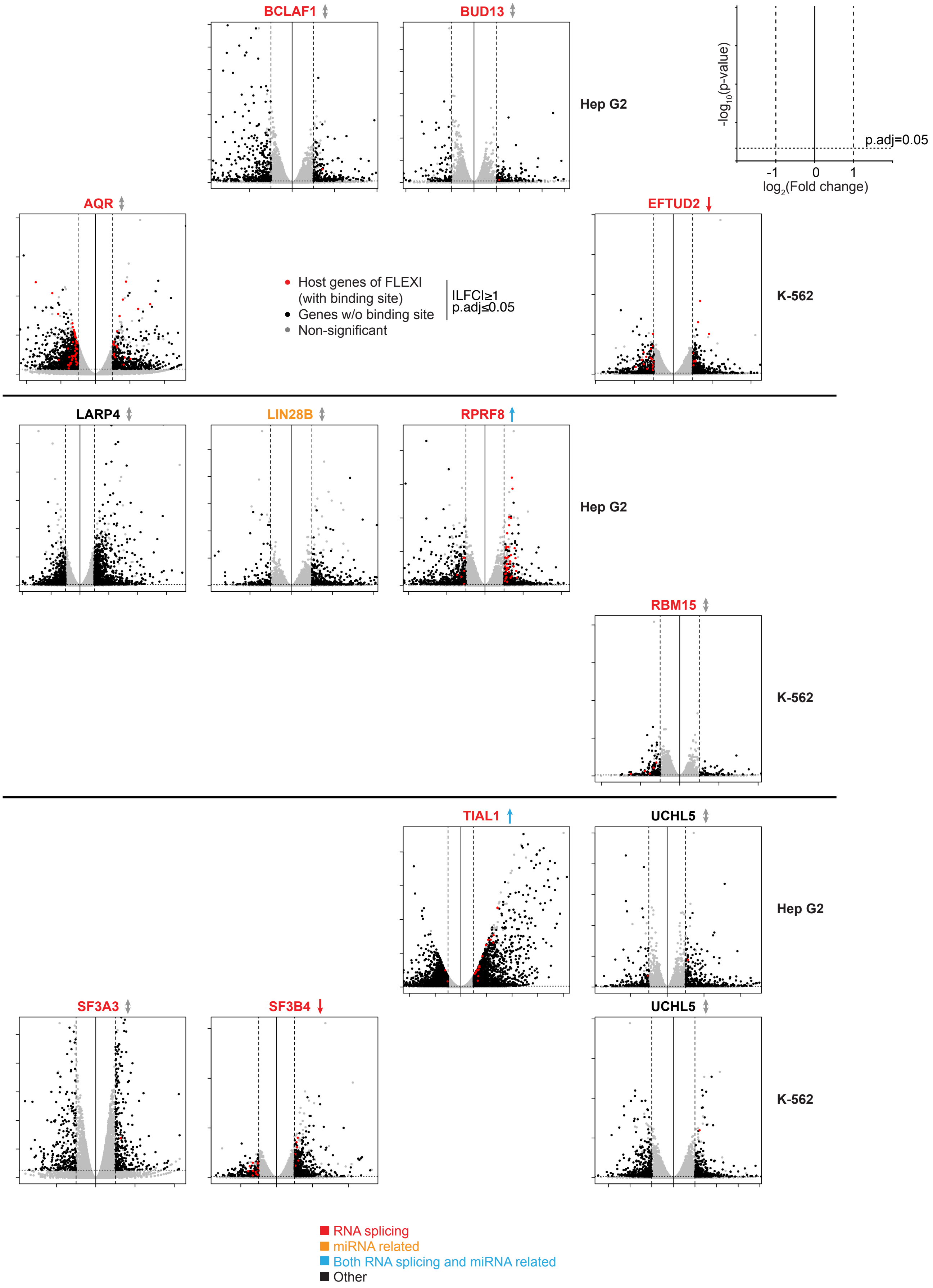

BCLAF1
BUD13
Hep G2
-log10(p-value)
p.adj=0.05
-1
0
1
log2(Fold change)
AQR
EFTUD2
Host genes of FLEXI (with binding site)
Genes w/o binding site
Non-significant
lLFCl≥1
p.adj≤0.05
K-562
LARP4
LIN28B
RPRF8
Hep G2
RBM15
K-562
TIAL1
UCHL5
Hep G2
SF3A3
SF3B4
UCHL5
K-562
RNA splicing
miRNA related
Both RNA splicing and miRNA related
Other

B. Other short introns

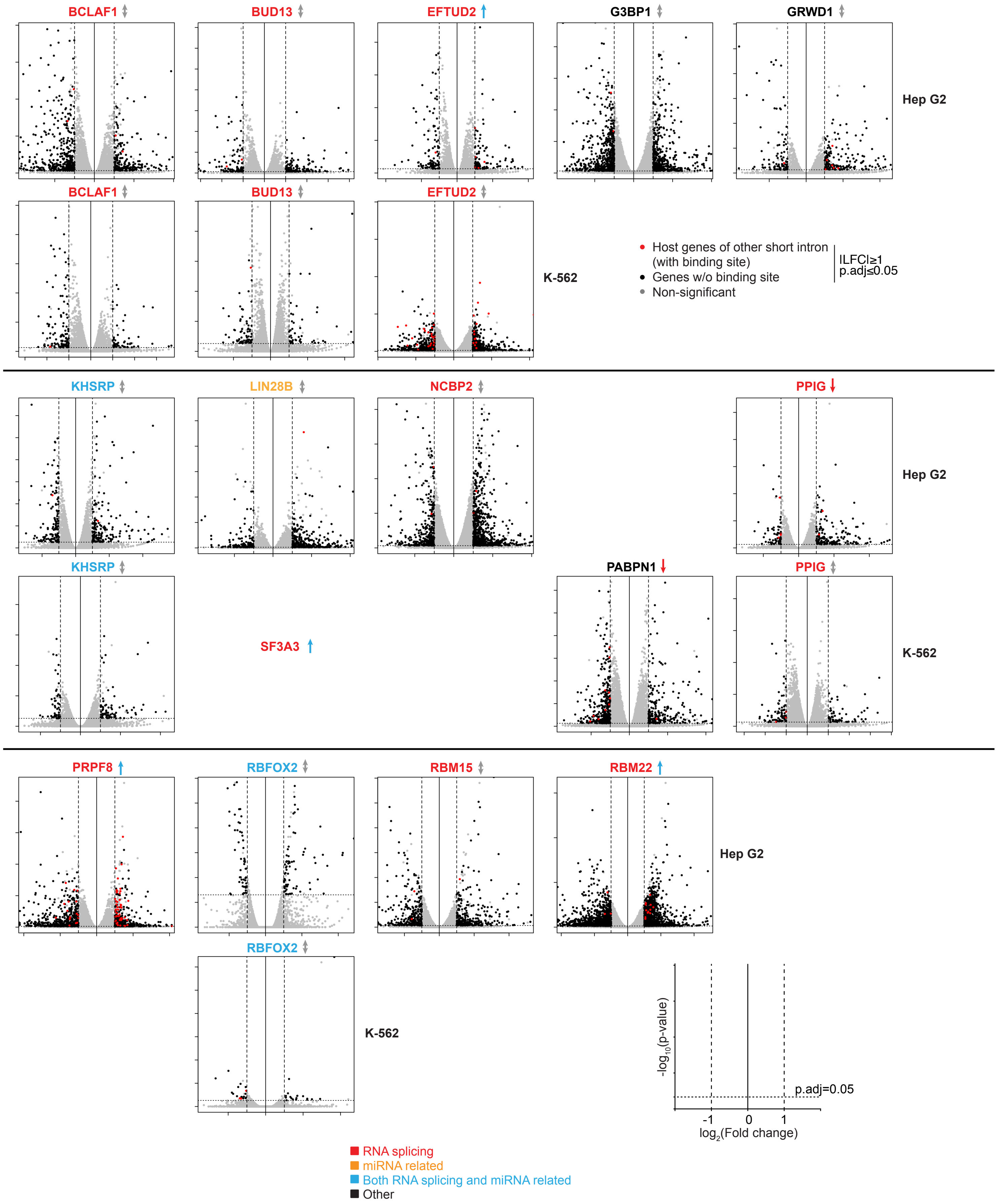

B. Other short introns

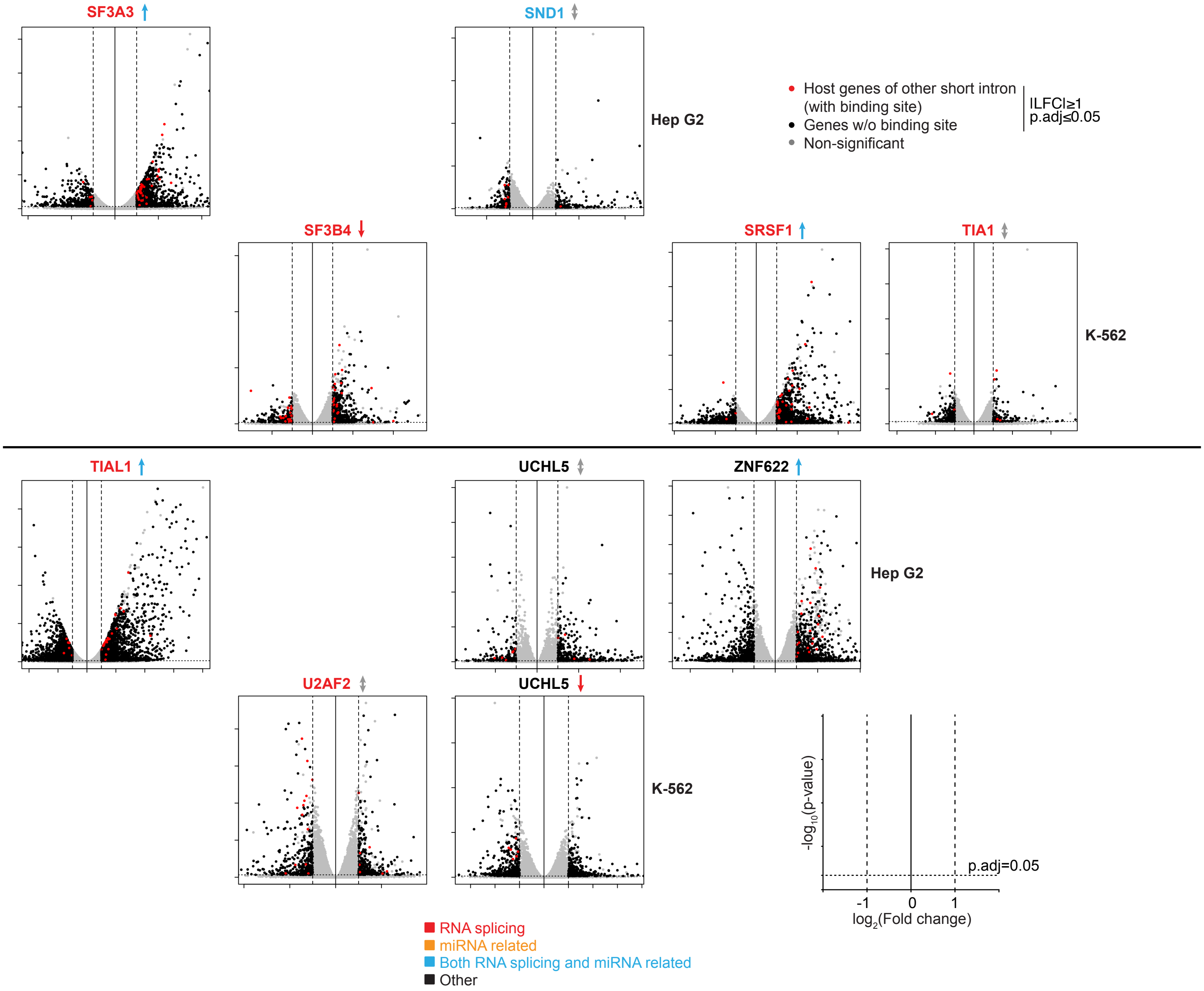

C. Long introns

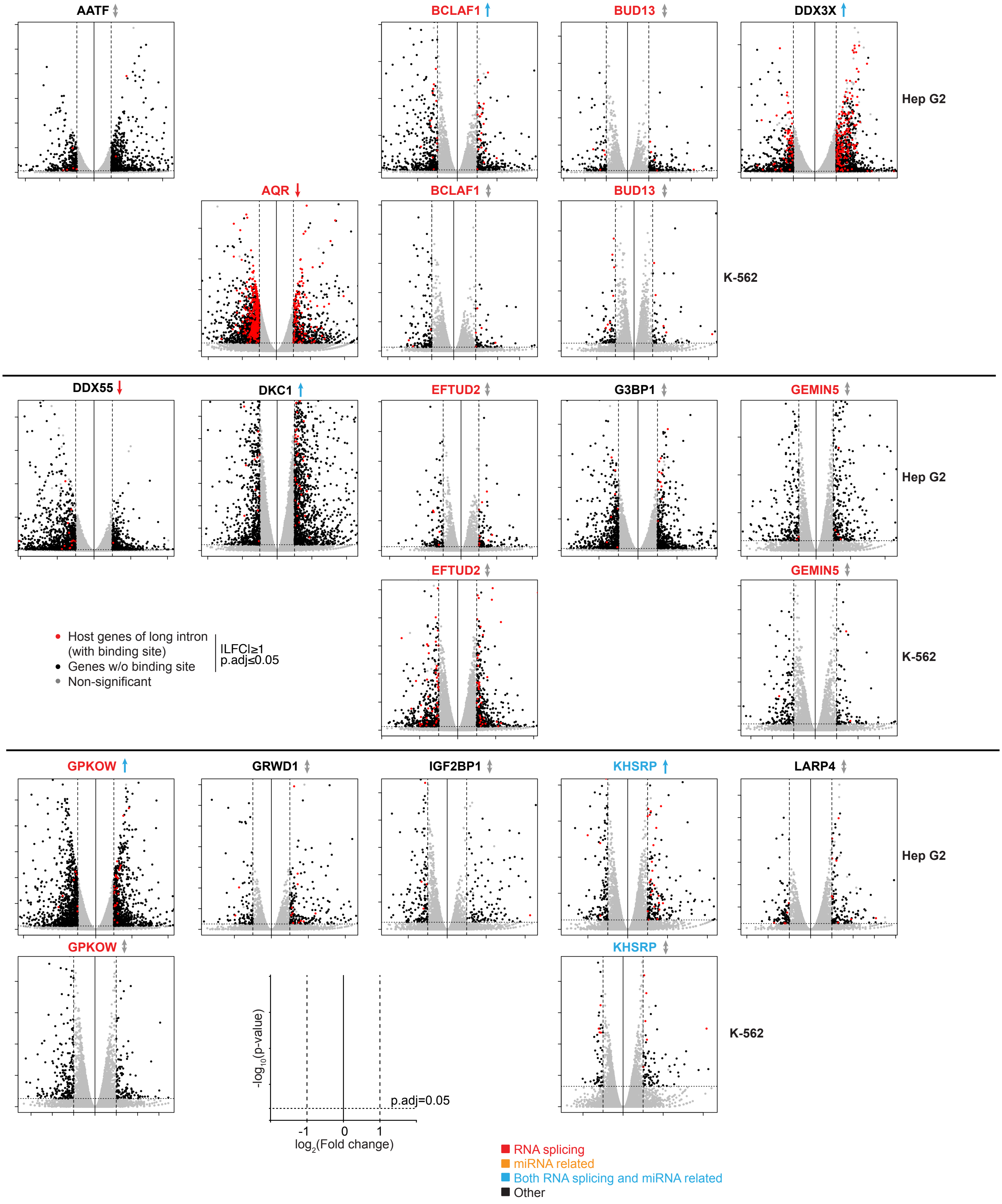

C. Long introns

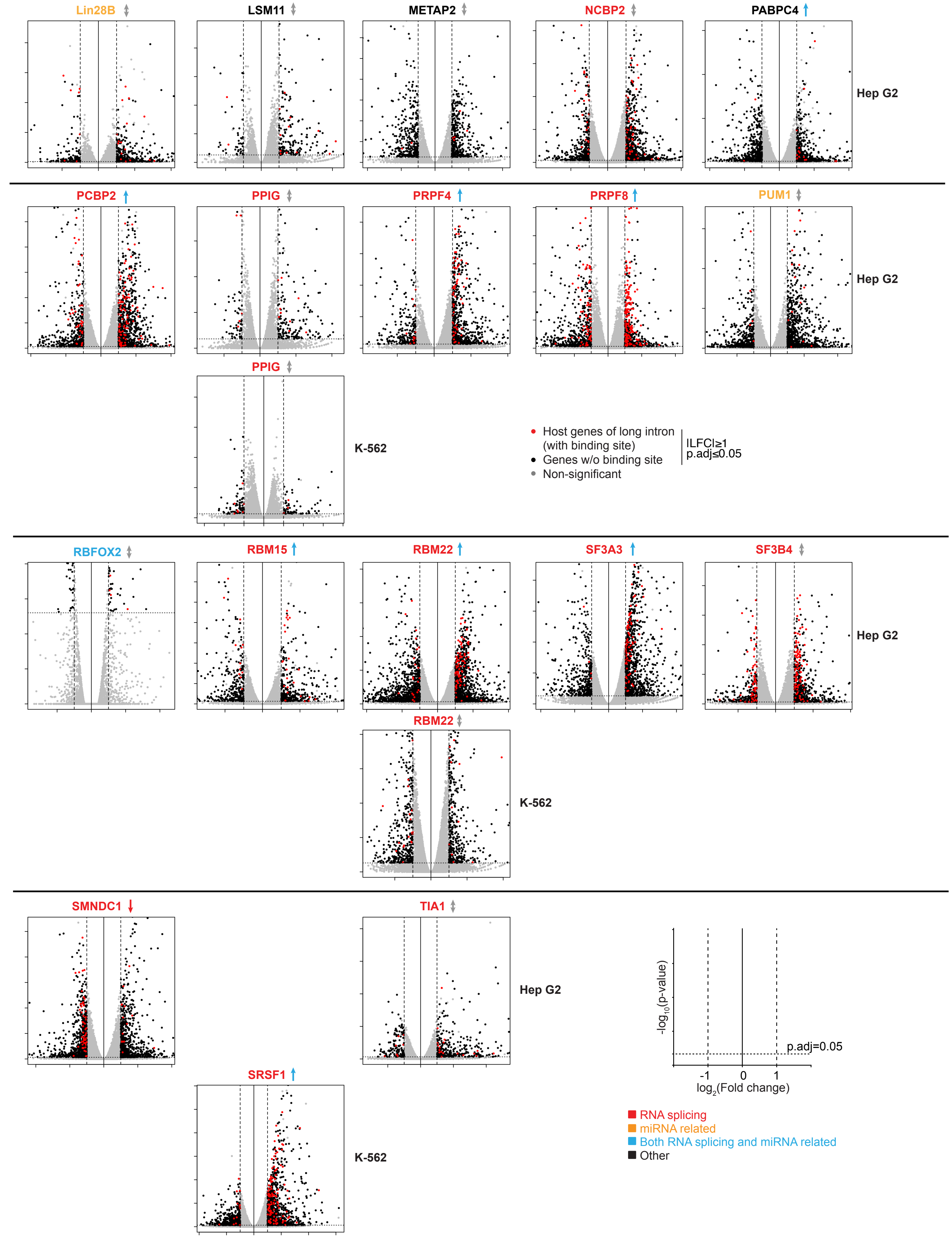

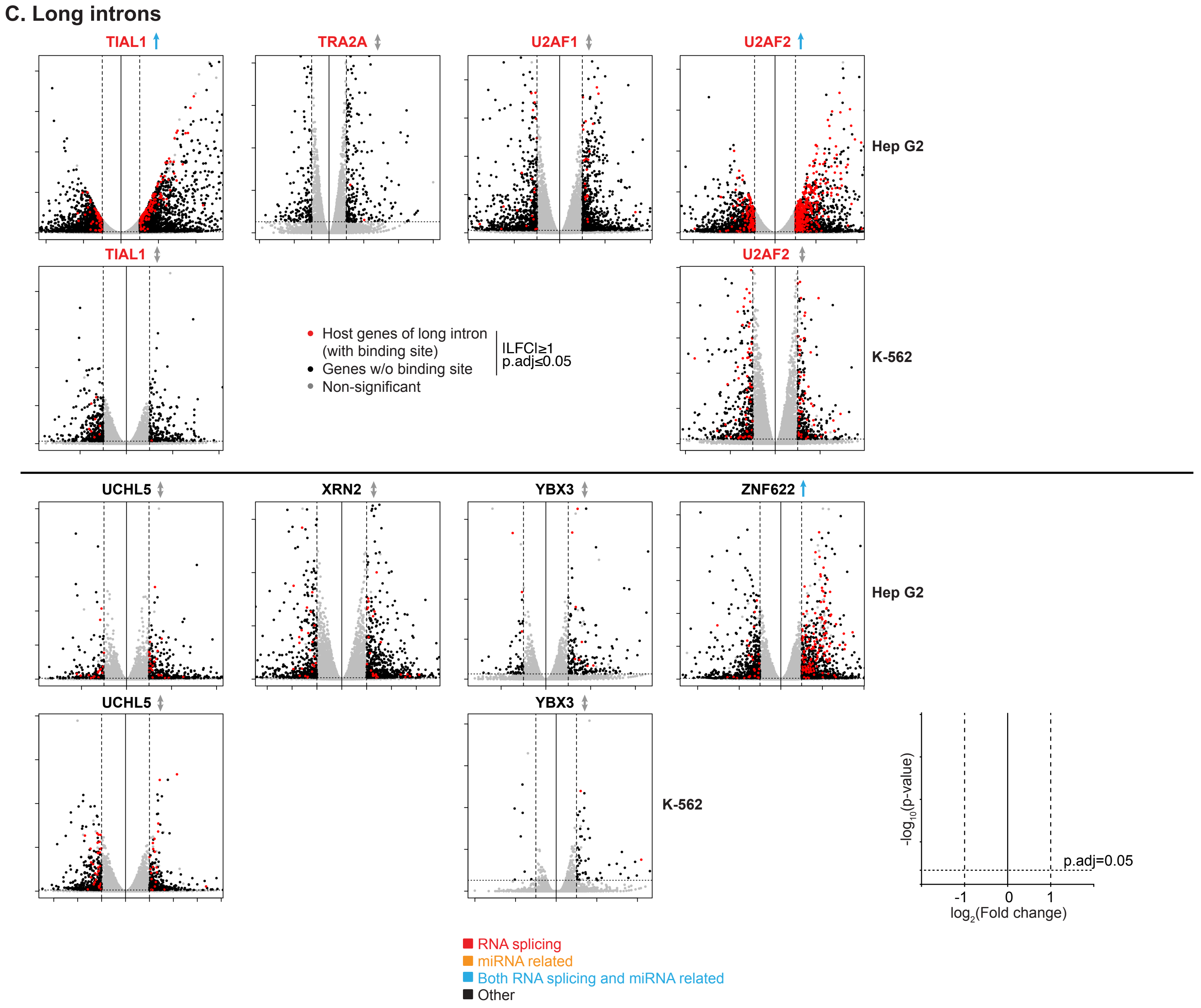

S13 Fig. Changes in mRNA levels of host genes of FLEXIs, other short, and long introns with binding sites for different RBPs in RBP-knockdown versus control datasets.

Volcano plots showing -$\log_{10}$-transformed adjusted p-values versus $\log_2$-transformed fold changes for ENSEMBL-annotated genes in ENCODE knockdown versus control datasets for the indicated RBPs in K-562 and Hep G2 cells. Host genes of **(A)** FLEXIs, **(B)** other short introns, and **(C)** long introns that contain an annotated binding site for the indicated RBP that have significant differential expression (DE) in mRNA levels (adjusted p≤0.05, |LFC|≥1) in the knockdown datasets are shown as red dots. Other genes with or without significant expression changes are shown as black or gray dots, respectively. RBPs whose knockdown resulted in a significant bias towards increased or decreased mRNA levels from host genes encoding FLEXIs, other short introns, and long introns with an annotated binding site for the RBP compared to genes whose transcript lack an annotated binding site for the same RBP are indicated by up (light blue) or down (red) arrows, respectively next to the RBP name. For these comparisons, significant bias is defined as p-value ≤0.05 determined by Fisher's exact test comparing the ratio of significantly up-regulated ($\log_2$FC>0, adjusted p≤0.05) or down-regulated ($\log_2$FC<0, adjusted p≤0.05) host genes whose FLEXIs, other short introns, or long introns contain an annotated binding site for the RBP to those in all significantly changed genes whose transcripts lack an annotated binding site for the same RBP. RBP knockdowns that resulted in significant changes in mRNA levels from host genes containing a FLEXI, other short introns, and long introns with a binding site for the knocked down RBP, but no significant directional bias, are indicated by a (gray) bi-directional arrow next to the RBP name. Plots are shown only from those RBPs whose knockdown resulted in a significant difference (p ≤0.05) in the number of DE genes whose FLEXIs, other short introns, or long introns contain an annotated binding site for the RBP. Datasets that were not available for an RBP in one of the two cell lines were left as a blank space. RBP names are color coded by protein function as shown at the bottom of the Figure.
