# Supplementary material for: Human cells contain myriad excised linear intron RNAs with links to gene regulation and potential utility as biomarkers
Source: PLoS Genet. 2024 Sep 26;20(9):e1011416. doi: 10.1371/journal.pgen.1011416 (PMC11460701; doi:10.1371/journal.pgen.1011416)
Supplement: S14 Fig — Splicing changes for (A) FLEXIs, (B) other short introns, or (C) long introns in ENCODE knockdown datasets for Hep G2 and K-562 cells were calculated using rMATS (https://rnaseq-mats.sourceforge.io). The Figure shows Empirical Cumulative Distribution Function (ECDF) plots for inclusion of retained introns (RI) or skipped exons (SE) adjacent to introns that have (red) or do not have (gray) a CLIP-seq-identified binding site for the indicated RBP in ENCODE knockdown datasets. Red curves shifted to the right or left of the control (gray) indicate an increase or decrease, respectively, in retained introns and skipped exons as indicated at the top of each set of plots. Statistical significance was calculated by Kolmogorov-Smirnov test. Plots are shown only from those RBPs whose knockdown resulted in a significant change (p≤0.05). Names of RBPs are color coded by protein function as indicated in the Figure. Blank spaces were left for datasets that were not available for an RBP in one of the two cell lines. Axes labels are shown in the key at the bottom right. (PDF) [file pgen.1011416.s014.pdf]

A. FLEXIs

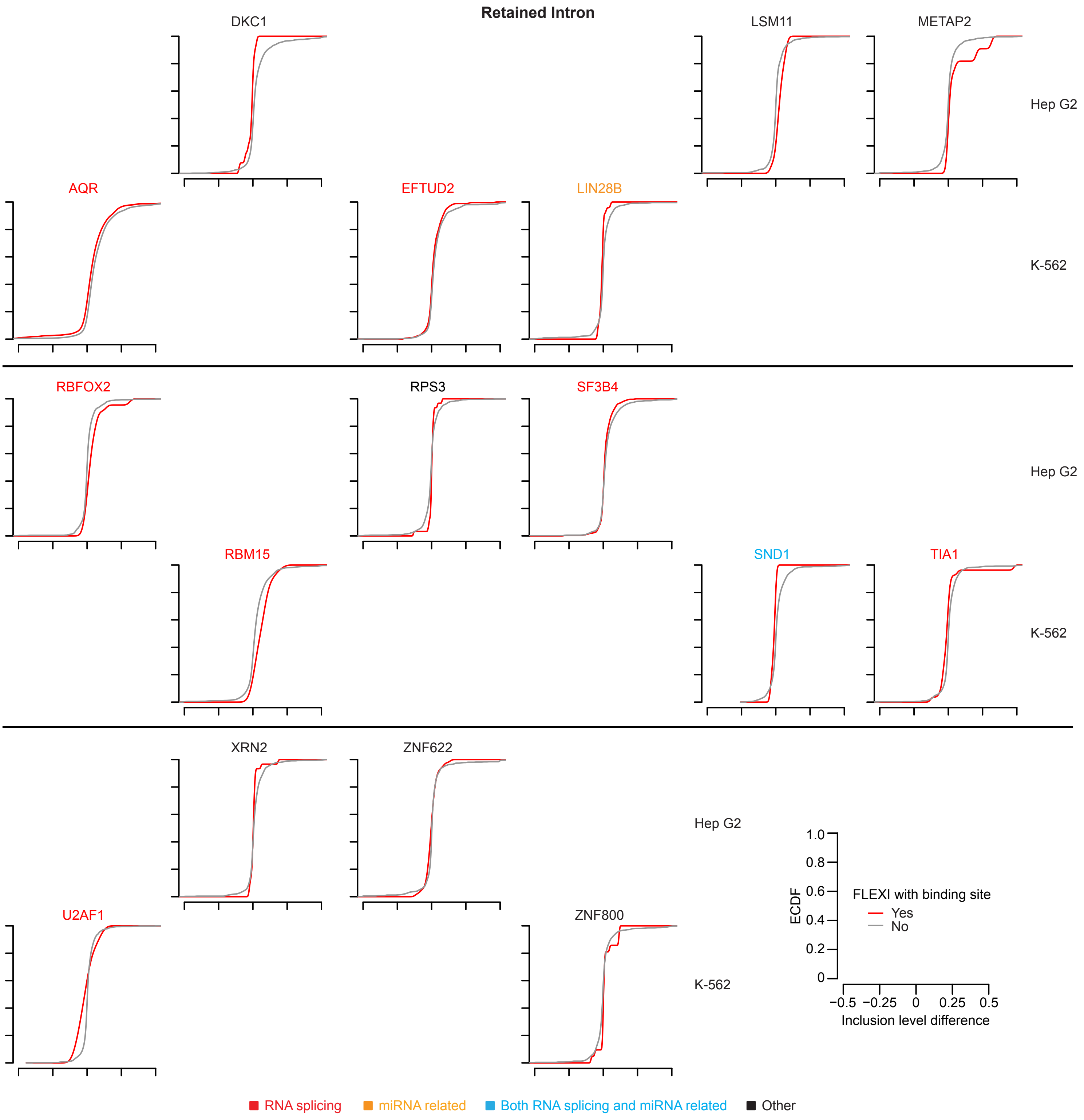
Retained Intron
DKC1
LSM11
METAP2
Hep G2
AQR
EFTUD2
LIN28B
K-562
RBFOX2
RPS3
SF3B4
Hep G2
RBM15
SND1
TIA1
K-562
XRN2
ZNF622
Hep G2
U2AF1
ZNF800
K-562
1.0
0.8
0.6
0.4
0.2
0
ECDF
FLEXI with binding site
Yes
No
−0.5 −0.25 0 0.25 0.5
Inclusion level difference
RNA splicing
miRNA related
Both RNA splicing and miRNA related
Other

A. FLEXIs

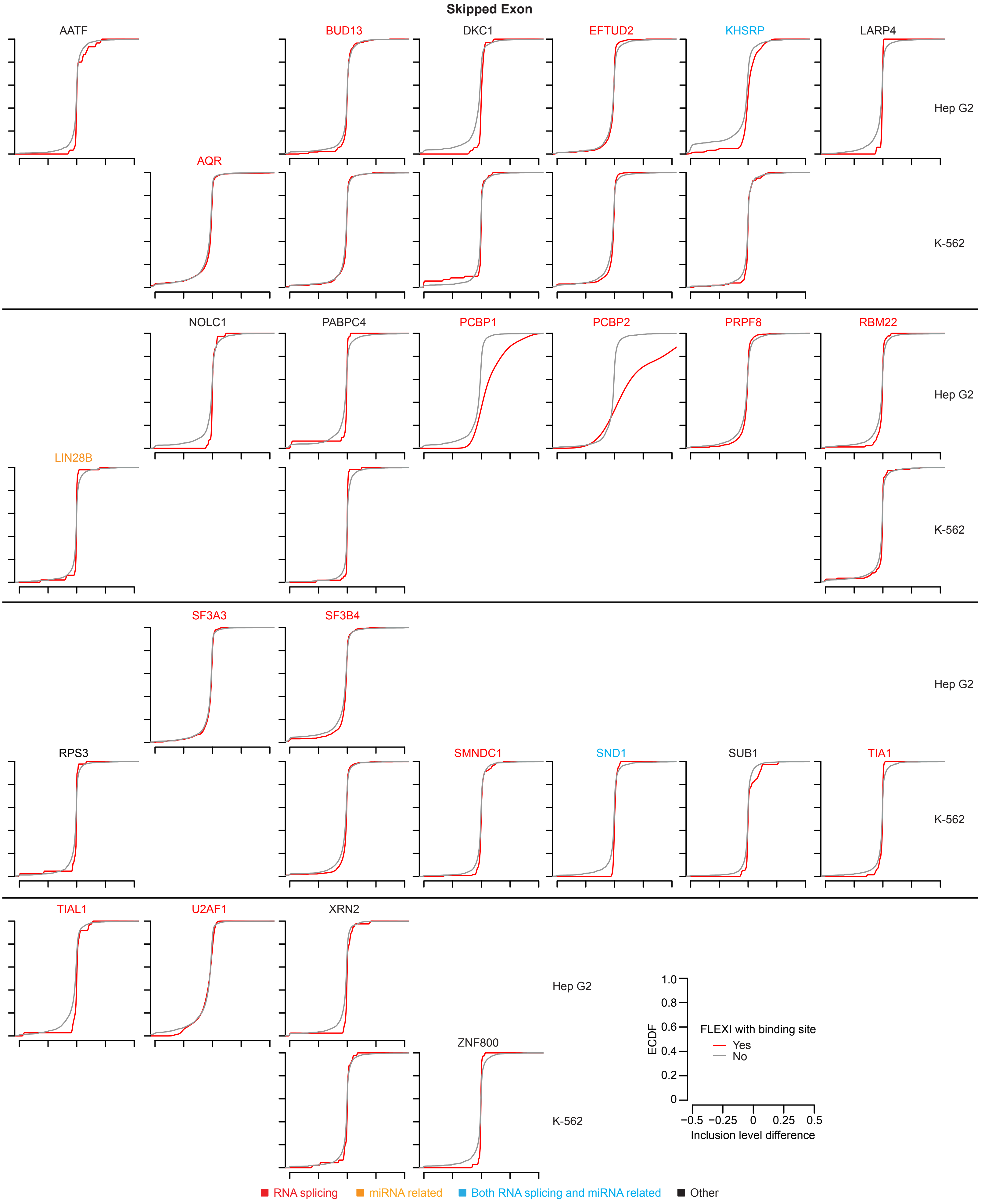
Skipped Exon
AATF
BUD13
DKC1
EFTUD2
KHSRP
LARP4
Hep G2
AQR
K-562
NOLC1
PABPC4
PCBP1
PCBP2
PRPF8
RBM22
Hep G2
LIN28B
K-562
SF3A3
SF3B4
Hep G2
RPS3
SMNDC1
SND1
SUB1
TIA1
K-562
TIAL1
U2AF1
XRN2
Hep G2
ZNF800
K-562
ECDF
1.0
0.8
0.6
0.4
0.2
0
−0.5 −0.25 0 0.25 0.5
Inclusion level difference
FLEXI with binding site
Yes
No
RNA splicing
miRNA related
Both RNA splicing and miRNA related
Other

B. Other short introns

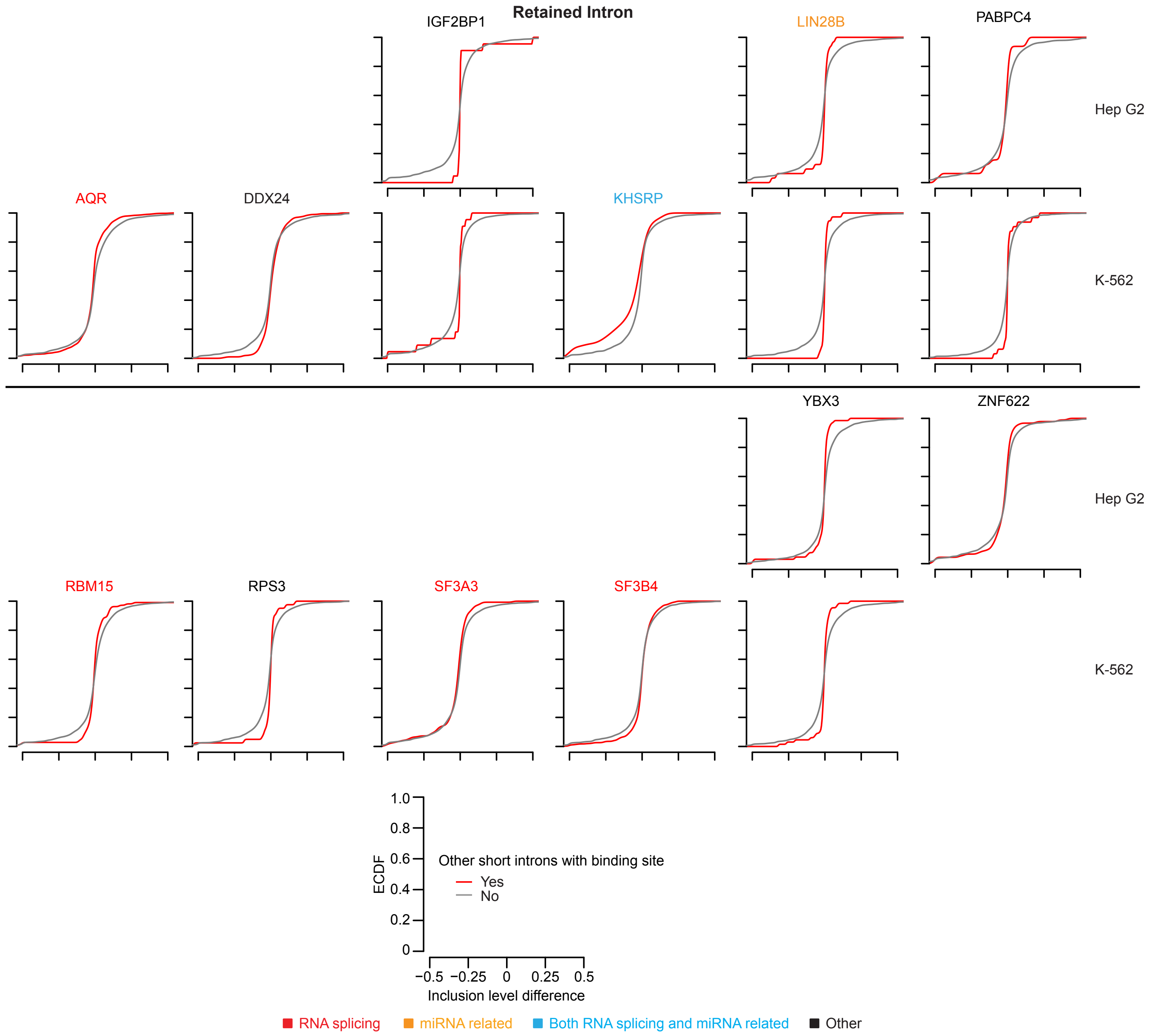

B. Other short introns

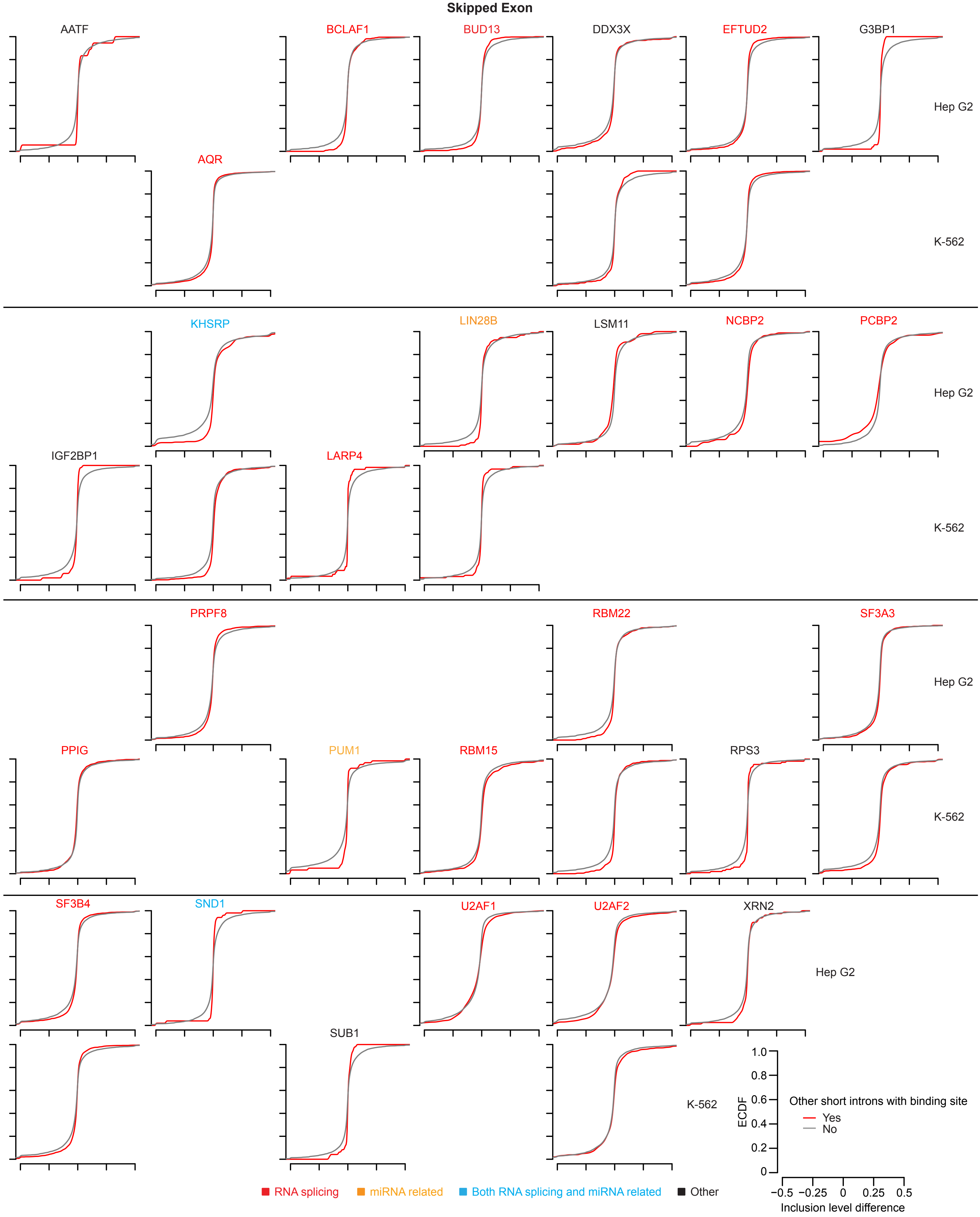

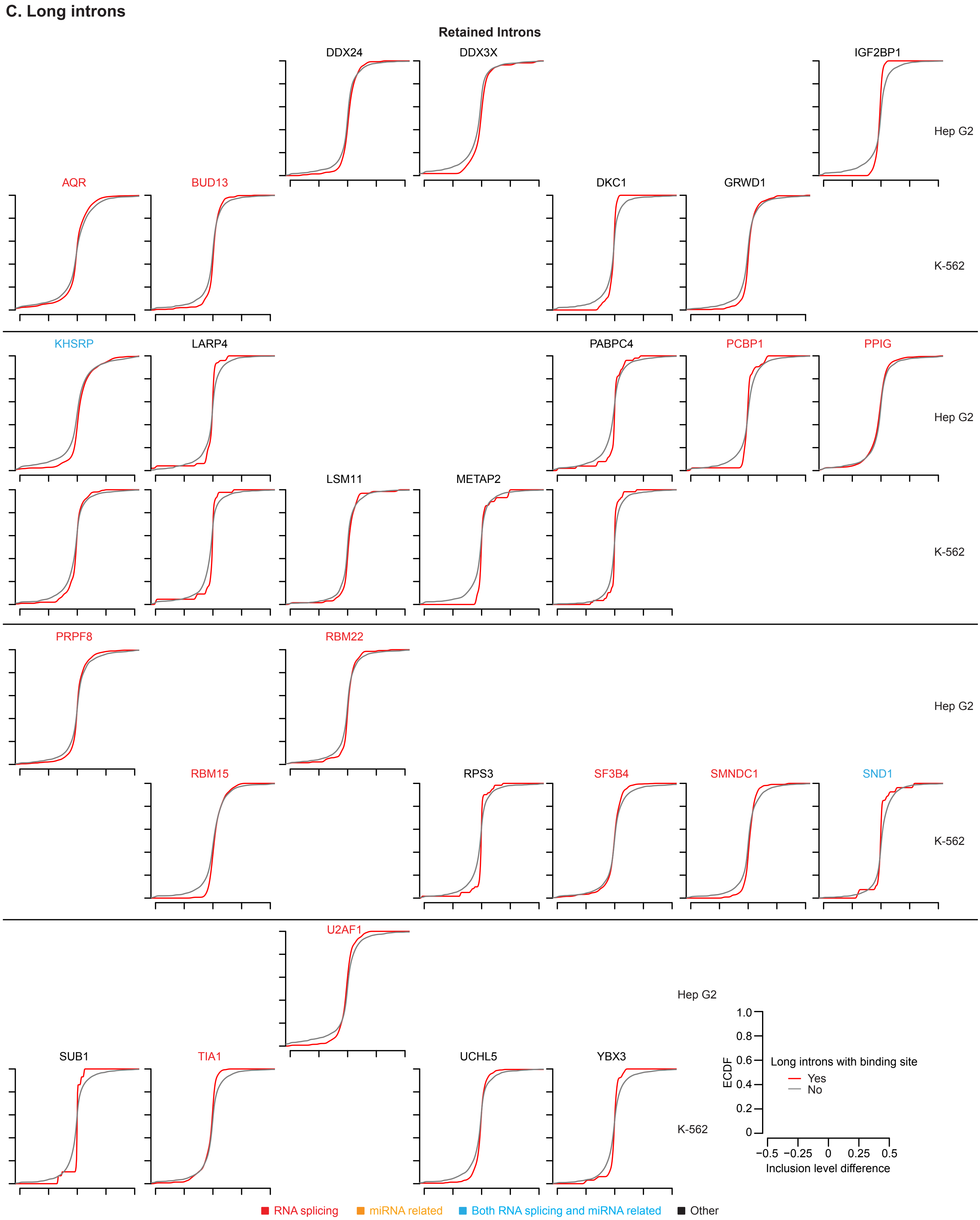
C. Long introns
Retained Introns
DDX24
DDX3X
IGF2BP1
Hep G2
AQR
BUD13
DKC1
GRWD1
K-562
KHSRP
LARP4
PABPC4
PCBP1
PPIG
Hep G2
LSM11
METAP2
K-562
PRPF8
RBM22
Hep G2
RBM15
RPS3
SF3B4
SMNDC1
SND1
K-562
U2AF1
Hep G2
SUB1
TIA1
UCHL5
YBX3
K-562
ECDF
1.0
0.8
0.6
0.4
0.2
0
−0.5
−0.25
0
0.25
0.5
Inclusion level difference
Long introns with binding site
Yes
No
RNA splicing
miRNA related
Both RNA splicing and miRNA related
Other

C. Long introns

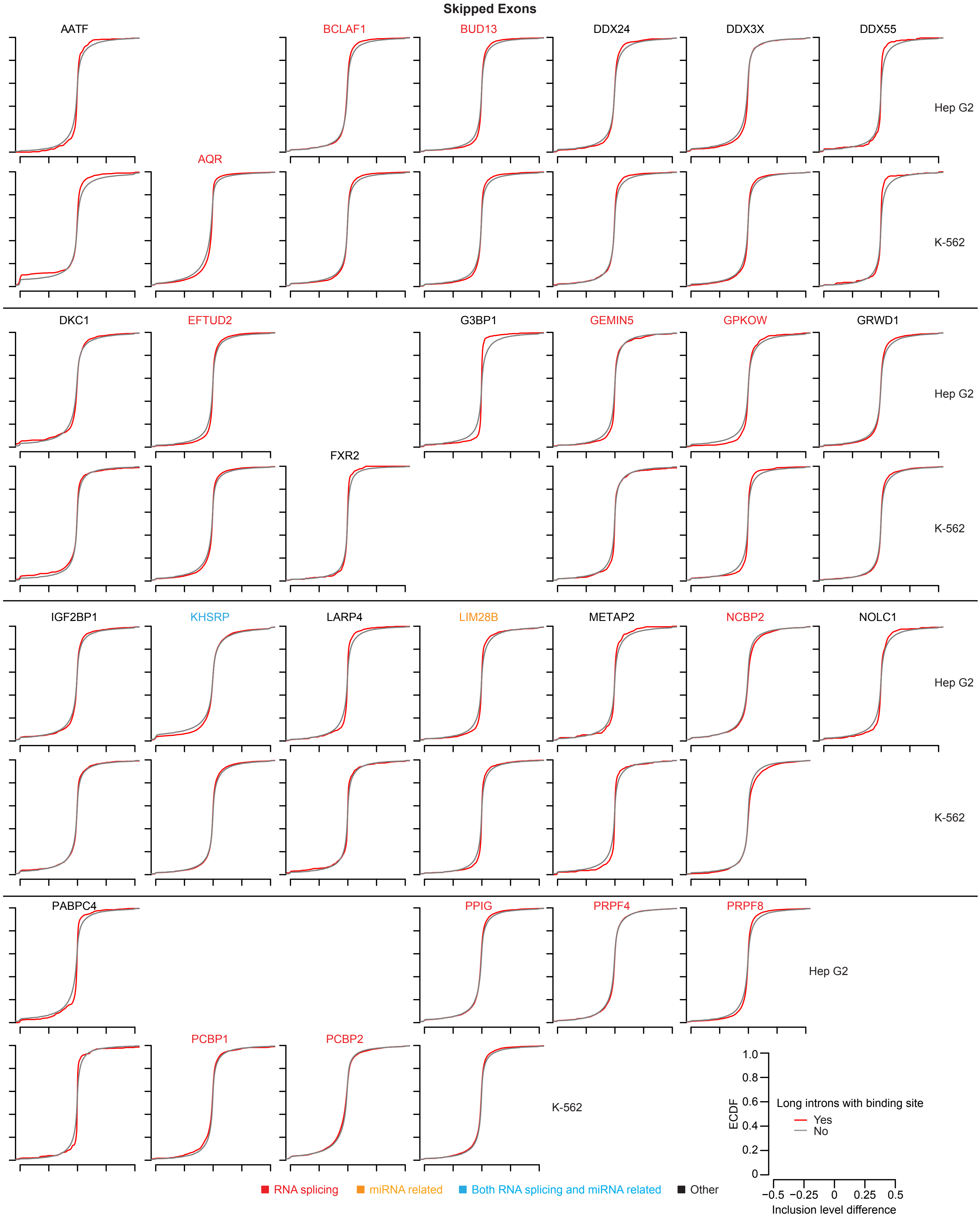

C. Long introns

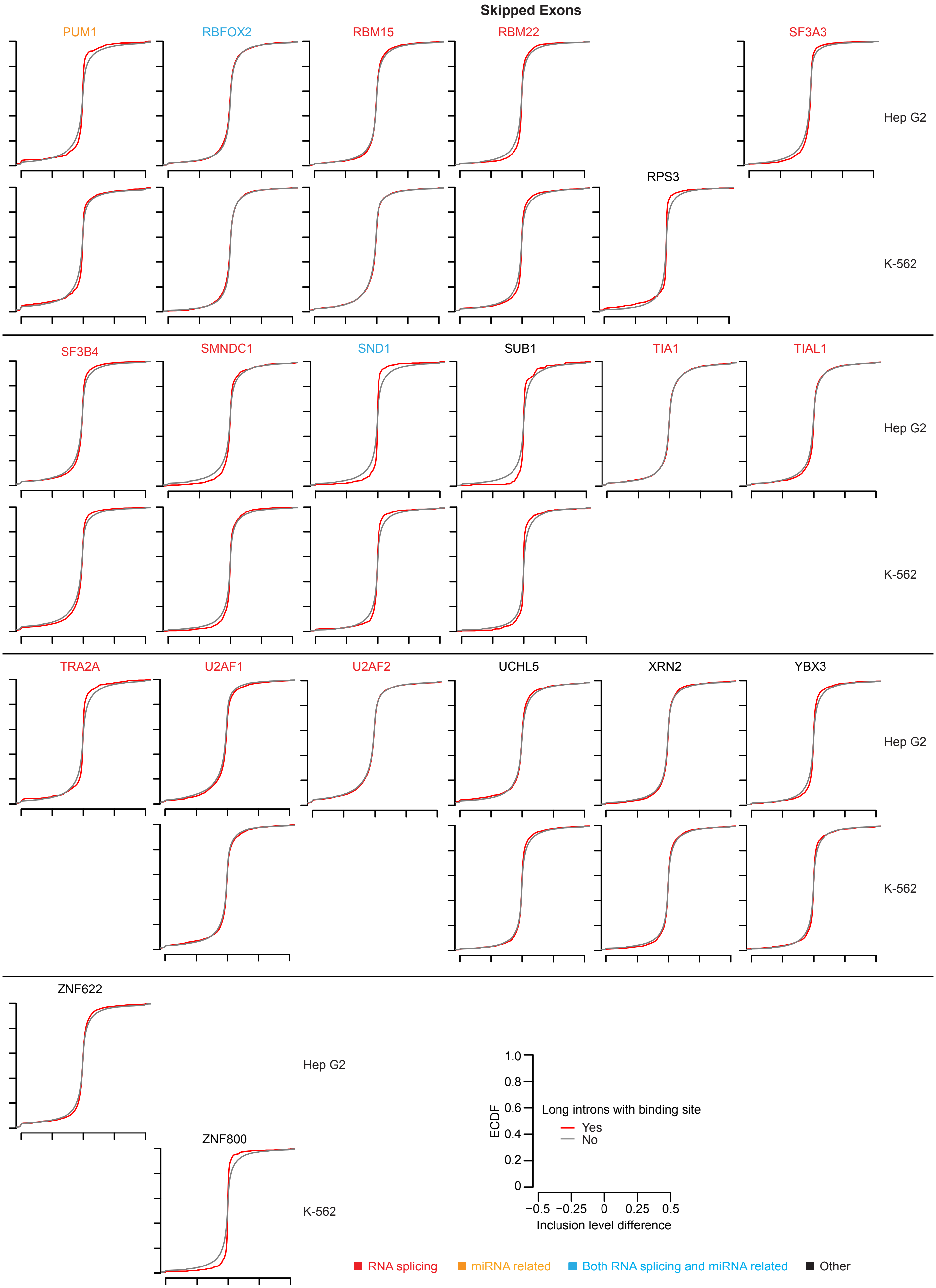

S14 Fig. Splicing changes at or adjacent to different classes of introns in RBP-knockdown versus control datasets.

Splicing changes for **(A)** FLEXIs, **(B)** other short introns, or **(C)** long introns in ENCODE knockdown datasets for Hep G2 and K-562 cells were calculated using rMATS (https://rnaseq-mats.sourceforge.io). The Figure shows Empirical Cumulative Distribution Function (ECDF) plots for inclusion of retained introns (RI) or skipped exons (SE) adjacent to introns that have (red) or do not have (gray) a CLIP-seq-identified binding site for the indicated RBP in ENCODE knockdown datasets. Red curves shifted to the right or left of the control (gray) indicate an increase or decrease, respectively, in retained introns and skipped exons as indicated at the top of each set of plots. Statistical significance was calculated by Kolmogorov-Smirnov test. Plots are shown only from those RBPs whose knockdown resulted in a significant change ($p \leq 0.05$). Names of RBPs are color coded by protein function as indicated in the Figure. Blank spaces were left for datasets that were not available for an RBP in one of the two cell lines. Axes labels are shown in the key at the bottom right.
