# Supplementary material for: Human cells contain myriad excised linear intron RNAs with links to gene regulation and potential utility as biomarkers
Source: PLoS Genet. 2024 Sep 26;20(9):e1011416. doi: 10.1371/journal.pgen.1011416 (PMC11460701; doi:10.1371/journal.pgen.1011416)
Supplement: S15 Fig — FLEXIs containing at least one annotated binding site for any RBP in any of the 4 cell lines (1,261 to 2,404 FLEXIs in different cell lines) were used in this analysis. For hierarchical clustering, subsets of FLEXIs (6 to 1,086) that have binding sites for each of the 47 non-core spliceosomal RBPs with binding sites for ≥30 different FLEXI in a merged TGIRT-seq dataset for the 4 cell lines were extracted for each of the cellular RNA sample types. Contingency tables were then generated by comparing the frequency of binding sites for all 126 RBPs that have an identified binding site in a FLEXI RNA in each of these subsets to those in all FLEXI RNAs. Multiple binding sites for the same RBP in the same FLEXI were counted as one binding site. p-values for over- and under-represented RBP-binding sites calculated by Fisher’s exact test were adjusted by the Benjamini-Hochberg procedure. RBP-binding sites (≥2% abundance) were then key-coded as not significantly different or as significantly over- or under-represented in the tested subset of FLEXIs compared to all FLEXIs (adjusted p≤0.05 calculated by Fisher’s exact test and adjusted by the Benjamini-Hochberg procedure), and the key-coded information was used to construct matrices of the Gower’s distance between the 47 subsets in each of the cellular RNA samples that were used as input for hierarchical clustering by the complete linkage clustering method [81]. The results were displayed as a two-dimensional heat map to identify subsets of FLEXIs showing similar patterns of significantly over- and under-represented RBP-binding sites in each of the cellular RNA samples, with the color scale at the bottom based on -log10-transformed adjusted p-values. Significantly co-enriched RBPs are indicated by an X in the heatmap box, and clusters of co-enriched RBPs are delineated in larger boxes. RBP names are color coded by protein function as indicated at the bottom of the Figure. (PDF) [file pgen.1011416.s015.pdf]

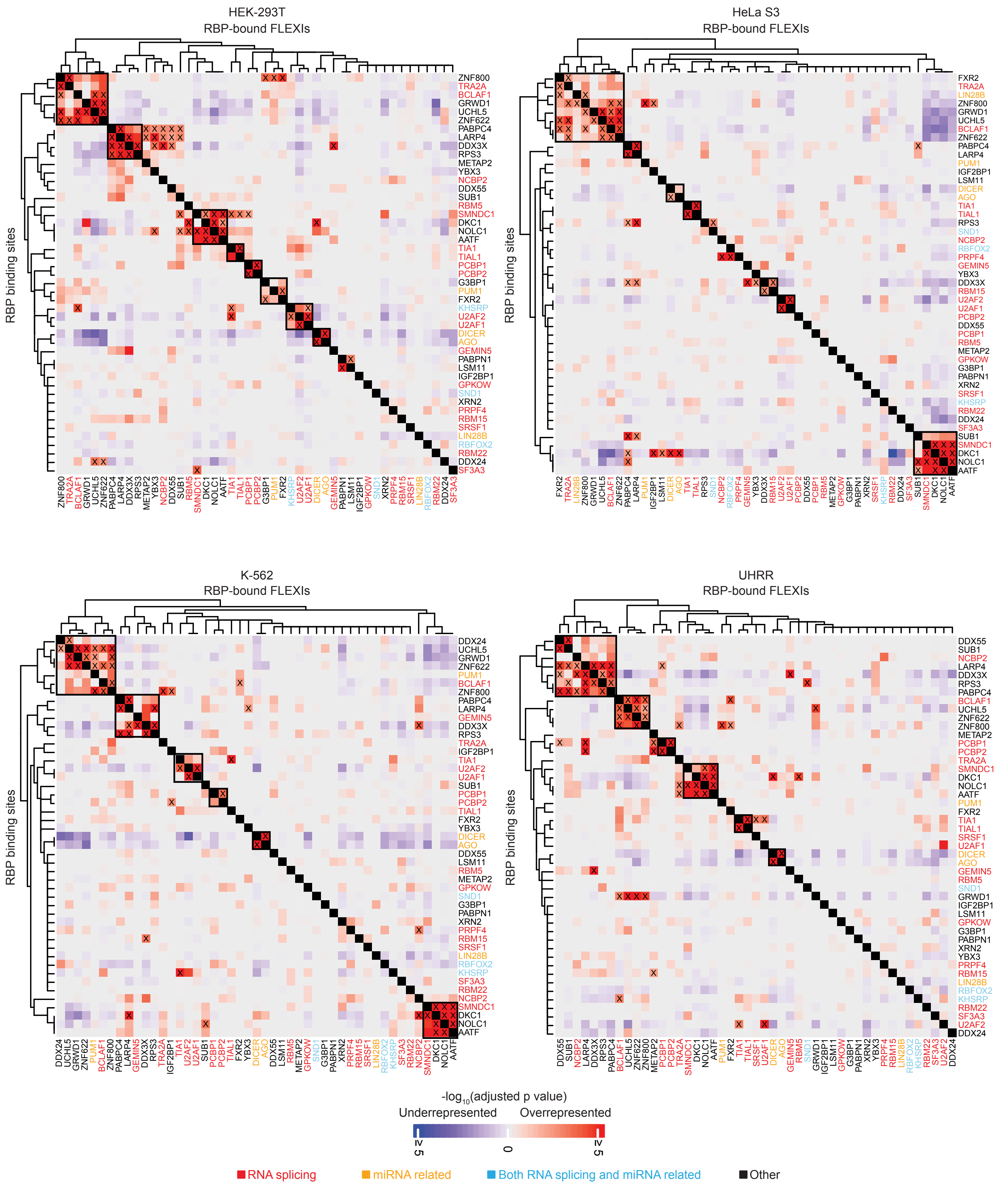
HEK-293T
RBP-bound FLEXIs
RBP binding sites
ZNF800
TRA2A
BCLAF1
GRWD1
UCHL5
ZNF622
PABPC4
LARP4
DDX3X
RPS3
METAP2
YBX3
NCBP2
DDX55
SUB1
RBM5
SMNDC1
DKC1
NOLC1
AATF
TIA1
TIAL1
PCBP1
PCBP2
G3BP1
PUM1
FXR2
KHSRP
U2AF2
U2AF1
DICER
AGO
GEMIN5
PABPN1
LSM11
IGF2BP1
GPKOW
SND1
XRN2
PRPF4
RBM15
SRSF1
LIN28B
RBFOX2
RBM22
DDX24
SF3A3
HeLa S3
RBP-bound FLEXIs
RBP binding sites
FXR2
TRA2A
LIN28B
ZNF800
GRWD1
UCHL5
BCLAF1
ZNF622
PABPC4
LARP4
PUM1
IGF2BP1
LSM11
DICER
AGO
TIA1
TIAL1
RPS3
SND1
NCBP2
RBFOX2
PRPF4
GEMIN5
YBX3
DDX3X
RBM15
U2AF2
U2AF1
PCBP2
DDX55
PCBP1
RBM5
METAP2
GPKOW
G3BP1
PABPN1
XRN2
SRSF1
KHSRP
RBM22
DDX24
SF3A3
SUB1
SMNDC1
DKC1
NOLC1
AATF
K-562
RBP-bound FLEXIs
RBP binding sites
DDX24
UCHL5
GRWD1
ZNF622
PUM1
BCLAF1
ZNF800
PABPC4
LARP4
GEMIN5
DDX3X
RPS3
TRA2A
IGF2BP1
TIA1
U2AF2
U2AF1
SUB1
PCBP1
PCBP2
TIAL1
FXR2
YBX3
DICER
AGO
DDX55
LSM11
RBM5
METAP2
GPKOW
SND1
G3BP1
PABPN1
XRN2
PRPF4
RBM15
SRSF1
LIN28B
RBFOX2
KHSRP
SF3A3
RBM22
NCBP2
SMNDC1
DKC1
NOLC1
AATF
UHRR
RBP-bound FLEXIs
RBP binding sites
DDX55
SUB1
NCBP2
LARP4
DDX3X
RPS3
PABPC4
BCLAF1
UCHL5
ZNF622
ZNF800
METAP2
PCBP1
PCBP2
TRA2A
SMNDC1
DKC1
NOLC1
AATF
PUM1
FXR2
TIA1
TIAL1
SRSF1
U2AF1
DICER
AGO
GEMIN5
RBM5
SND1
GRWD1
IGF2BP1
LSM11
GPKOW
G3BP1
PABPN1
XRN2
YBX3
PRPF4
RBM15
LIN28B
RBFOX2
KHSRP
RBM22
SF3A3
U2AF2
DDX24
-log10(adjusted p value)
Underrepresented
Overrepresented
≥5
0
≥5
RNA splicing
miRNA related
Both RNA splicing and miRNA related
Other

S15 Fig. Hierarchical clustering of FLEXIs in different cell lines based on over- and under-represented RBP-binding sites.

FLEXIs containing at least one annotated binding site for any RBP in any of the 4 cell lines (1,261 to 2,404 FLEXIs in different cell lines) were used in this analysis. For hierarchical clustering, subsets of FLEXIs (6 to 1,086) that have binding sites for each of the 47 non-core spliceosomal RBPs with binding sites for ≥30 different FLEXI in a merged TGIRT-seq dataset for the 4 cell lines were extracted for each of the cellular RNA sample types. Contingency tables were then generated by comparing the frequency of binding sites for all 126 RBPs that have an identified binding site in a FLEXI RNA in each of these subsets to those in all FLEXI RNAs. Multiple binding sites for the same RBP in the same FLEXI were counted as one binding site. p-values for over- and under-represented RBP-binding sites calculated by Fisher's exact test were adjusted by the Benjamini-Hochberg procedure. RBP-binding sites (≥2% abundance) were then key-coded as not significantly different or as significantly over- or under-represented in the tested subset of FLEXIs compared to all FLEXIs (adjusted $p \leq 0.05$ calculated by Fisher's exact test and adjusted by the Benjamini-Hochberg procedure), and the key-coded information was used to construct matrices of the Gower's distance between the 47 subsets in each of the cellular RNA samples that were used as input for hierarchical clustering by the complete linkage clustering method (79). The results were displayed as a two-dimensional heat map to identify subsets of FLEXIs showing similar patterns of significantly over- and under-represented RBP-binding sites in each of the cellular RNA samples, with the color scale at the bottom based on $-\log_{10}$-transformed adjusted p-values. Significantly co-enriched RBPs are indicated by an X in the heatmap box, and clusters of co-enriched RBPs are delineated in larger boxes. RBP names are color coded by protein function as indicated at the bottom of the Figure.
