# Supplementary material for: Human cells contain myriad excised linear intron RNAs with links to gene regulation and potential utility as biomarkers
Source: PLoS Genet. 2024 Sep 26;20(9):e1011416. doi: 10.1371/journal.pgen.1011416 (PMC11460701; doi:10.1371/journal.pgen.1011416)
Supplement: S16 Fig — Scatter plots and density plots are shown for FLEXIs with binding sites for each protein in (A) Cluster I, (B) Cluster II, (C) Cluster III, (D) Cluster IV, (E) Cluster V, and (F) Cluster VI. In the scatter plots (left), RBPs whose binding sites were significantly over- or under-represented compared to those for other RBPs in the subset of FLEXIs compared to all other FLEXIs (≥2% abundance, p≤0.05 calculated by Fisher’s exact test and adjusted by the Benjamini-Hochberg procedure) are labeled by name color coded by protein function as shown at the bottom of the Figure. The density distribution plots (right) compare the length, GC content, and MFE for the most stable secondary structure predicted by RNAfold for subsets of FLEXIs with binding sites for each RBP associated with Clusters I to VI (red) compared to all other FLEXIs (black). The number of FLEXIs comprising each subset is indicated in parentheses next to the name of the RBP. p-values are shown at the top left of those density plots in which the distribution for the subset of FLEXIs differed significantly from other FLEXIs (p<0.01 and FDR≤0.05 as determined by 1,000 Monte-Carlo simulations). (PDF) [file pgen.1011416.s016.pdf]

A. Cluster I

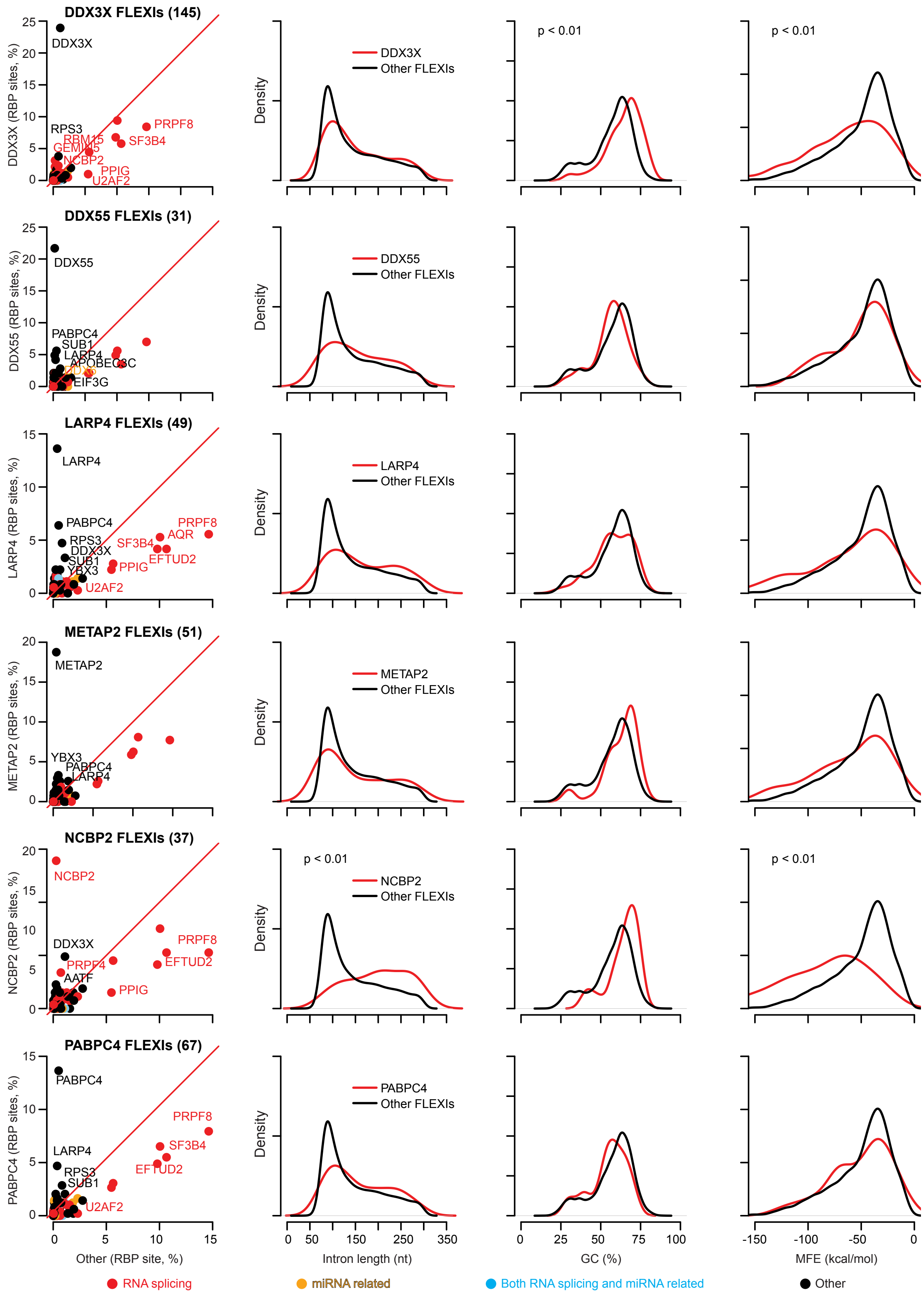

DDX3X FLEXIs (145)
DDX55 FLEXIs (31)
LARP4 FLEXIs (49)
METAP2 FLEXIs (51)
NCBP2 FLEXIs (37)
PABPC4 FLEXIs (67)
DDX3X (RBP sites, %)
DDX55 (RBP sites, %)
LARP4 (RBP sites, %)
METAP2 (RBP sites, %)
NCBP2 (RBP sites, %)
PABPC4 (RBP sites, %)
Other (RBP site, %)
DDX3X
RPS3
RBM15
GEMIN5
NCBP2
PRPF8
SF3B4
PPIG
U2AF2
DDX55
PABPC4
SUB1
LARP4
APOBEC3C
EIF3G
DDX3X
YBX3
AQR
EFTUD2
METAP2
PRPF4
AATF
Density
p < 0.01
DDX3X
Other FLEXIs
DDX55
LARP4
METAP2
NCBP2
PABPC4
Intron length (nt)
GC (%)
MFE (kcal/mol)
RNA splicing
miRNA related
Both RNA splicing and miRNA related
Other

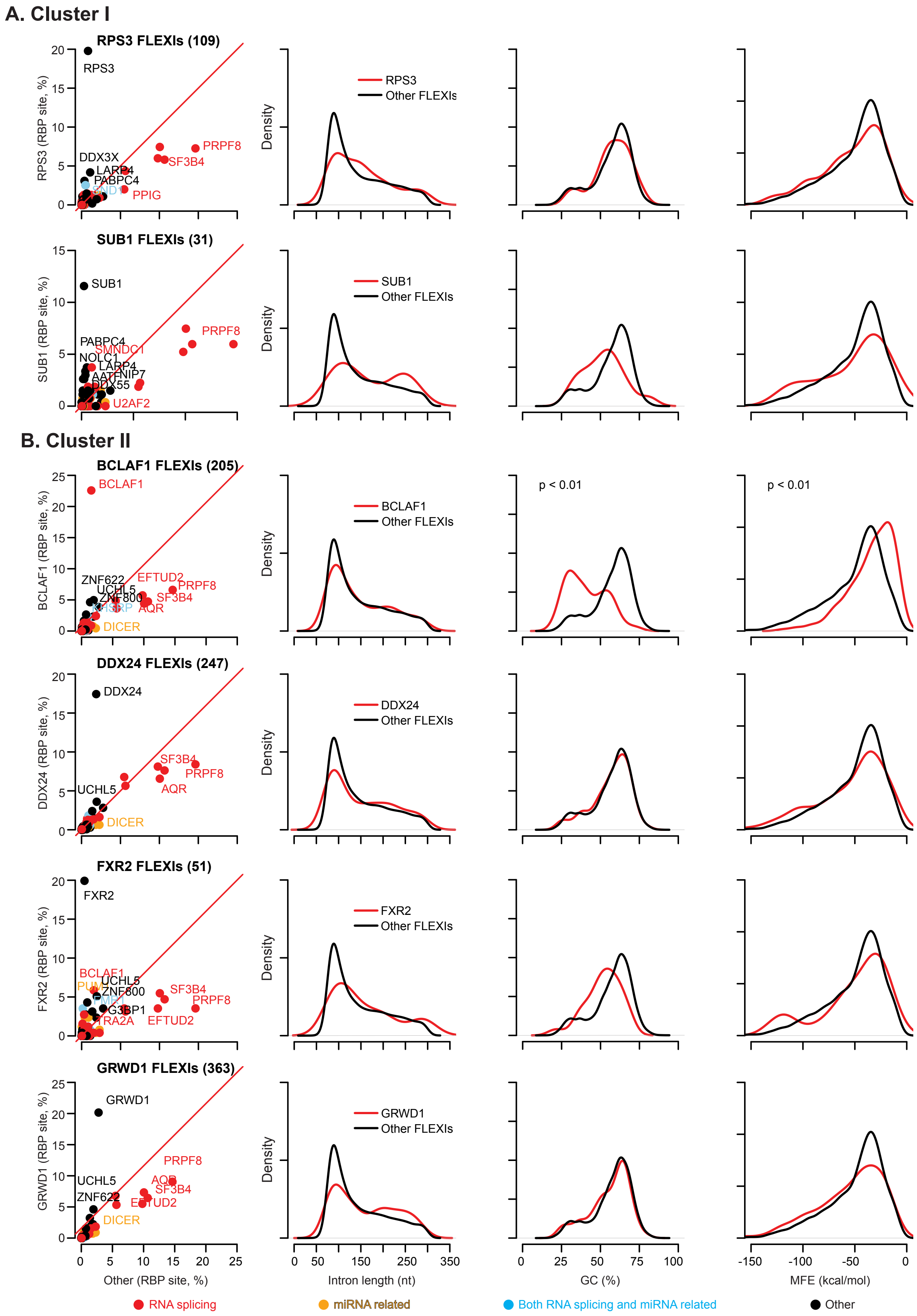

A. Cluster I
RPS3 FLEXIs (109)
RPS3 (RBP site, %)
RPS3
DDX3X
LARP4
PABPC4
PRPF8
SF3B4
PPIG
RPS3
Other FLEXIs
Density
SUB1 FLEXIs (31)
SUB1 (RBP site, %)
SUB1
PABPC4
SMNDC1
NOLC1
LARP4
AATF
NIP7
DDX55
PRPF8
U2AF2
SUB1
Other FLEXIs
B. Cluster II
BCLAF1 FLEXIs (205)
BCLAF1 (RBP site, %)
BCLAF1
ZNF622
UCHL5
ZNF800
EFTUD2
PRPF8
SF3B4
AQR
DICER
BCLAF1
Other FLEXIs
p < 0.01
p < 0.01
DDX24 FLEXIs (247)
DDX24 (RBP site, %)
DDX24
UCHL5
SF3B4
PRPF8
AQR
DICER
DDX24
Other FLEXIs
FXR2 FLEXIs (51)
FXR2 (RBP site, %)
FXR2
BCLAF1
UCHL5
ZNF800
SF3B4
PRPF8
G3BP1
TRA2A
EFTUD2
FXR2
Other FLEXIs
GRWD1 FLEXIs (363)
GRWD1 (RBP site, %)
GRWD1
PRPF8
UCHL5
AQR
SF3B4
ZNF622
EFTUD2
DICER
GRWD1
Other FLEXIs
Other (RBP site, %)
Intron length (nt)
GC (%)
MFE (kcal/mol)
RNA splicing
miRNA related
Both RNA splicing and miRNA related
Other

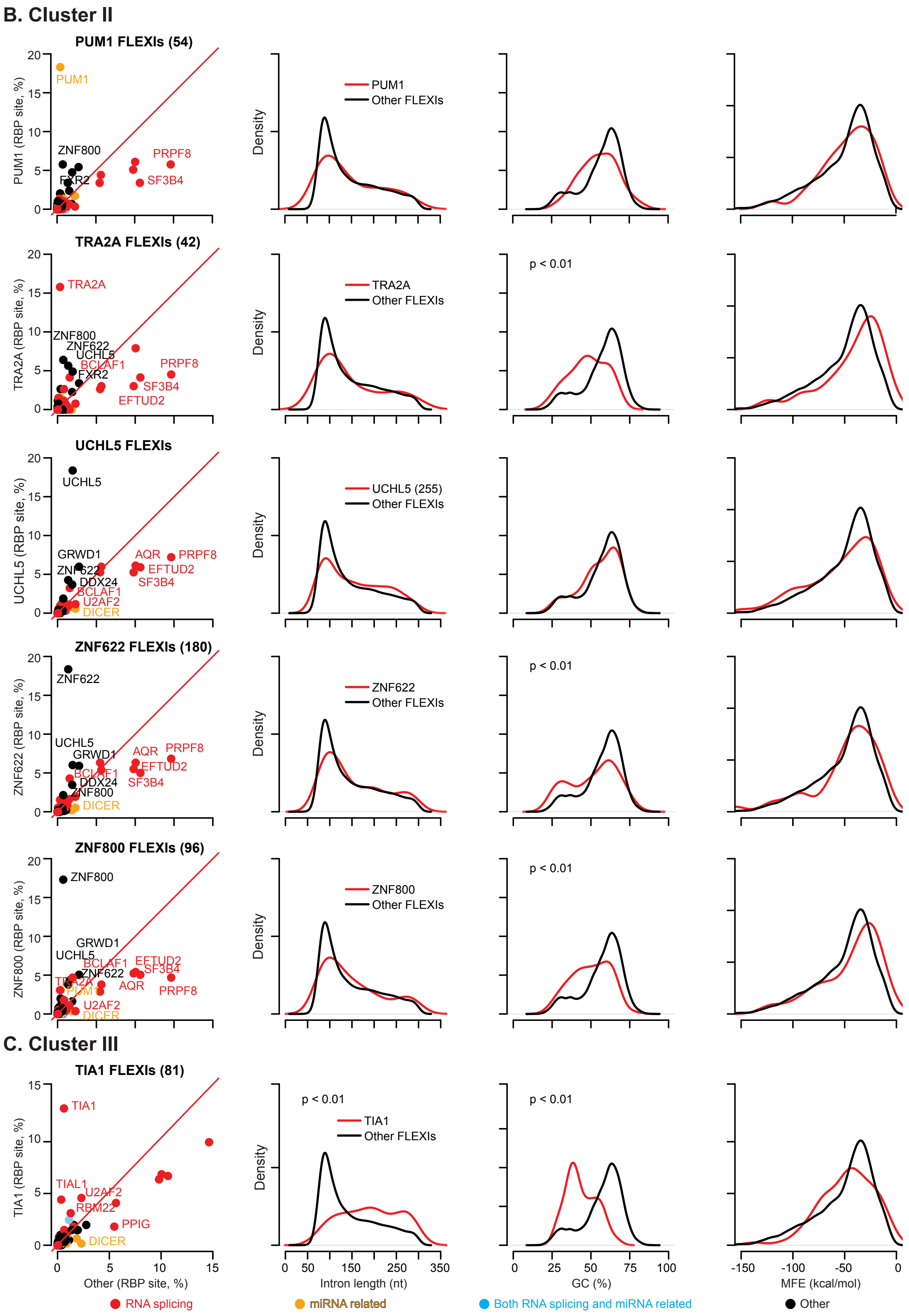

B. Cluster II
PUM1 FLEXIs (54)
PUM1 (RBP site, %)
PUM1
ZNF800
FXR2
PRPF8
SF3B4
Density
PUM1
Other FLEXIs
TRA2A FLEXIs (42)
TRA2A (RBP site, %)
TRA2A
ZNF800
ZNF622
UCHL5
BCLAF1
FXR2
PRPF8
SF3B4
EFTUD2
TRA2A
Other FLEXIs
p < 0.01
UCHL5 FLEXIs
UCHL5 (RBP site, %)
UCHL5
GRWD1
ZNF622
DDX24
BCLAF1
U2AF2
DICER
AQR
PRPF8
EFTUD2
SF3B4
UCHL5 (255)
Other FLEXIs
ZNF622 FLEXIs (180)
ZNF622 (RBP site, %)
ZNF622
UCHL5
GRWD1
BCLAF1
DDX24
ZNF800
DICER
AQR
PRPF8
EFTUD2
SF3B4
ZNF622
Other FLEXIs
p < 0.01
ZNF800 FLEXIs (96)
ZNF800 (RBP site, %)
ZNF800
GRWD1
UCHL5
BCLAF1
ZNF622
TRA2A
PUM1
U2AF2
DICER
EFTUD2
SF3B4
AQR
PRPF8
ZNF800
Other FLEXIs
p < 0.01
C. Cluster III
TIA1 FLEXIs (81)
TIA1 (RBP site, %)
TIA1
TIAL1
U2AF2
RBM22
PPIG
DICER
p < 0.01
TIA1
Other FLEXIs
p < 0.01
Other (RBP site, %)
Intron length (nt)
GC (%)
MFE (kcal/mol)
RNA splicing
miRNA related
Both RNA splicing and miRNA related
Other

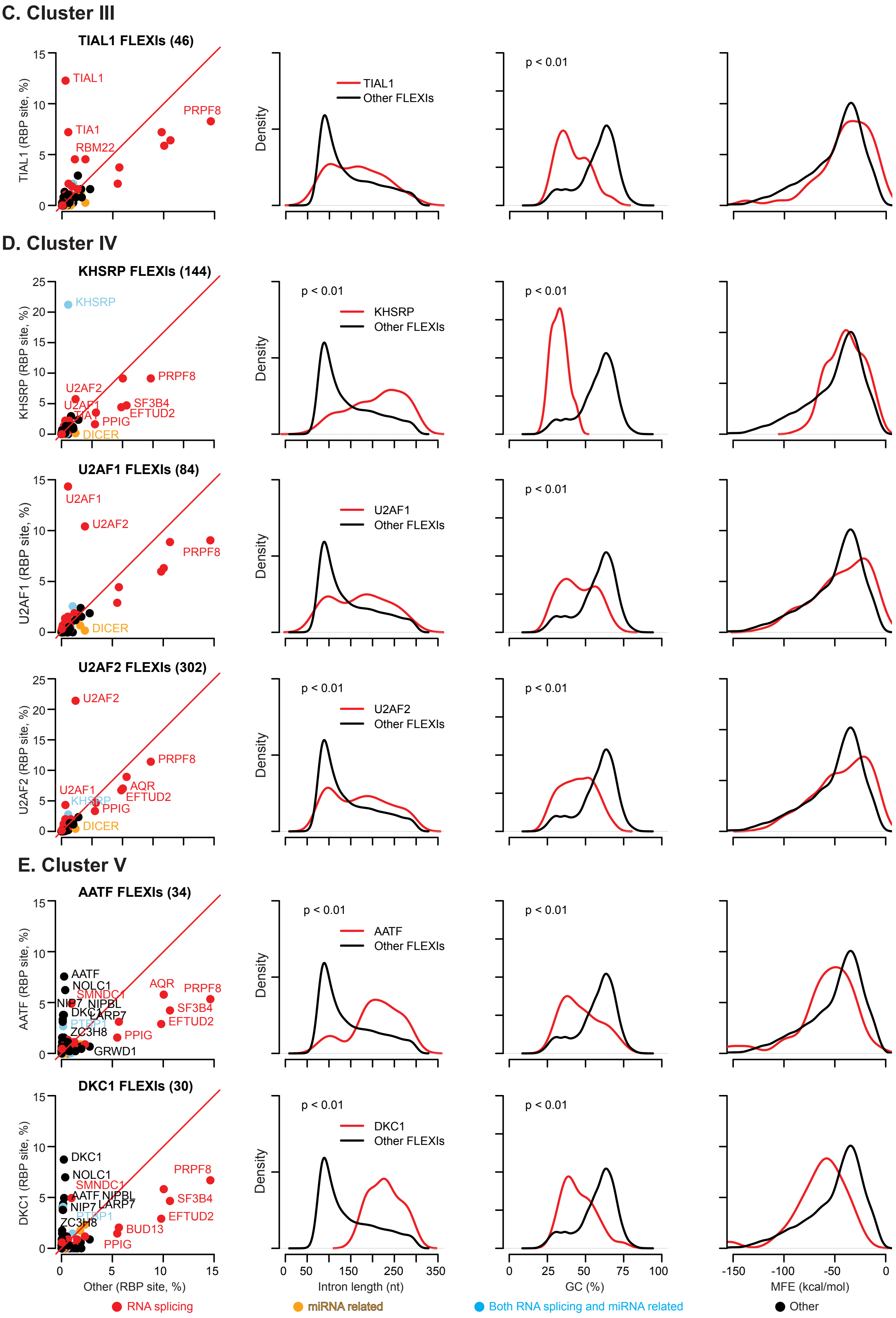
C. Cluster III
TIAL1 FLEXIs (46)
TIAL1 (RBP site, %)
TIAL1
TIA1
RBM22
PRPF8
TIAL1
Other FLEXIs
Density
p < 0.01
D. Cluster IV
KHSRP FLEXIs (144)
KHSRP (RBP site, %)
KHSRP
U2AF2
U2AF1
TIA1
PRPF8
SF3B4
EFTUD2
PPIG
DICER
p < 0.01
KHSRP
Other FLEXIs
Density
U2AF1 FLEXIs (84)
U2AF1 (RBP site, %)
U2AF1
U2AF2
PRPF8
DICER
U2AF1
Other FLEXIs
Density
p < 0.01
U2AF2 FLEXIs (302)
U2AF2 (RBP site, %)
U2AF2
PRPF8
AQR
EFTUD2
U2AF1
KHSRP
PPIG
DICER
p < 0.01
U2AF2
Other FLEXIs
Density
E. Cluster V
AATF FLEXIs (34)
AATF (RBP site, %)
AATF
NOLC1
SMNDC1
NIP7
NIPBL
DKC1
LARP7
PTBP1
ZC3H8
GRWD1
AQR
PRPF8
SF3B4
EFTUD2
PPIG
p < 0.01
AATF
Other FLEXIs
Density
DKC1 FLEXIs (30)
DKC1 (RBP site, %)
DKC1
NOLC1
SMNDC1
AATF
NIPBL
NIP7
LARP7
PTBP1
ZC3H8
BUD13
PPIG
PRPF8
SF3B4
EFTUD2
p < 0.01
DKC1
Other FLEXIs
Density
Other (RBP site, %)
Intron length (nt)
GC (%)
MFE (kcal/mol)
RNA splicing
miRNA related
Both RNA splicing and miRNA related
Other

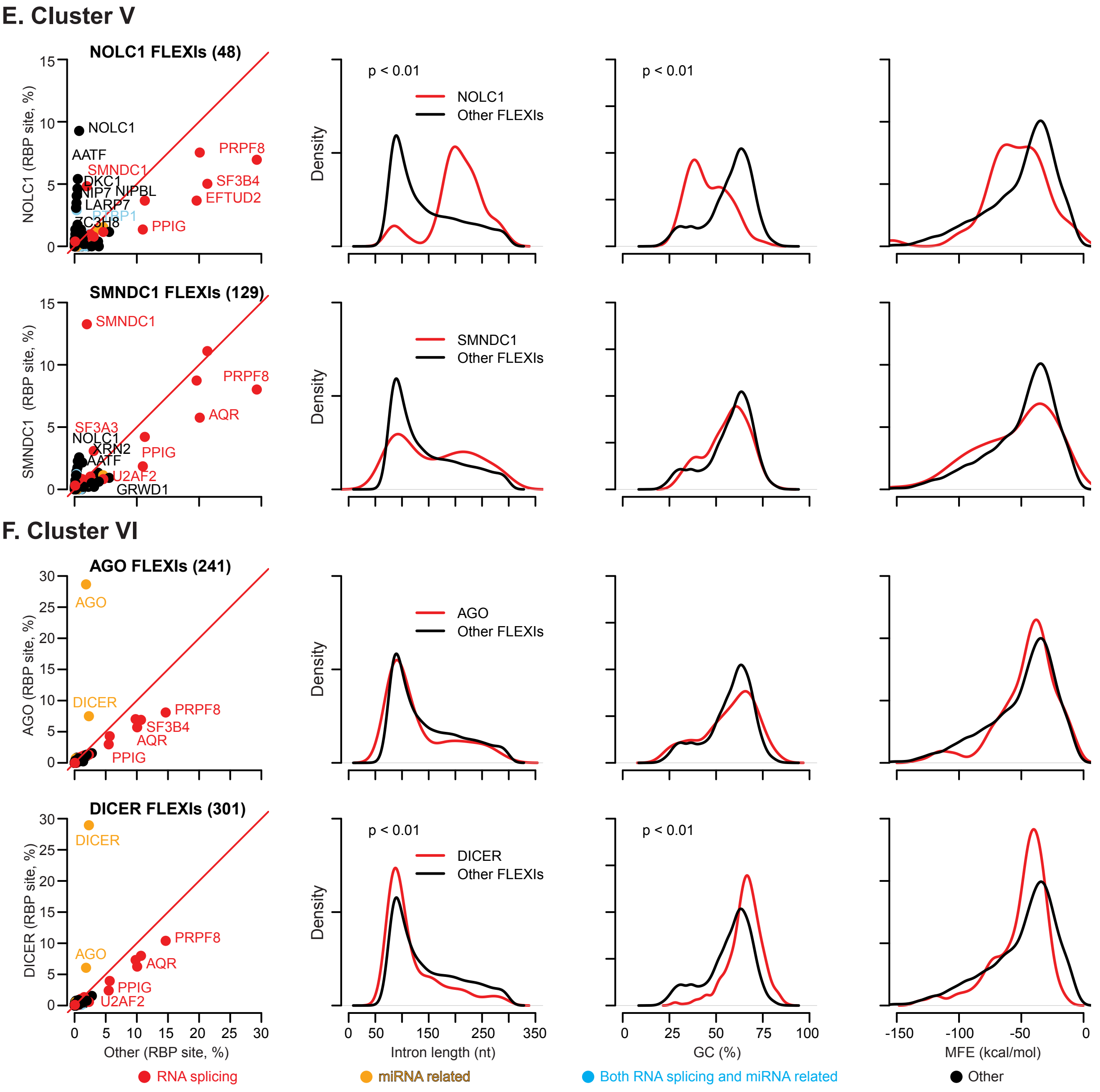

S16 Fig. Enriched RBP-binding sites and characteristics of subsets of FLEXIs with a binding site for each of the RBPs in Clusters I-VI.

Scatter plots and density plots are shown for FLEXIs with binding sites for each protein in **(A)** Cluster I, **(B)** Cluster II, **(C)** Cluster III, **(D)** Cluster IV, **(E)** Cluster V, and **(F)** Cluster VI. In the scatter plots (left), RBPs whose binding sites were significantly over- or under-represented compared to those for other RBPs in the subset of FLEXIs compared to all other FLEXIs (≥2% abundance, p≤0.05 calculated by Fisher's exact test and adjusted by the Benjamini-Hochberg procedure) are labeled by name color coded by protein function as shown at the bottom of the Figure. The density distribution plots (right) compare the length, GC content, and MFE for the most stable secondary structure predicted by RNAfold for subsets of FLEXIs with binding sites for each RBP associated with Clusters I to VI (red) compared to all other FLEXIs (black). The number of FLEXIs comprising each subset is indicated in parentheses next to the name of the RBP. p-values are shown at the top left of those density plots in which the distribution for the subset of FLEXIs differed significantly from other FLEXIs (p<0.01 and FDR ≤0.05 as determined by 1,000 Monte-Carlo simulations).
