# Supplementary material for: Human cells contain myriad excised linear intron RNAs with links to gene regulation and potential utility as biomarkers
Source: PLoS Genet. 2024 Sep 26;20(9):e1011416. doi: 10.1371/journal.pgen.1011416 (PMC11460701; doi:10.1371/journal.pgen.1011416)
Supplement: S17 Fig — Heat maps of FLEXI RNAs associated with Clusters I-VI that have overlapping binding sites for each of the 47 non-core spliceosomal RBPs with a binding site in ≥30 FLEXIs (columns and rows, respectively). The number of FLEXIs containing overlapping binding sites for each compared pair of RBPs (columns and rows) were log10-transformed, clustered and color-coded. Names of RBPs associated with Clusters I-VI are color coded as shown at the bottom of the Figure. (PDF) [file pgen.1011416.s017.pdf]

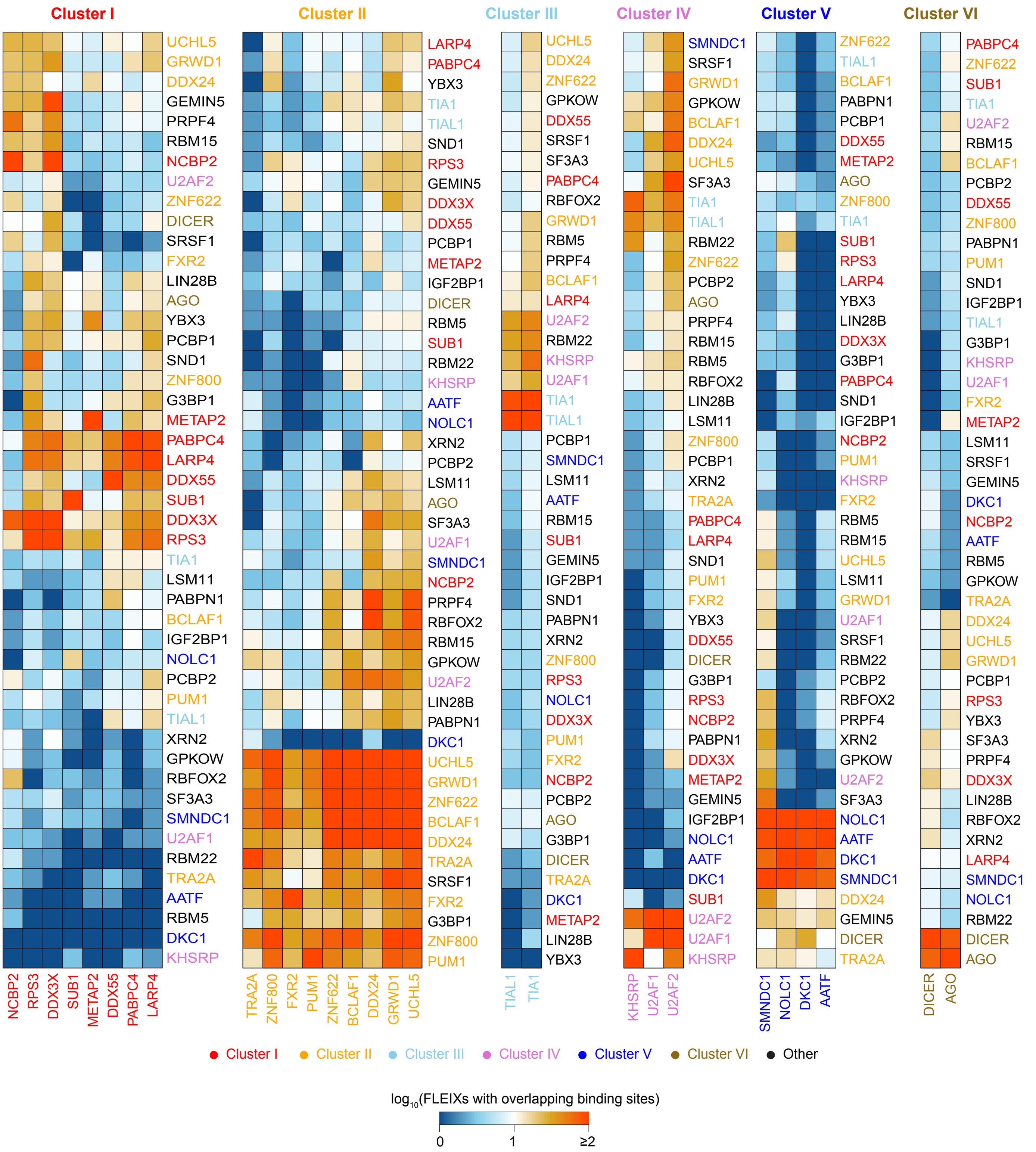

S17 Fig. Patterns of overlapping RBP-binding sites in FLEXI RNAs.
Heat maps of FLEXI RNAs associated with Clusters I-VI that have overlapping binding sites for each of the 47 non-core spliceosomal RBPs with a binding site in ≥30 FLEXIs (columns and rows, respectively). The number of FLEXIs containing overlapping binding sites for each compared pair of RBPs (columns and rows) were $\log_{10}$-transformed, clustered and color-coded. Names of RBPs associated with Clusters I-VI are color coded as shown at the bottom of the Figure.
