# Supplementary material for: Human cells contain myriad excised linear intron RNAs with links to gene regulation and potential utility as biomarkers
Source: PLoS Genet. 2024 Sep 26;20(9):e1011416. doi: 10.1371/journal.pgen.1011416 (PMC11460701; doi:10.1371/journal.pgen.1011416)
Supplement: S18 Fig — UpSet plots showing the distribution of (A) FLEXI RNAs, (B) FLEXI host genes, (C) FLEXI host oncogenes, and (D) FLEXI host tumor suppressor genes in MCF7 cells and MDA-MB-231 cells, compared to those in a combined dataset (Other) for HEK-293T, HeLa S4, K-562 and UHRR cellular RNA samples. FLEXI RNAs from known oncogenes [83] or tumor suppressor genes [84] that were detected only in MCF7 and/or MDA-MB-231 are listed below the plots. ATF3 and KLF4 were annotated as both oncogenes and tumor suppressor genes [83, 84]. (PDF) [file pgen.1011416.s018.pdf]

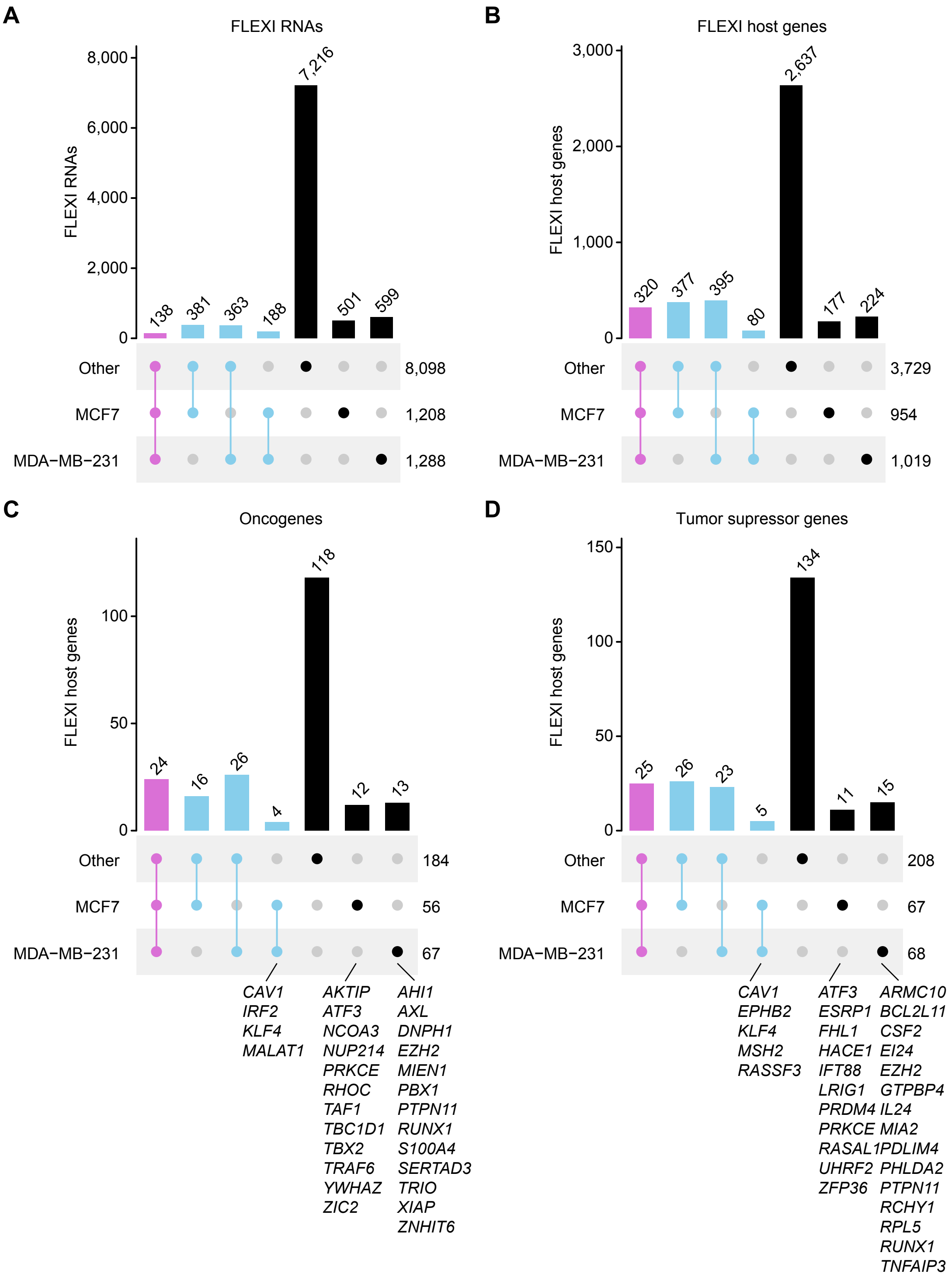

S18 Fig. Cell-type specific FLEXI RNAs and their host genes in MCF7 and MDA-MB-231 cancer cell lines.

UpSet plots showing the distribution of **(A)** FLEXI RNAs, **(B)** FLEXI host genes, **(C)** FLEXI host oncogenes, and **(D)** FLEXI host tumor suppressor genes in MCF7 cells and MDA-MB-231 cells, compared to those in a combined dataset (Other) for HEK-293T, HeLa S4, K-562 and UHRR cellular RNA samples. FLEXI RNAs from known oncogenes (80) or tumor suppressor genes (81) that were detected only in MCF7 and/or MDA-MB-231 are listed below the plots. ATF3 and KLF4 were annotated as both oncogenes and tumor suppressor genes (80, 81).
