# Supplementary material for: Human cells contain myriad excised linear intron RNAs with links to gene regulation and potential utility as biomarkers
Source: PLoS Genet. 2024 Sep 26;20(9):e1011416. doi: 10.1371/journal.pgen.1011416 (PMC11460701; doi:10.1371/journal.pgen.1011416)
Supplement: S19 Fig — Scatter plots comparing enrichment of all and subtypes of cellular RNAs in cytoplasmic and nuclear fractions from HeLa S3, K-562, MDA-MB-231, and MCF7 cells. RNA subtypes are color coded as shown to the right of the scatter plots. Mitochondrial RNAs, tRNAs, Vault RNAs, Y RNA, and 7SL RNA were enriched in the cytoplasmic RNA fraction, while 7SK RNA and snRNAs were enriched in the nuclear RNA fraction. Most snoRNAs (357 to 457 in different cell types) had higher normalized counts in nuclear fractions than in cytoplasmic fractions with only 20–49 snoRNAs having higher normalized counts in cytoplasmic fractions. (PDF) [file pgen.1011416.s019.pdf]

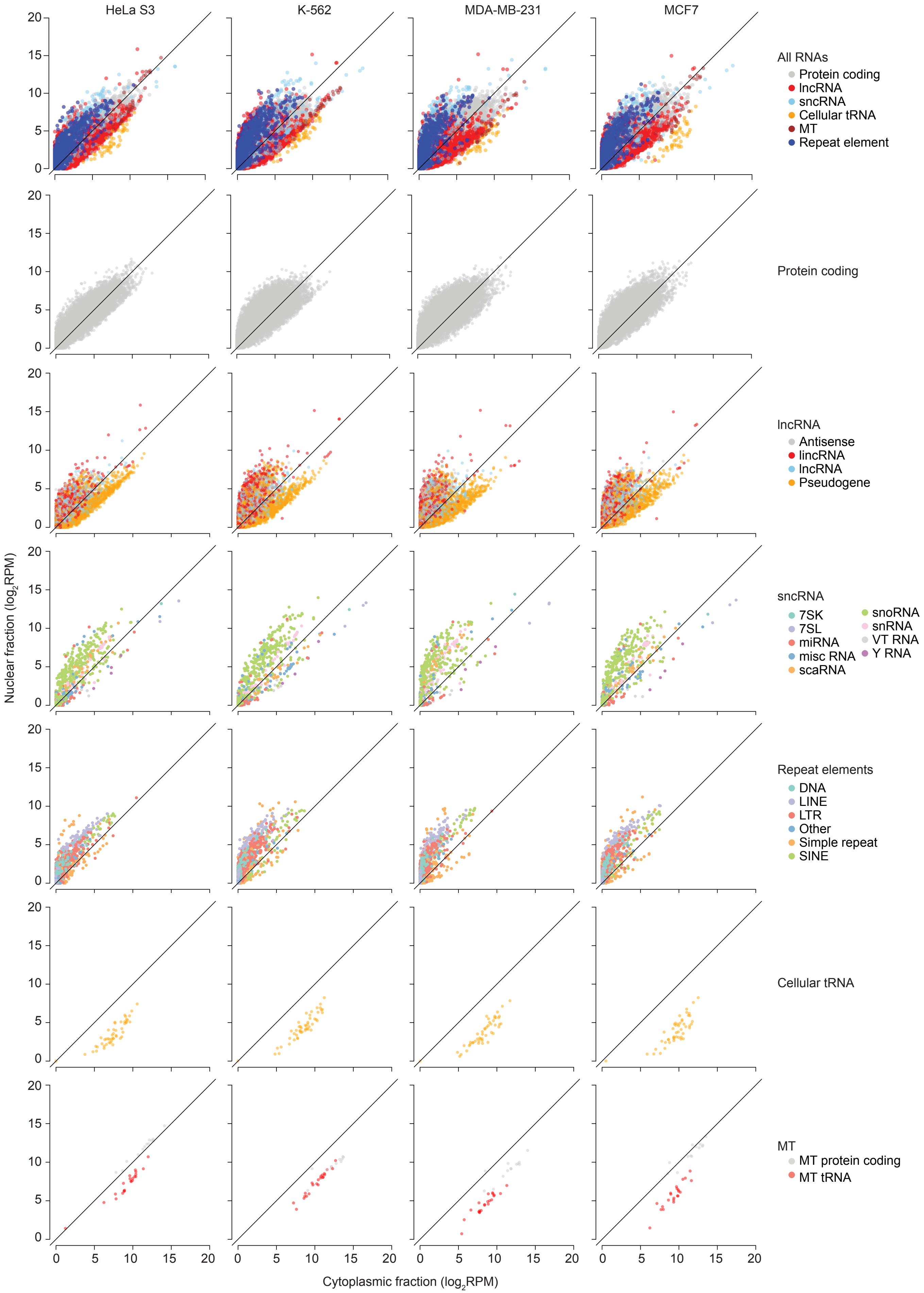

HeLa S3
K-562
MDA-MB-231
MCF7
All RNAs
Protein coding
lncRNA
sncRNA
Cellular tRNA
MT
Repeat element
Protein coding
lncRNA
Antisense
lincRNA
lncRNA
Pseudogene
sncRNA
7SK
7SL
miRNA
misc RNA
scaRNA
snoRNA
snRNA
VT RNA
Y RNA
Repeat elements
DNA
LINE
LTR
Other
Simple repeat
SINE
Cellular tRNA
MT
MT protein coding
MT tRNA
Nuclear fraction (log2RPM)
Cytoplasmic fraction (log2RPM)
0
5
10
15
20

S19 Fig. Analysis of RNAs in nuclear and cytoplasmic fractions from cultured cells.
Scatter plots comparing enrichment of all and subtypes of cellular RNAs in cytoplasmic and nuclear fractions from HeLa S3, K-562, MDA-MB-231, and MCF7 cells. RNA subtypes are color coded as shown to the right of the scatter plots. Mitochondrial RNAs, tRNAs, Vault RNAs, Y RNA, and 7SL RNA were enriched in the cytoplasmic RNA fraction, while 7SK RNA and snRNAs were enriched in the nuclear RNA fraction. Most snoRNAs (357 to 457 in different cell types) had higher normalized counts in nuclear fractions than in cytoplasmic fractions with only 20-49 snoRNAs having higher normalized counts in cytoplasmic fractions.
