# Supplementary material for: Human cells contain myriad excised linear intron RNAs with links to gene regulation and potential utility as biomarkers
Source: PLoS Genet. 2024 Sep 26;20(9):e1011416. doi: 10.1371/journal.pgen.1011416 (PMC11460701; doi:10.1371/journal.pgen.1011416)
Supplement: S20 Fig — (A) and (B) Principal Component Analysis (PCA) plots for RNAs detected by TGIRT-seq in whole-cell (squares), nuclear fractions (Nucleus; triangles), and cytoplasmic fractions (Cytoplasm; circles) for HeLa S3, K-562, MCF7 and MDA-MB-231 cells color coded as shown in the Figure. (C) Heatmap of Euclidean distance between datasets shaded as being more (darker blue) or less (lighter blue) similar as shown by color scale in the Figure. (PDF) [file pgen.1011416.s020.pdf]

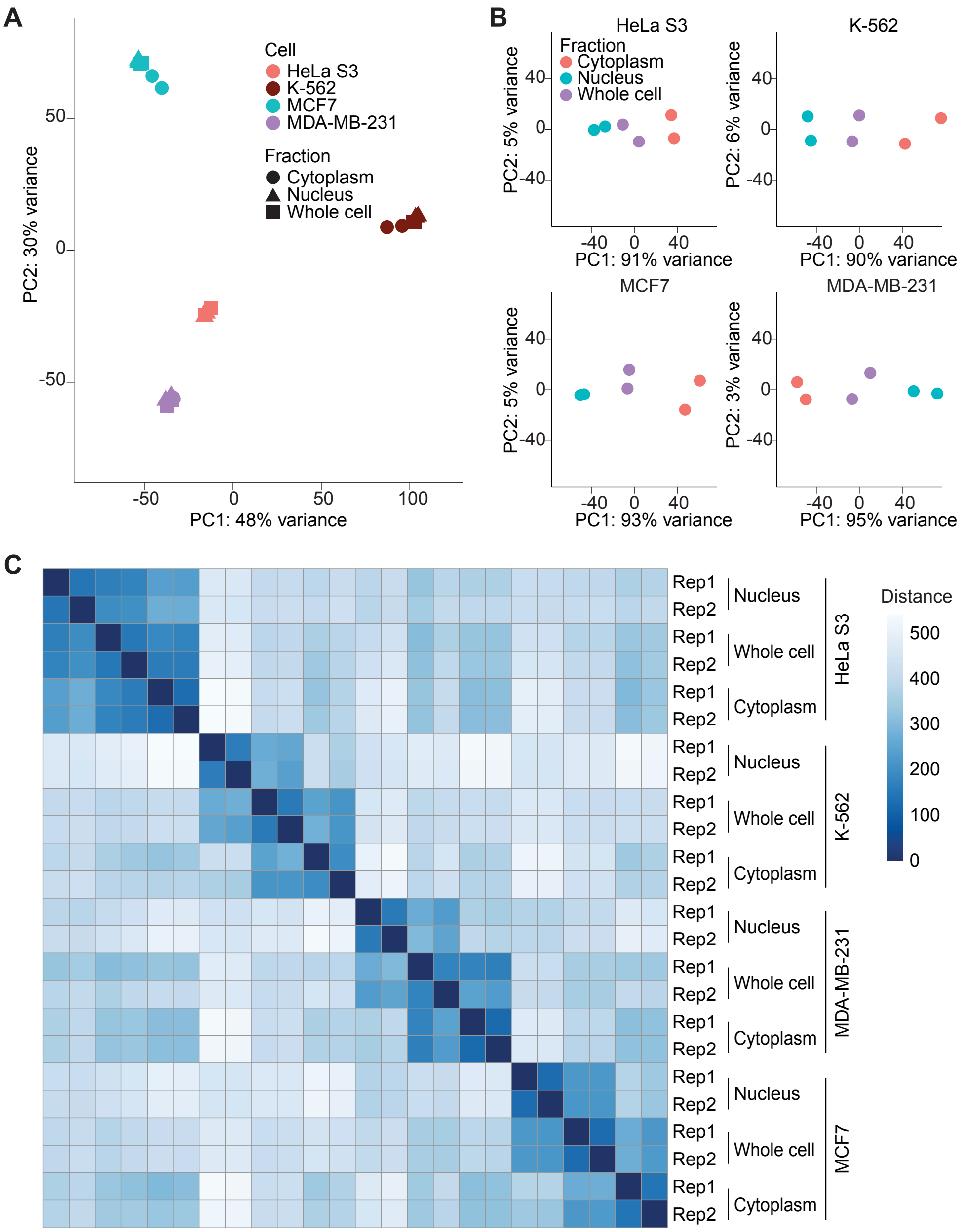

S20 Fig. Clustering analysis of RNAs in nuclear and cytoplasmic fractions from cultured cells.

(A) and **(B)** Principal Component Analysis (PCA) plots for RNAs detected by TGIRT-seq in whole-cell (squares), nuclear fractions (Nucleus; triangles), and cytoplasmic fractions (Cytoplasm; circles) for HeLa S3, K-562, MCF7 and MDA-MB-231 cells color coded as shown in the Figure. **(C)** Heatmap of Euclidean distance between datasets shaded as being more (darker blue) or less (lighter blue) similar as shown by color scale in the Figure.
