# Supplementary material for: Human cells contain myriad excised linear intron RNAs with links to gene regulation and potential utility as biomarkers
Source: PLoS Genet. 2024 Sep 26;20(9):e1011416. doi: 10.1371/journal.pgen.1011416 (PMC11460701; doi:10.1371/journal.pgen.1011416)
Supplement: S21 Fig — (A) Numbers and percentages of host genes encoding mitochondrial proteins annotated in MitoCarta3.0 or cytoplasmic or mitochondrial ribosomal proteins annotated in GRCh38 that contain FLEXIs (top), other short introns (middle), or long introns (bottom) with binding sites for Cluster I RBPs. (B) Numbers and percentages of all host genes for different subsets of introns compared to those for all GRCh38 annotated protein-coding genes. (C) Density plots showing the average coverage of RBP-binding sites across the gene body (Exons CDS + UTRs; black) or intron body (red) calculated using Picard tools normalized by percent length. In the gene body plots, 0% and 100% indicate the 5’ and 3’ ends the mRNAs, respectively. In the intron body plots, 0% indicates the 5’ end of the first intron and 100% indicates the 3’ end of the last intron of the same gene. (PDF) [file pgen.1011416.s021.pdf]

A

FLEXIs

Host genes / RBP	Total	MitoCarta3.0	Cyto. ribosomal proteins	Mito. ribosomal proteins
LARP4	45	10 (22.2%)	15 (33.3%)	1 (2.2%)
PABPC4	54	8 (14.8%)	23 (42.6%)	1 (1.9%)
SUB1	26	3 (11.5%)	8 (30.8%)	0 (0.0%)
DDX3X	137	35 (25.5%)	14 (10.2%)	9 (6.6%)
RPS3	99	14 (14.1%)	10 (10.1%)	2 (2.0%)
NCBP2	36	3 (8.3%)	3 (8.3%)	0 (0.0%)
DDX55	28	4 (14.3%)	4 (14.3%)	0 (0.0%)
METAP2	48	4 (8.3%)	7 (14.6%)	0 (0.0%)

Other short introns

Host genes / RBP	Total	MitoCarta3.0	Cyto. ribosomal proteins	Mito. ribosomal proteins
LARP4	132	25 (18.9%)	18 (13.6%)	4 (3.0%)
PABPC4	112	15 (13.4%)	25 (22.3%)	0 (0.0%)
SUB1	57	6 (10.5%)	9 (15.8%)	0 (0.0%)
DDX3X	510	83 (16.3%)	23 (4.5%)	11 (2.2%)
RPS3	223	28 (12.6%)	16 (7.2%)	3 (1.3%)
NCBP2	170	19 (11.2%)	6 (3.5%)	0 (0.0%)
DDX55	87	15 (17.2%)	3 (3.4%)	0 (0.0%)
METAP2	96	7 (7.3%)	8 (8.3%)	0 (0.0%)

Long introns

Host genes / RBP	Total	MitoCarta3.0	Cyto. ribosomal proteins	Mito. ribosomal proteins
LARP4	801	118 (14.7%)	49 (6.1%)	14 (1.7%)
PABPC4	661	86 (13.0%)	68 (10.3%)	9 (1.4%)
SUB1	464	62 (13.4%)	31 (6.7%)	5 (1.1%)
DDX3X	3,107	354 (11.4%)	47 (1.5%)	34 (1.1%)
RPS3	872	112 (12.8%)	35 (4.0%)	4 (0.5%)
NCBP2	1,554	133 (8.6%)	20 (1.3%)	3 (0.2%)
DDX55	410	44 (10.7%)	11 (2.7%)	3 (0.7%)
METAP2	445	39 (8.8%)	28 (6.3%)	2 (0.4%)

B

Host genes	Total	MitoCarta3.0	Cyto. ribosomal proteins	Mito. ribosomal proteins
Cluster I FLEXIs	324	59 (18.2%)	31 (9.6%)	10 (3.1%)
Cluster V FLEXIs	116	8 (6.9%)	13 (11.2%)	0 (0.0%)
All FLEXIs	3,729	280 (7.5%)	60 (1.6%)	23 (0.6%)
Other short introns	5,051	399 (7.9%)	74 (1.5%)	29 (0.6%)
Long introns	13,166	970 (7.4%)	95 (0.7%)	73 (0.6%)
GRCh38 genes	19,780	1,122 (5.7%)	101 (0.5%)	78 (0.4%)

C

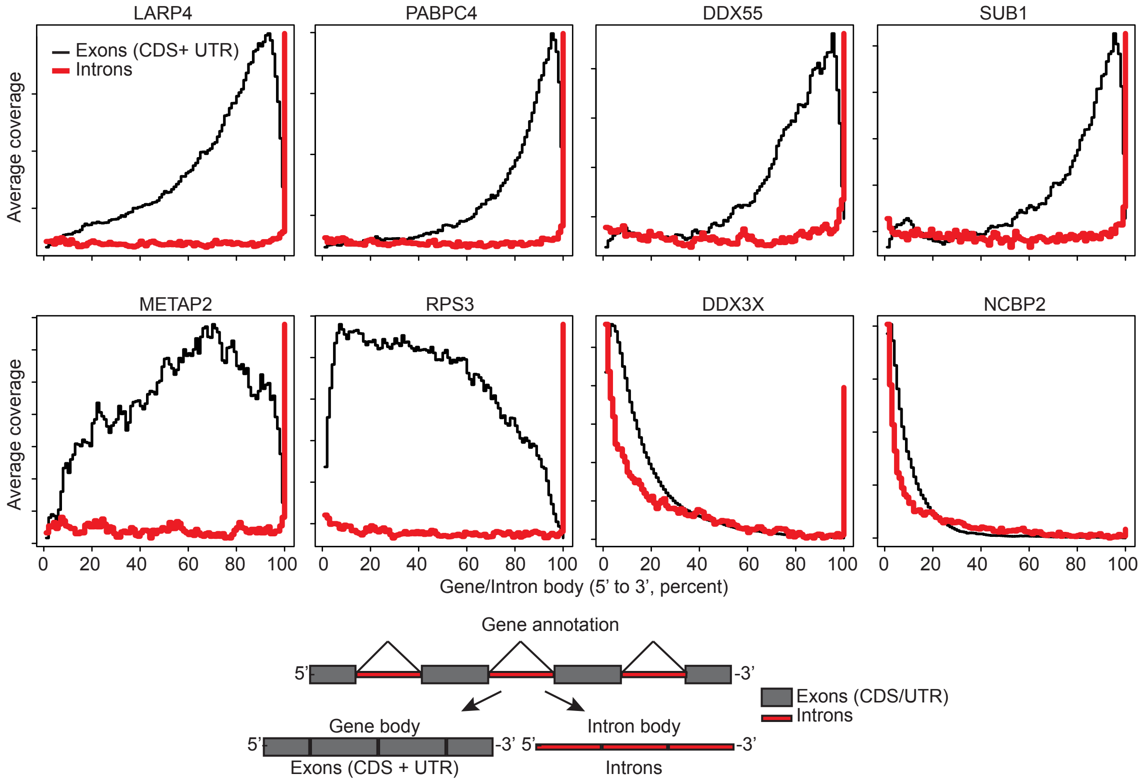

S21 Fig. Characterization of RBP-binding sites in intron RNAs encoded by different categories of host genes.

(A) Numbers and percentages of host genes encoding mitochondrial proteins annotated in MitoCarta3.0 or cytoplasmic or mitochondrial ribosomal proteins annotated in GRCh38 that contain FLEXIs (top), other short introns (middle), or long introns (bottom) with binding sites for Cluster I RBPs. **(B)** Numbers and percentages of all host genes for different subsets of introns compared to those for all GRCh38 annotated protein-coding genes. **(C)** Density plots showing the average coverage of RBP-binding sites across the gene body (Exons CDS + UTRs; black) or intron body (red) calculated using Picard tools normalized by percent length. In the gene body plots, 0% and 100% indicate the 5' and 3' ends the mRNAs, respectively. In the intron body plots, 0% indicates the 5' end of the first intron and 100% indicates the 3' end of the last intron of the same gene.
