# Supplementary material for: Human cells contain myriad excised linear intron RNAs with links to gene regulation and potential utility as biomarkers
Source: PLoS Genet. 2024 Sep 26;20(9):e1011416. doi: 10.1371/journal.pgen.1011416 (PMC11460701; doi:10.1371/journal.pgen.1011416)
Supplement: S1 Table — (PDF) [file pgen.1011416.s022.pdf]

S1 Table. Summary of TGIRT-seq datasets obtained in this study.

Cell / Tissue	Raw reads ($x10^6$)	Trimmed reads ($x10^6$)	Mapped reads ($x10^6$)	Mapped to feature ($x10^6$)
HEK-293T (Bio. 1)*	741.6	726.5 (98.0%)	715.2 (98.5%)	690.9 (96.6%)
HeLa S3†	851.0	803.3 (94.4%)	768.4 (95.7%)	705.6 (92.0%)
K-562 (Bio. 1)§	744.2	725.0 (97.4%)	713.8 (98.4%)	698.5 (97.9%)
UHRR (Bio. 1)‡	712.0	682.2 (95.8%)	666.3 (97.7%)	630.9 (94.7%)
Plasma¶	134.5	122.7 (91.3%)	71.1 (57.9%)	61.7 (87.2%)
MCF7‖	757.8	703.7 (92.9%)	692.1 (98.4%)	673.7 (97.3%)
MDA-MB-231 (Bio. 1)#	226.1	211.3 (93.5%)	207.5 (98.2%)	202.2 (97.5%)
HEK-293T (Bio. 2)‡‡	224.1	211.0 (94.2%)	180.8 (85.7%)	162.6 (89.9%)
K-562 (Bio. 2) ¶¶	59.4	54.6 (91.9%)	47.9 (87.7%)	45.5 (95.1%)
MDA-MB-231 (Bio. 2) ##	314.8	274.3 (87.1%)	259.1 (94.5%)	243.7 (94.1%)
UHRR (Bio. 2)§§	416.4	397.3 (95.4%)	359.4 (90.5%)	293.0 (81.5%)
HeLa S3 (Total RNA) **	46.8	27.2 (58.2%)	20.5 (75.1%)	13.3 (65.0%)
HeLa S3 (Nucleus RNA) **	37.4	27.5 (73.5%)	22.4 (81.4%)	15.3 (68.5%)
HeLa S3 (Cytoplasm RNA) **	49.7	29.3 (59.1%)	21.1 (71.9%)	13.2 (62.7%)
K-562 (Total RNA) **	66.2	49.7 (75.1%)	37.5 (75.4%)	24.3 (64.8%)
K-562 (Nucleus RNA) **	75.5	66.7 (88.4%)	56.5 (84.6%)	34.4 (60.9%)
K-562 (Cytoplasm RNA) **	78.4	59.4 (75.8%)	40.9 (68.8%)	25.4 (62.0%)
MDA-MB-231 (Total RNA) **	82.0	74.1 (90.3%)	57.4 (77.5%)	38.7 (67.3%)
MDA-MB-231 (Nucleus RNA) **	108.6	102.3 (94.2%)	87.7 (85.7%)	54.3 (61.9%)

MDA-MB-231 (Cytoplasm RNA) **	108.1	92.6 (85.7%)	65.2 (70.4%)	42.0 (64.4%)
MCF7 (Total RNA) **	70.7	63.8 (90.2%)	49.3 (77.3%)	33.3 (67.6%)
MCF7 (Nucleus RNA) **	89.7	85.0 (94.8%)	73.7 (86.7%)	48.2 (65.5%)
MCF7 (Cytoplasm RNA) **	109.9	94.9 (86.3%)	68.3 (72.0%)	43.6 (63.9%)

[*] HEK-293T cell RNA, combined datasets from 8 technical replicates (SRA BioProject accession number PRJNA648481, HEK-rep1 to 8).

[†] HeLa S3 cell RNA, combined datasets from 10 technical replicates (SRA BioProject accession number PRJNA648481, HeLa-rep1 to 10).

[§] K-562 cell RNA, combined datasets from 8 technical replicates (SRA BioProject accession number PRJNA648481, K-562-rep1 to 8).

[‡] Universal human reference RNA, combined datasets from 8 technical replicates (SRA BioProject accession number PRJNA648481, UHRR-rep1 to 8, lot# 0006390163).

[¶] Commercial human plasma pooled plasma from healthy individuals. Fifteen combined datasets (SRA BioProject accession number PRJNA640428, samples DNaseI_1 to 12, ExoI_1 to 3) (Yao *et al.* 2020).

[||] MCF7 RNA, combined datasets from 8 technical replicates (SRA BioProject accession number PRJNA648481, MCF-rep1 to 8).

[#] MDA-MB-231 RNA, combined datasets from 2 technical replicates (SRA BioProject accession number PRJNA648481, MDA-rep1 and 2).

[‡‡] HEK-293T cell RNA, 2 technical replicates (SRA BioProject accession number PRJNA389469, samples WC and YC) (Shurtleff *et al.*, 2017).

[¶¶] K-562 cell RNA, Bio. 2, 7 technical replicates (SRA BioProject accession number PRJNA722757, samples UFC1 to 7).

[##] MDA-MB-231 cell RNA, Bio. 2, 5 technical replicates (SRA BioProject accession number PRJNA722757, samples MDA1 to 5).

[§§] Universal human reference RNA, 10 technical replicates (SRA BioProject accession number PRJNA722757, samples UHRR0 to 9, lot# 0006227794).

[**] Total cellular, nucleus and cytoplasm RNA from 4 cell lines, 2 technical replicates each (SRA BioProject accession number PRJNA648481, samples SRR26807958-81).
