# Supplementary material for: Human cells contain myriad excised linear intron RNAs with links to gene regulation and potential utility as biomarkers
Source: PLoS Genet. 2024 Sep 26;20(9):e1011416. doi: 10.1371/journal.pgen.1011416 (PMC11460701; doi:10.1371/journal.pgen.1011416)
Supplement: S4 Table — (PDF) [file pgen.1011416.s025.pdf]

S4 Table. Abundance of sncRNAs (RPM) detected by TGIRT-seq in cellular RNA samples compared to literature copy number per cell values for these RNAs.

Name	RPM				Copies/cell*	Type
	K-562	HEK-293T	HeLa S3	UHRR		
U1	1,385	1,266	1,337	2,647	$1x10^6$	Major spliceosomal snRNA
U2	5,764	9,476	28,774	9,787	$5x10^5$	Major spliceosomal snRNA
U4	65	34	540	76	$2x10^5$	Major spliceosomal snRNA
U5	1,146	2,703	13,241	2,715	$2x10^5$	Major spliceosomal snRNA<br>Minor spliceosomal snRNA
U6	974	1,766	923	1,355	$4x10^5$	Major spliceosomal snRNA
U7	3	4	49	3	$4x10^3$	U7
U11	2	3	11	8	$1x10^4$	Minor spliceosomal snRNA
U12	36	24	192	41	$5x10^3$	Minor spliceosomal snRNA
U4ATAC	37	21	1,291	32	$2x10^3$	Minor spliceosomal snRNA
U6ATAC	204	286	699	268	$2x10^3$	Minor spliceosomal snRNA
RN7SK	1,368	2,771	5,043	7,174	$2x10^5$	7SK
RN7SL	3,445	8,080	4,689	19,801	$5x10^5$	7SL
RPPH1	165	245	257	1,293	$2x10^5$	RNase P RNA component
SNORD3	7,637	12,700	23,204	38,531	$2x10^5$	C/D box snoRNA, U3
SNORD118	222	985	552	745	$4x10^4$	C/D box snoRNA, U8
SNORD13	436	367	817	1,223	$1x10^4$	C/D box snoRNA, U13
SNORD14	4,278	3,887	9,979	11,073	$1x10^4$	C/D box snoRNA, U14
SNORD22	362	240	748	947	$1x10^4$	C/D box snoRNA, U22
RMRP	669	1,776	2,211	3,276	$1x10^5$	MRP

*Copy number per cell values for sncRNAs in vertebrate cells (Tycowski *et al.*, 2006) used in the linear regression analysis of Fig. S6.

Tycowski K, Kolev N, Conrad N, Fok V, Steitz J. The ever-growing world of small nuclear ribonucleoproteins. In: Gesteland R, Cech T, Atkins J, editors. The RNA World. Third Edition ed. New York: Cold Spring Harbor Laboratory Press; 2006. p. 327-368.
