# Supplementary material for: Human cells contain myriad excised linear intron RNAs with links to gene regulation and potential utility as biomarkers
Source: PLoS Genet. 2024 Sep 26;20(9):e1011416. doi: 10.1371/journal.pgen.1011416 (PMC11460701; doi:10.1371/journal.pgen.1011416)
Supplement: S6 Table — (PDF) [file pgen.1011416.s027.pdf]

S6 Table. Cellular functions of RBPs with binding sites for ≥30 different FLEXIs.

Symbol	Name	Function
AATF	Apoptosis antagonizing transcription factor	Transcriptional cofactor with roles in cell proliferation, apoptosis, DNA damage response and general stress response through regulation of Rb, HDAC1, and p53 functions; snoRNA processing.
AGO	Argonaute	Central player in RNA silencing processes, essential component of RNA-induced silencing complex (RISC).
AKAP8L	A-kinase anchoring protein 8 like	mRNA splicing, unspliced mRNA export.
AQR	RNA helicase aquarius	Spliceosome component. Pre-mRNA splicing and snoRNP biogenesis.
BCLAF1	BCL2-2-associated transcription factor 1	Transcriptional repressor; promotes apoptosis through interaction with BCL2; Upregulated in senescence, promotes p53 transcription in response to DNA damage.
BUD13	BUD13 homolog	Spliceosome component. Pre-mRNA splicing.
DDX24	ATP-dependent RNA helicase DDX24	ATP-dependent RNA helicase and negative regulator of p53.
DDX3X	ATP-dependent RNA helicase DDX3X	Multifunctional ATP-dependent RNA helicase with functions in cell cycle control, apoptosis, and innate immunity; critical role in stress granule assembly.
DDX55	ATP-dependent RNA helicase DDX55	ATP-binding RNA helicase.
DICER	DICER	Cleaves double-stranded RNA and pre-miRNA to form siRNA or miRNA.
DKC1	H/ACA ribonucleoprotein complex subunit DKC1	Catalytic subunit of H/ACA small nucleolar ribonucleoprotein (H/ACA snoRNP) complex; plays an active role in telomerase stabilization.
EFTUD2	116 kDa U5 small nuclear ribonucleoprotein component	Spliceosome component. Pre-mRNA splicing.
FXR2	Fragile X mental retardation syndrome-related protein 2	RNA-binding protein.

G3BP1	Ras GTPase-activating protein-binding protein 1	ATP- and Mg-dependent helicase that plays an essential role in innate immunity; Also functions in stress granule assembly and is associated with cellular senescence. Regulates Ras, TGF-β/Smad, Src/FAK and p53 signaling pathways.
GEMIN5	Gem-associated protein 5	Plays a role in cytoplasmic assembly of snRNPs; component of the SMN complex which plays a role in alternative splicing.
GPKOW	G-patch domain and KOW motifs-containing protein	Component of the spliceosome.
GRSF1	G-rich RNA sequence binding factor 1	Alternative splicing, mRNA export.
GRWD1	Glutamate-rich WD repeat-containing protein 1	Histone binding-protein that regulates chromatin dynamics and minichromosome maintenance (MCM) loading at replication origins; negatively regulates p53.
HNRNPA1	Heterogeneous nuclear ribonucleoprotein A1	Packaging of pre-mRNA into hnRNP. Transport poly(A) mRNA from the nucleus to the cytoplasm. Translational inhibition by binding to IRES. Binds specific miRNA hairpins. Plays role in HCV RNA replication.
HNRNPC	Heterogeneous nuclear ribonucleoproteins C1/C2	Packaging of pre-mRNA into hnRNP. Interacts with poly-U tracts in the 3'/5' UTR and modulates mRNA stability and translation. Splicing regulation.
HNRNPUL1	Heterogeneous nuclear ribonucleoprotein U-like protein 1	Transcriptional regulator. mRNA processing and transport.
IGF2BP1	Insulin-like growth factor 2 mRNA-binding protein 1	RNA-binding protein that recruits target transcripts to cytoplasmic protein-RNA complexes (mRNPs); Promotes cell cycle progression through regulation of E2F translation.
ILF3	Interleukin enhancer-binding factor 3	circRNAs processing in the nucleus. Innate anti-viral response.
KHDRBS1	KH domain-containing, RNA-binding, signal transduction-associated protein 1	mRNA nuclear export, stability, and translation regulation. Involved in alternative splicing.
KHSRP	KH-type splicing regulatory protein	Regulates alternative pre-mRNA splicing, mRNA degradation, trafficking and localization.
LARP4	La-related protein 4	RNA binding protein that binds to the polyA tract of mRNA molecules

LIN28B	Lin-28 homolog B	Suppressor of miRNA biogenesis, specifically let7; inhibits apoptosis
LSM11	U7 snRNA-associated Sm-like protein LSm11	Component of the U7 snRNP complex that is involved in the histone 3'-end pre-mRNA processing
METAP2	Methionine aminopeptidase 2	Co-translationally removes the N-terminal methionine from nascent proteins.
NCBP2	Nuclear cap-binding protein subunit 2	Component of cap-binding complex that binds cap of pre-mRNAs; involved in splicing, translation regulation and NMD
NOLC1	Nucleolar and coiled-body phosphoprotein 1	Nucleolar protein that plays a critical role in snoRNP assembly and acts as a regulator of RNA polymerase I by connecting RNA polymerase I with enzymes responsible for ribosomal processing and modification; Stabilizes telomeres by regulating TRF2 retention.
NONO	Non-POU domain-containing octamer-binding protein	Pre-mRNA splicing. Involved in formation of nuclear paraspeckles, DNA non-homologous end joining (NHEJ) for double strand repair and V(D)J recombination. Transcriptional regulation. Anti-viral innate response.
PABPC4	Polyadenylate-binding protein 4	Binds the polyA tail of mRNA
PABPN1	Polyadenylate-binding protein 2	Involved in the 3'-end formation of mRNA precursors (pre-mRNA) by the addition of a poly(A) tail; Regulated by ATM and plays a crucial role in DSB repair.
PCBP1	Poly(rC)-binding protein 1	Binds single stranded nucleic acids; regulates antiviral response mediated by MAVS signaling
PCBP2	Poly(rC)-binding protein 2	Binds single stranded nucleic acids; regulates antiviral response mediated by MAVS signaling
PRPF4	Pre-mRNA processing factor 4	Component of the U4/U6-U5 tri-snRNP complex that is involved in pre-mRNA splicing.
PRPF8	Pre-mRNA-processing-splicing factor 8	Spliceosome component. Pre-mRNA splicing.
PUM1	Pumilio RNA binding family member 1	Post-transcriptional repressor; binds the 3'-UTR of mRNA targets; regulates MIR199 expression.
QKI	KH domain-containing RNA-binding protein QKI	RNA reader protein, which recognizes and binds specific RNAs, thereby regulating RNA metabolic processes, such as pre-mRNA splicing, circular RNA (circRNA) formation, mRNA export, mRNA stability and/or translation.

RBFOX2	RNA binding Fox-1 homolog 2	Regulates alternative splicing by preventing binding of U2AF2 to the 3'-splice site.
RBM15	RNA-binding protein 15	Regulates m6A modification of RNAs; alternative splicing factor
RBM22	RNA-binding protein 22	Required component of the spliceosome
RBM5	RNA-binding protein 5	Component of the spliceosome; alternative splicing factor
RPS3	40S ribosomal protein S3	Role in regulating transcription; implicated in regulating DNA damage response and apoptosis.
SERBP1	SERPINE1 mRNA binding protein 1	Stabilize inactive ribosome, involved in PML-body formation
SF3A3	Splicing factor 3a subunit 3	Required splicing factor
SF3B4	Splicing factor 3B subunit 4	Spliceosome component. Pre-mRNA splicing.
SMNDC1	Survival motor neuron domain containing 1	Involved in spliceosome assembly; snoRNA processing.
SND1	Staphylococcal nuclease and Tudor domain containing 1	Transcriptional co-activator of STAT5 and EB virus nuclear antigen 2; endonuclease that mediates miRNA decay.
SRSF1	Serine/arginine-rich splicing factor 1	Prevents exon skipping, regulates alternative splicing
SRSF7	Serine/arginine-rich splicing factor 7	mRNA splicing and export
SUB1	Activated RNA polymerase II transcriptional coactivator p15	General coactivator that functions cooperatively with TAFs and mediates functional interactions between upstream activators and the general transcriptional machinery; critical role in genome integrity and chromatin compaction, regulates transcription in response to stress.
TIA1	TIA1 cytotoxic granule associated RNA binding protein	Alternative splicing factor, regulation of mRNA translation; potential role in apoptosis
TIAL1	TIA1 cytotoxic granule associated RNA binding protein like 1	Binds AU-rich elements; roles in regulating translation, splicing and apoptosis.
TRA2A	Transformer 2 alpha homolog	Regulates pre-mRNA splicing

U2AF1	U2 small nuclear RNA auxiliary factor 1	Critical role in splicing, recruits U2 snRNP to branch point
U2AF2	U2 small nuclear RNA auxiliary factor 2	Roles in pre-mRNA splicing and 3' end processing
UCHL5	Ubiquitin carboxyl-terminal hydrolase isozyme L5	Protease
UPF1	Regulator of nonsense transcripts 1	NMD and mRNA export
XPO5	Exportin 5	dsRNA and pre-miRNA export
XRN2	5'-3' exoribonuclease 2	May promote transcription termination by RNA polymerase II
YBX3	Y-Box-binding protein 3	Binds also to full-length mRNA and to short RNA sequences containing the consensus site 5'-UCCAUCA-3'.
ZC3H11A	Zinc finger CCCH-type containing 11A	mRNA and viral RNA export
ZNF622	Zinc finger protein 622	May behave as an activator of the bound transcription factor, MYBL2; positive regulator of apoptosis.
ZNF800	Zinc finger protein 800	May be involved in transcriptional regulation.

Functional annotations RBPs in the ENCODE eCLIP dataset were based on Table S2 of Nostrand *et al.* The functional annotations of AGO1-4 and DICER were from the UniProt database (https://www.uniprot.org).

Van Nostrand EL, Pratt GA, Yee BA, Wheeler EC, Blue SM, Mueller J, et al. Principles of RNA processing from analysis of enhanced CLIP maps for 150 RNA binding proteins. Genome Biol. 2020;21(1): 90. doi: 10.1186/s13059-020-01982-9.
