# Supplementary material for: Human cells contain myriad excised linear intron RNAs with links to gene regulation and potential utility as biomarkers
Source: PLoS Genet. 2024 Sep 26;20(9):e1011416. doi: 10.1371/journal.pgen.1011416 (PMC11460701; doi:10.1371/journal.pgen.1011416)
Supplement: S8 Table — (PDF) [file pgen.1011416.s029.pdf]

S8 Table. Oligonucleotides used for construction of TGIRT-seq libraries.

Name	Sequence and notes
NTT R2 RNA	5’-AAGAUCGGAAGAGCACACGUCUGAACUCCAGUCAC/3SpC/
NTT R2R DNA	5’-GTGACTGGAGTTCAGACGTGTGCTCTTCCGATCTTN-3’, where N is an equimolar mix of A, C, G, T (obtained by hand mixing of individual oligonucleotides with A, C, G and T at their 3’ end).
R1R DNA	R1R DNA: 5’-/5Phos/GATCGTCGGACTGTAGAACTCTGAACGTGT AG/3SpC3/. The R1R oligonucleotide was adenylated. as described in Materials and Methods.
Illumina multiplex PCR primer	5’-AATGATACGGCGACCACCGAGATCTACACGTTCAGAGTTCTA CAGTCCGACGATC-3’
Illumina index PCR primer	5’ CAAGCAGAAGACGGCATACGAGAT BARCODE* GTGACTGGA GTTCAGACGTGTGCTCTTCCGATCT-3’, where BARCODE* corresponds to the 6 nucleotide Illumina TruSeq barcode sequence.
