# Supplementary material for: Human cells contain myriad excised linear intron RNAs with links to gene regulation and potential utility as biomarkers
Source: PLoS Genet. 2024 Sep 26;20(9):e1011416. doi: 10.1371/journal.pgen.1011416 (PMC11460701; doi:10.1371/journal.pgen.1011416)
Supplement: S9 Table — (PDF) [file pgen.1011416.s030.pdf]

S9 Table. Oligonucleotides used for ddPCR and exonuclease digestion assays.

Name †	Sequence
1I_H3F3B (Forward primer-FLEXI)	TAAGACAAAGGCCTGGAGCC
1I_H3F3B (RT primer/Reverse primer-FLEXI)	GGACGAGAGCCGCACTATTA
1I_H3F3B (Forward primer-junction)	CCGACCTGAGGTTTCAGAGC
1I_H3F3B (Reverse primer-junction)	GGCTCCAGGCCTTTGTCTTA
3I_RAN (Forward primer-FLEXI)	TGCTGGAAAACCTTGCTTGTG
3I_RAN (RT primer/Reverse primer-FLEXI)	CTGAAACGGAAACGATGCGA
3I_RAN (Forward primer-junction)	GGTTGGTGATGGTGGTACTG
3I_RAN (Reverse primer-junction)	CACAAGCAAGGTTTTCCAGCA
7I_EIF4A1 (Forward primer-FLEXI)	CCTGGTAGTGAGTTGTTGGGT
7I_EIF4A1 (RT primer/Reverse primer-FLEXI)	TGGAGGTTGGGGGACAAAAAT
7I_EIF4A1 (Forward primer-junction)	TTGACCCTGGAGGGTATCCG
7I_EIF4A1 (Reverse primer-junction)	AACAACTCACTACCAGGCCC
4I_JUP (Forward primer-FLEXI)	GGAGGCTGGGTCCCACT
4I_JUP (RT primer)	AGAGGAAAGGAAAAGCCAGGT
4I_JUP (Reverse primer-FLEXI)	CCAGGTTAAACGTCGGCCAG
4I_JUP (Forward primer-junction)	CACTCATGGAGTTGCTGCAC
4I_JUP (Reverse primer-junction)	AGTGGGACCCAGCCTCC
16I_POLG (Forward primer-FLEXI)	TAGCCAGGCCTTGGGTGG
16I_POLG (RT primer/Reverse primer-FLEXI)	CAGAGCCAGTCCACTAGGGC
16I_POLG (Forward primer-junction)	GATCTGGCCAATGATGCCTG
16I_POLG (Reverse primer-junction)	CAGTCCCCTGCCTAGATCCT
2I_ACTB (Forward primer-FLEXI)	GTGTCTTTCCTGCCTGAGCTG
2I_ACTB (RT primer/Reverse primer-FLEXI)	TGAGGAGGGAAGGGGACAGG
2I_ACTB (Forward primer-junction)	GAAGGAGATCACTGCCCTGG
2I_ACTB (Reverse primer-junction)	CAGCTCAGGCAGGAAAGACA
1I_RPS2 (Forward primer-FLEXI)	GTAGGCTGGTGGCAGGTGAT
1I_RPS2 (RT primer/Reverse primer-FLEXI)	CTGGTGAGGGAAGGAGTCAG
1I_RPS2 (Forward primer-junction)	GAGGCTACTGGGGGAACAAG
1I_RPS2 (Reverse primer-junction)	ATCACCTGCCACCAGCCTAC
U6 (Forward primer)	CGCTTCGGCAGCACATATAC
U6 (RT primer/Reverse primer)	ATTTGCGTGTCATCCTTGCG
U7 (Forward primer)	TACAGCTCTTTTAGAATTTGTCTAGTAGGC
U7 (RT primer/Reverse primer)	GGGGCTTTCCGGTAAAAAGCC
SNORD14B (Forward primer)*	CACTGTGATGATGGTTTTCCAAC
SNORD14B (RT primer/Reverse primer)*	AGGAAGGTTTACCCAACACTAAG
SNORD44 (Forward primer)	GATAAGCAAATGCTGACTGAACA
SNORD44 (RT primer/Reverse primer)	AGTCAGTTAGAGCTAATTAAGACC

†Primers are named according to FLEXI intron number and gene name or sncRNA name.

* Dodd DW, Gagnon KT, Corey DR. Digital quantitation of potential therapeutic target RNAs. Nucleic Acid Ther. 2013;23(3): 188-194. doi: 10.1089/nat.2013.0427.
