# Supplementary material for: Human cells contain myriad excised linear intron RNAs with links to gene regulation and potential utility as biomarkers
Source: PLoS Genet. 2024 Sep 26;20(9):e1011416. doi: 10.1371/journal.pgen.1011416 (PMC11460701; doi:10.1371/journal.pgen.1011416)
Supplement: S4 Fig — (A) 5’- and 3’-splice sites (5’SS and 3’SS, respectively) and branch-point (BP) consensus sequences of GU-AG and AU-AC U12-type FLEXIs in a combined datasets for the 4 cellular RNA samples (HEK-293T, HeLa S3, K-562, and UHRR) compared to literature consensus sequences for human U12-type introns (top) [28, 30] and consensus sequences for other U12-type short (≤300 nt) and long (>300 nt) introns in the same combined datasets. The number of introns for each consensus sequence is indicated to the right of the sequence. (B) IGV screenshots showing read alignments for U12-type and non-canonical splice-site FLEXI RNAs in RNA samples from different cell lines. FLEXIs are named at the top above an arrow indicating the 5’ to 3’ orientation of the RNA with the tracks below showing gene annotations (exons, thick lines; introns, thin lines) followed by alignments of FLEXI reads in different cellular RNA samples color coded by sample type. The length of the intron that is the focus of each panel is shown above the read alignments, and the fraction of FLEXI reads (continuous reads that began and ended within 3 nt of the annotated 5’- and 3’-splice sites) for that intron in different cell lines is shown within the read alignments. Reads were down sampled to a maximum of 100 for display in IGV. NTA, non-templated nucleotides added to the 3’ end of cDNAs during TGIRT-seq library preparation. (PDF) [file pgen.1011416.s004.pdf]

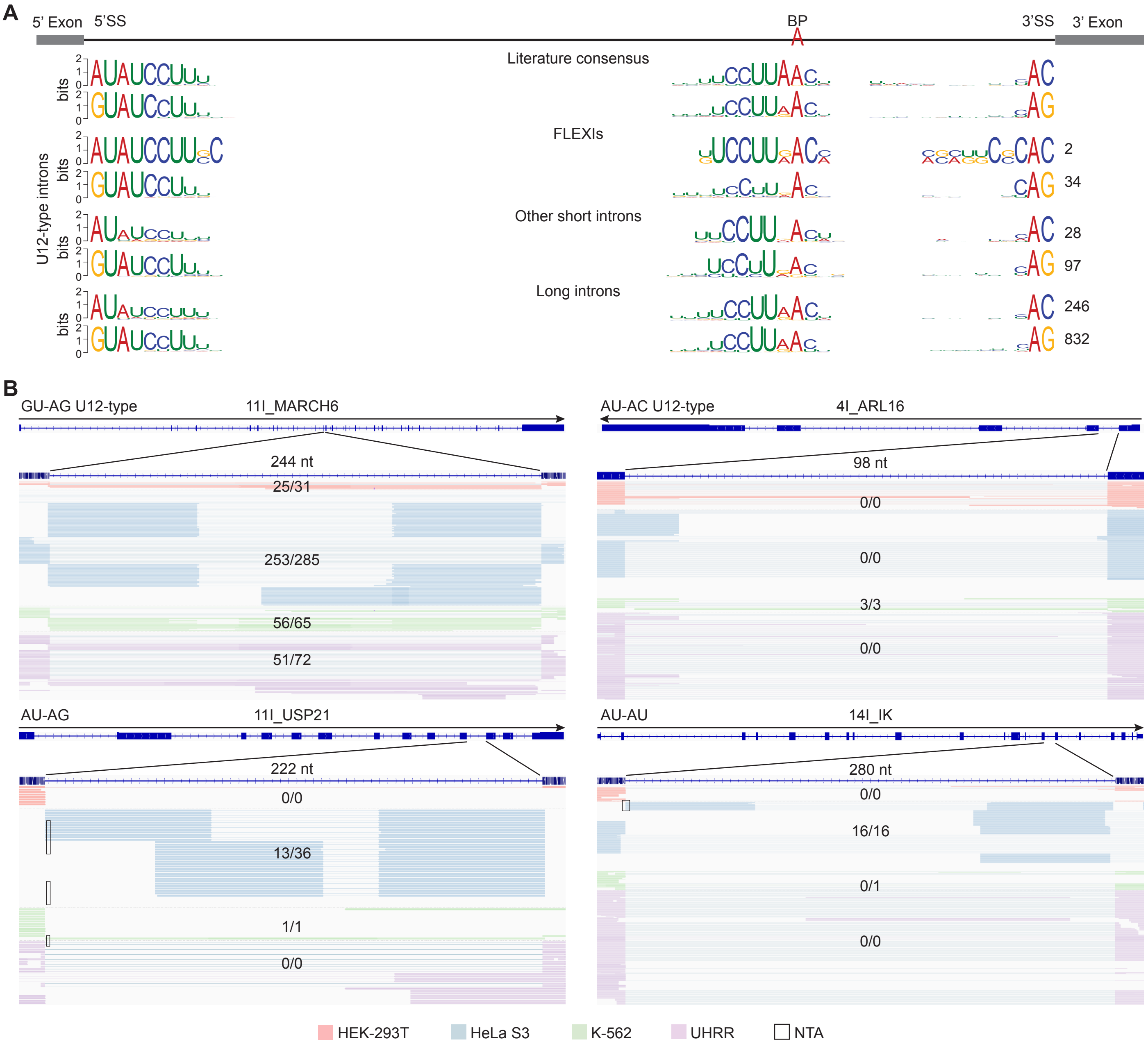

S4 Fig. FLEXI RNAs corresponding to U12-type introns or introns with non-GU-AG 5'- and 3'-splice sites. **(A)** 5'- and 3'-splice sites (5'SS and 3'SS, respectively) and branch-point (BP) consensus sequences of GU-AG and AU-AC U12-type FLEXIs in a combined datasets for the 4 cellular RNA samples (HEK-293T, HeLa S3, K-562, and UHRR) compared to literature consensus sequences for human U12-type introns (top) (28, 30) and consensus sequences for other U12-type short (≤300 nt) and long (>300 nt) introns in the same combined datasets. The number of introns for each consensus sequence is indicated to the right of the sequence. **(B)** IGV screenshots showing read alignments for U12-type and non-canonical splice-site FLEXI RNAs in RNA samples from different cell lines. FLEXIs are named at the top above an arrow indicating the 5' to 3' orientation of the RNA with the tracks below showing gene annotations (exons, thick lines; introns, thin lines) followed by alignments of FLEXI reads in different cellular RNA samples color coded by sample type. The length of the intron that is the focus of each panel is shown above the read alignments, and the fraction of FLEXI reads (continuous reads that began and ended within 3 nt of the annotated 5'- and 3'-splice sites) for that intron in different cell lines is shown within the read alignments. Reads were down sampled to a maximum of 100 for display in IGV. NTA, non-templated nucleotides added to the 3' end of cDNAs during TGIRT-seq library preparation.
